# Functional identification of two cytochrome P450 enzymes involved in diterpenoid oxidation in *Ginkgo biloba*

**DOI:** 10.3389/fpls.2026.1943006

**Published:** 2026-09-16

**Authors:** Lu Chen, Yue Zhang, Zhen Fan, Ying Wang, Ying Li, Qiang Zhou, Guo-Qiang Lin, Gong-Li Tang, Ying Zhou, Sheng Wu

**Affiliations:** 1 College of Pharmacy, Guizhou University of Traditional Chinese Medicine, Guiyang, China; 2 Innovation Research Institute of Traditional Chinese Medicine, Shanghai University of Traditional Chinese Medicine, Shanghai, China; 3 State Key Laboratory of Chemical Biology, Shanghai Institute of Organic Chemistry, Chinese Academy of Sciences, Shanghai, China

**Keywords:** biosynthetic pathway, CYP7005C, CYP720B, cytochrome P450, diterpene, *Ginkgo biloba*, *Saccharomyces cerevisiae*

## Abstract

*Ginkgo biloba* is a globally renowned medicinal plant. Ginkgo produces structurally unique diterpene metabolites, of which ginkgolides are the most representative bioactive compounds. Although the ginkgo genome contains a large and diverse family of cytochrome P450s (CYPs), only a few CYPs involved in the early steps of ginkgolide biosynthesis have been identified so far. As a result, the contribution of CYP-mediated oxidation to the diversity of ginkgo’s diterpene metabolism remains poorly understood. In this study, we used a constructed diterpenoid-producing yeast chassis for rapid functional screening and identified two cytochrome P450 oxidases from *G. biloba*: Gb_04669 (GbCYP7005C1) and Gb_33836 (CYP720B2). Gb_04669 catalyzed the oxidation of abietane-type diterpenoids, yielding 2*α*-hydroxyabietadiene and ginkgosinoic acid A. On the other hand, Gb_33836 catalyzed the C-19 oxidation of labdane-related alcohol substrates, producing isocupressic acid at a titer of 211.24 ± 9.98 μg/L in the engineered yeast strain. The contrasting product profiles of these two enzymes demonstrate the distinct catalytic activities of the CYP7005C and CYP720B families in *G. biloba* toward different diterpene scaffolds and extend our knowledge of P450-mediated early diterpenoid oxidation in gymnosperms. Our study expanded the substrate profile of CYP720Bs by showing that the ginkgo CYP720B acts on bicyclic diterpenes. This is different from previously characterized conifer CYP720Bs, which act on tricyclic diterpenes. These results provide new insights into the evolutionary diversification of this enzyme family. Collectively, this study highlights the value of the engineered yeast chassis for accelerating the discovery of plant diterpenoid oxidases and provides new enzymatic tools for the future biosynthetic production of structurally diverse oxidized diterpenoids.

## Introduction

*Ginkgo biloba* is an ancient relict gymnosperm species. It belongs to the Ginkgoaceae family and has been widely studied for its medicinal value. *G. biloba* leaf extract is used to treat conditions such as ischemic heart and brain disease, arteriosclerosis, asthma, and rheumatic diseases (Liu et al., 2022). Studies indicate that it may alleviate the symptoms of Alzheimer’s disease and minor circulatory disorders (Kulić et al., 2022). The standardized *G. biloba* extract EGb 761 contains 24% flavone glycosides and 6% terpenoids (3.1% ginkgolides A, B, and C, and 2.9% bilobalide) (Sabater-Jara et al., 2013). The growing demand for EGb 761 has stimulated research into the sustainable production of its active terpenoid components and related biosynthetic studies. *G. biloba* contains unique terpene trilactones (TTLs), comprising diterpenoid ginkgolides and the sesquiterpenoid bilobalide (Zeng et al., 2013). While ginkgolides are derived from *G. biloba*, the extraction of terpenoids from *G. biloba* leaves is not cost-effective. This has led to research into biosynthetic pathways for these compounds.

Ginkgo diterpenoid biosynthesis can be divided into three stages: upstream terpenoid precursor supply, diterpene skeleton formation, and oxidative post-modification of the diterpene skeleton (Bathe and Tissier, 2019; Du et al., 2025; Schepmann et al., 2001). The biosynthesis of diterpenoid is initiated with the methylerythritol phosphate (MEP) pathway. First, the enzyme geranylgeranyl diphosphate synthase (GGPPS) catalyzes the condensation of isopentenyl diphosphate (IPP) and dimethylallyl diphosphate (DMAPP) to form geranylgeranyl diphosphate (GGPP) (Gong et al., 2005). In labdane-type diterpene biosynthesis, skeleton formation requires both class II and class I diterpene synthase (diTPS) activities, either as separate enzymes or within bifunctional class II/I diTPSs (Peters, 2010; Gao et al., 2021). GbLPS belongs to the bifunctional diTPSs, in which the class II active site first cyclizes GGPP to a (+)-copalyl diphosphate [(+)-CPP] intermediate, followed by ionization, cyclization, and rearrangement mediated by the class I active site, generating levopimaradiene and related diterpene skeletons (Cyr et al., 2007). This mixed substrate background creates a challenge for assigning downstream cytochrome P450 (CYP) activities, because different CYP450s may act on structurally related but distinct diterpene substrates (**Figure 1A**).

**Figure 1:**
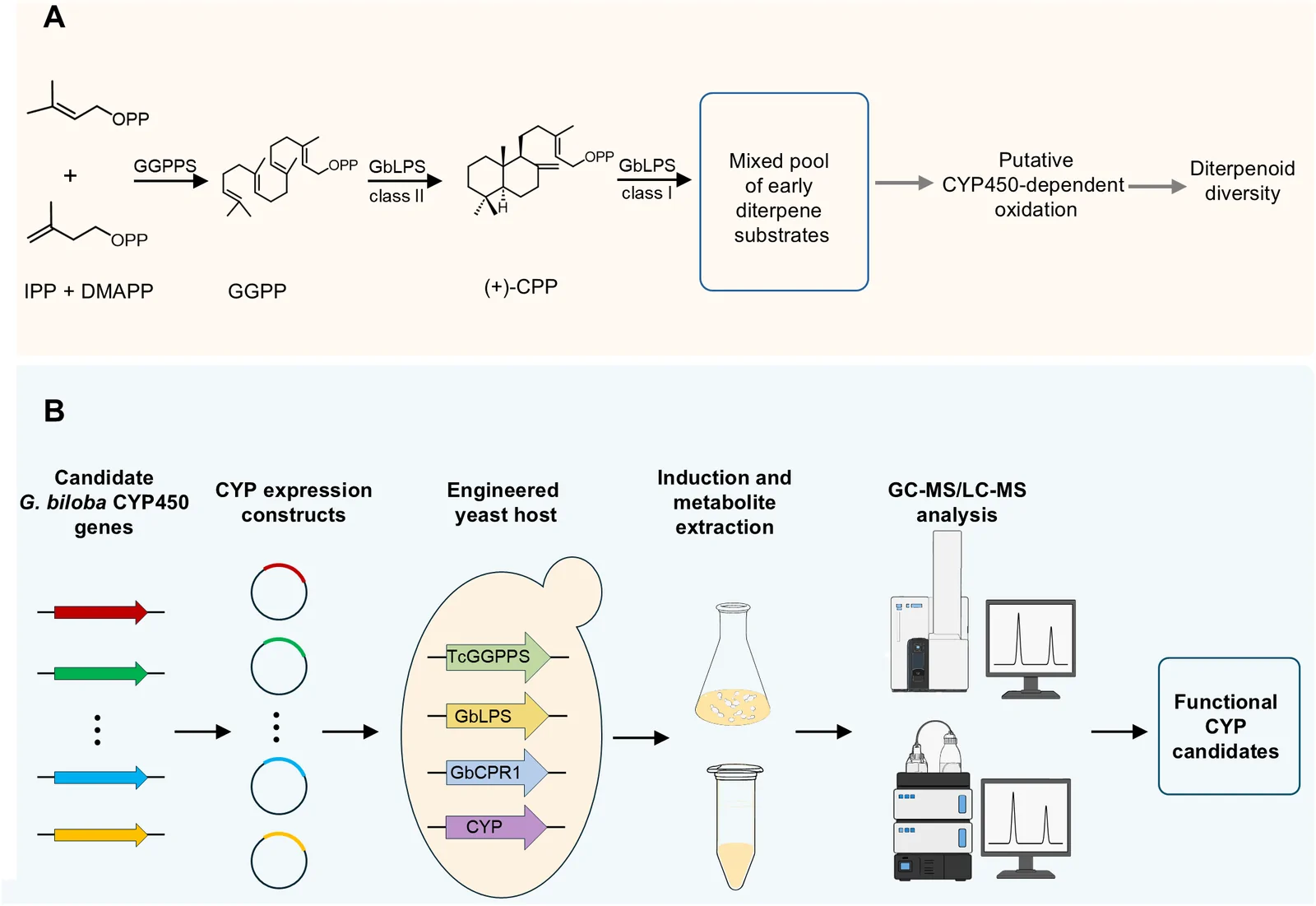
Conceptual framework and engineered-yeast screening strategy for early diterpenoid oxidation in Ginkgo biloba. **(A)** Geranylgeranyl diphosphate synthase (GGPPS) catalyzes the formation of geranylgeranyl diphosphate (GGPP) from isopentenyl diphosphate (IPP) and dimethylallyl diphosphate (DMAPP). The class II active site of bifunctional GbLPS cyclizes GGPP to (+)-copalyl diphosphate [(+)-CPP], which is subsequently converted by its class I active site into a mixed pool of early diterpene substrates. These substrates may undergo CYP450-dependent oxidation, contributing to diterpenoid diversity. **(B)** Candidate G. biloba CYP450 genes were individually introduced into an engineered yeast host expressing TcGGPPS, GbLPS, and GbCPR1. Following induction and metabolite extraction, product profiles were analyzed by GC–MS and LC–MS to identify CYP candidates with detectable activity in yeast.

Although the CYPs involved in ginkgo terpenoid biosynthesis have been analyzed and screened, the functional characterization of CYPs involved in ginkgo diterpenoid oxidation remains limited. Recently, Forman and collaborators found that the *G. biloba* CYP450 enzyme starts the biosynthesis of ginkgolides. They identified five CYP450 genes (GbCYP7005C1, GbCYP7005C2, GbCYP7005C3, GbCYP867K1, and GbCYP867E38) surrounding the terpene synthase gene (GbLPS), suggesting these genes form a biosynthetic gene cluster (Forman et al., 2022). Three CYP450 genes belong to the CYP7005 subfamily, while the other two are in the CYP867 subfamily with unknown functions. The functions of these genes were determined through heterologous expression in tobacco and *Saccharomyces cerevisiae* (Forman et al., 2022). Genomic analysis shows that the genes for ginkgolide biosynthesis are clustered on different chromosomes. The GbLPS gene is located on chromosome 5, alongside several CYP450 genes, suggesting that they may work together in the biosynthesis of ginkgolides (Liu et al., 2022). Forman et al. have characterized the functions of three CYP450 genes located on this chromosome (Forman et al., 2022). Similar clusters of CYP450 genes and terpenoid biosynthesis genes have been observed on other chromosomes as well. Consequently, further investigation into the functions of these CYP450 genes is essential.

To investigate how *G. biloba* CYP450 enzymes contribute to early diterpenoid oxidative diversification, we developed an engineered *S. cerevisiae* platform that supplies a pool of diterpene substrates and used it to screen candidate CYP450s. Our study focuses on identifying CYP450-dependent oxidative modifications acting on early-stage diterpene skeletons and comparing the product profiles generated by distinct candidate CYP450s. Through this approach, we aim to systematically survey the oxidative diversification of core diterpene scaffolds in ginkgo and to characterize the catalytic functions of CYP450 enzymes involved in this process (**Figure 1B**).

## Results

### Engineering yeast enhances production of diterpene olefins derived from GbLPS

To identify potential CYP450 enzymes, it is necessary to engineer strains that can produce high yields of diterpene skeletons. We cloned the codon-optimized GbLPS gene into a yeast expression vector, using the strong and constitutive P_GPD_ promoter to drive expression. The expression plasmid was then transformed into the CEN.PK2-1D yeast strain, resulting in the strain yCL1 (**Supplementary Table S1**). The product was extracted and analyzed by gas chromatography-mass spectrometry (GC–MS). However, only a negligible amount of the diterpene skeleton was detected with this configuration. To increase diterpene skeleton synthesis, we engineered a yeast strain to produce GbLPS-derived diterpene olefins for CYP450 screening. The platform features three key modifications. First, TcGGPPS from *Taxus cuspidata* is co-expressed to enhance the supply of GGPP, the direct precursor for diterpene cyclization. Second, we expressed the previously engineered GbLPS variant T79LPS^M593I/Y700F^, which lacks the N-terminal 79 amino acids and carries the M593I and Y700F substitutions previously reported to enhance levopimaradiene production in yeast (Liu et al., 2018). Hereafter, this engineered variant is referred to as GbLPS. Third, the genes were placed under galactose-inducible GAL promoters and expressed in a yeast strain with enhanced terpene precursor supply, resulting in strain yCL2 (**Supplementary Table S1**).

Using this reconfigured platform, up to five different diterpenoid products were detected in the yeast strain expressing TcGGPPS/GbLPS. These products include Sandaracopimaradiene (peak 1), Palustradiene (peak 2), Levopimaradiene (peak 3), Dehydroabietadiene (peak 4), and Abietadiene (peak 5). Peaks 1–3, 5 exhibited a mass-to-charge ratio (*m/z*) of 272, while peak 4 showed an *m/z* of 270 (**Figure 2**). Diterpenes were primarily detected in the yeast cell pellet, with almost no detectable levels found in the culture medium. No products were observed in the empty-vector control strains (**Figure 2**; **Supplementary Table S1**). This finding aligns with previous reports that GbLPS is a multifunctional diTPS capable of producing multiple diterpenoid skeletons, including levopimaradiene, abietadiene, and so on (Forman et al., 2022). Using engineering strategies, we developed yeast strains that produce diterpenoid skeletons for subsequent high-throughput screening of CYP450 oxidases.

**Figure 2:**
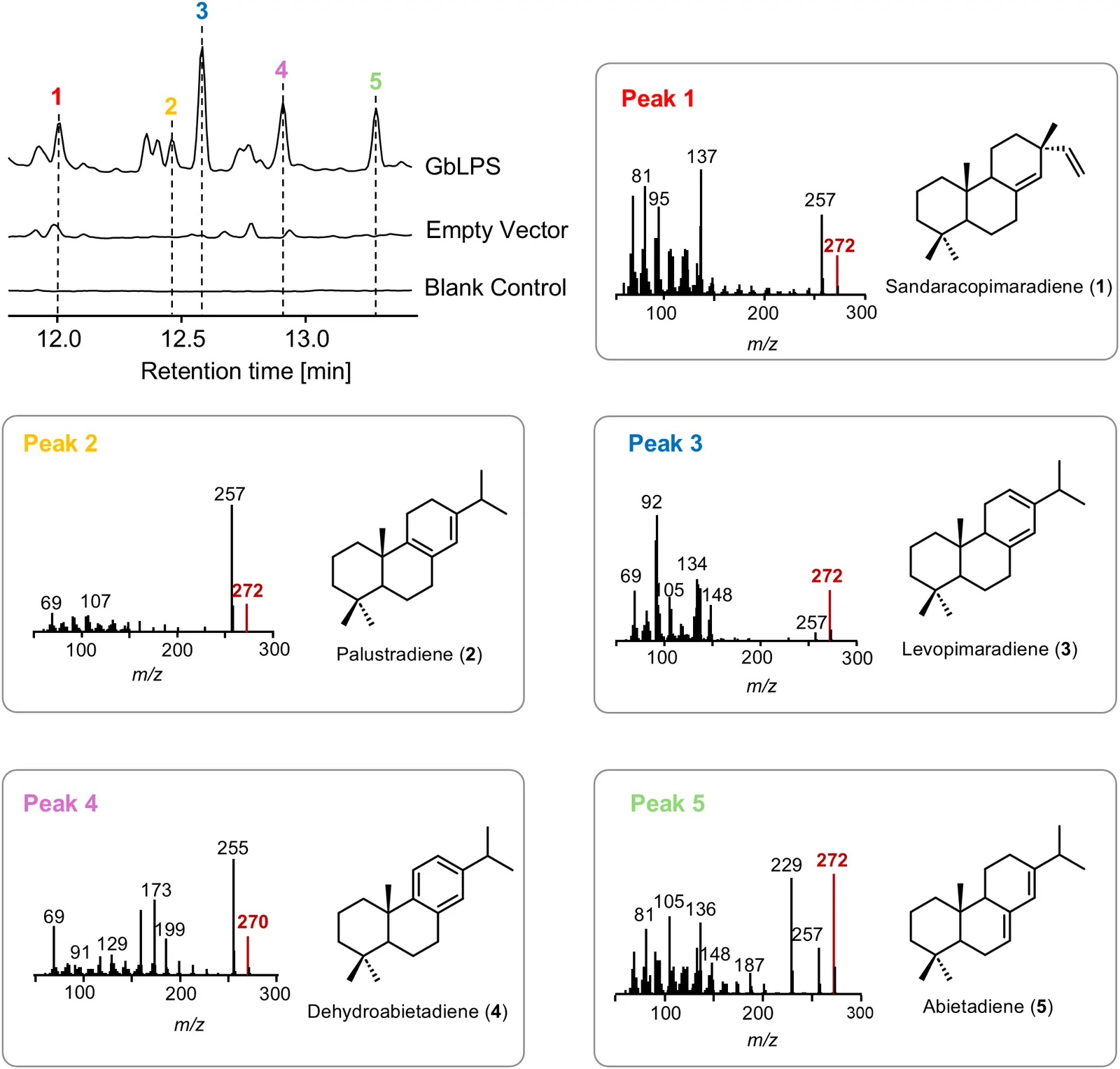
GC–MS analysis of diterpene olefins produced by GbLPS in yeast. Total ion chromatograms of extracts from yeast carrying TcGGPPS and GbLPS, the empty vector control, and the blank control are shown at the top left. Peaks 1–5 were specifically detected in the strain carrying GbLPS. EI mass spectra of the five peaks are shown together with the corresponding proposed diterpene structures. Diagnostic ions are labeled, with the molecular ions highlighted in red. The structures were assigned by comparing the observed EI mass spectra with the NIST 17 mass spectral libraries and reported diterpene mass spectra. Peaks 1–5 were assigned as sandaracopimaradiene (1), palustradiene (2), levopimaradiene (3), dehydroabietadiene (4), and abietadiene (5), respectively.

### Screening of candidate CYP450 genes identifies two diterpene oxidases

*G. biloba* genome and transcriptome data provide valuable insights into candidate CYP450 genes. Sun et al. analyzed 464 *G. biloba* CYP450 genes and identified 67 CYP450 enzymes that are potentially involved in diterpene biosynthesis in *G. biloba* (Sun et al., 2020). Additionally, five specific CYP450 genes (Gb_08057, Gb_08058, Gb_19877, Gb_19886, and Gb_36689) exhibit expression patterns similar to those of GbLPS and are believed to play a role in diterpene biosynthesis (Sun et al., 2020). To expand the candidate pool, we included the *G. biloba* CYP450 genes previously predicted to be associated with diterpene oxidation. We also considered CYP450 genes located near GbLPS on the same chromosome, as well as those distributed within ±10 genes of known mevalonate (MVA) pathway genes identified in the linkage analysis (**Supplementary Tables S2** and **S3**). We obtained these candidate CYP450 genes from publicly available *G. biloba* genome databases and performed phylogenetic analysis (**Supplementary Figure S1**). As a result, we selected 118 representative CYPs for further analysis (**Supplementary Table S4**).

A total of 118 candidate CYP450 genes were screened in an engineered yeast strain that contained GbLPS, TcGGPPS, and GbCPR1. Candidate CYP450s were selected when corresponding candidate-dependent peaks were reproducibly detected across independent cultures, absent from the corresponding controls, and associated with mass signals consistent with putative oxidized diterpenoid products. Based on this criterion, Gb_04669 and Gb_33836 produced candidate P450-dependent product peaks and were selected for detailed analysis. By contrast, the other candidate P450s did not generate detectable, reproducible peaks of oxidized diterpenoid products under the conditions tested.

### Yeast assays reveal Gb_04669-dependent oxidized abietadiene products

Gb_04669 shares 98% amino acid sequence identity with GbCYP7005C1, a CYP450 enzyme previously implicated in early oxidative metabolism related to ginkgolides (Forman et al., 2022). For this reason, Gb_04669 was prioritized for functional analysis in the engineered yeast platform, where GbLPS and TcGGPPS jointly supplied a diterpene substrate pool.

LC–HRMS analysis of the Gb_04669-expressing strain revealed three product peaks: a minor product 6 with *m/z* 287.2376 [M+H]^+^, product 7 with *m/z* 303.2325 [M+H]^+^, and product 8 with *m/z* 289.2534 [M+H]^+^. These peaks were absent from negative controls (**Figures 3A, B**). Large-scale fermentation of the strain expressing Gb_04669/GbCPR1/TcGGPPS/GbLPS (yCL3, **Supplementary Table S1**) enabled purification of products 7 and 8. Product 7 was identified as ginkgosinoic acid A based on high-resolution mass spectrometry (HRMS) data and agreement with previously reported ^1^H nuclear magnetic resonance (NMR) values (**Supplementary Figure S4**, **Supplementary Table S7**). Product 8 was identified as 2*α*-hydroxyabietadiene by HRMS and one- and two-dimensional NMR analyses, including HSQC, HMBC, and NOESY correlations (**Supplementary Figures S5–11**; **Supplementary Table S8**). Together, these results indicate that Gb_04669 was associated with the formation of multiple oxidized diterpenoid products from the GbLPS-supplied substrate pool, allowing possible reaction relationships among the detected products to be proposed (**Figure 3C**). To enhance the yield of GbCYP products in *S. cerevisiae*, we tested CPRs from various plant sources, including AtCPR1 from *Arabidopsis thaliana* and GbCPR1 and GbCPR2 from *G. biloba* (yCL3-yCL5, **Supplementary Table S1**). We found that native GbCPR1 exhibited the highest activity (**Supplementary Figure S2**), so subsequent tests all used GbCPR1.

**Figure 3:**
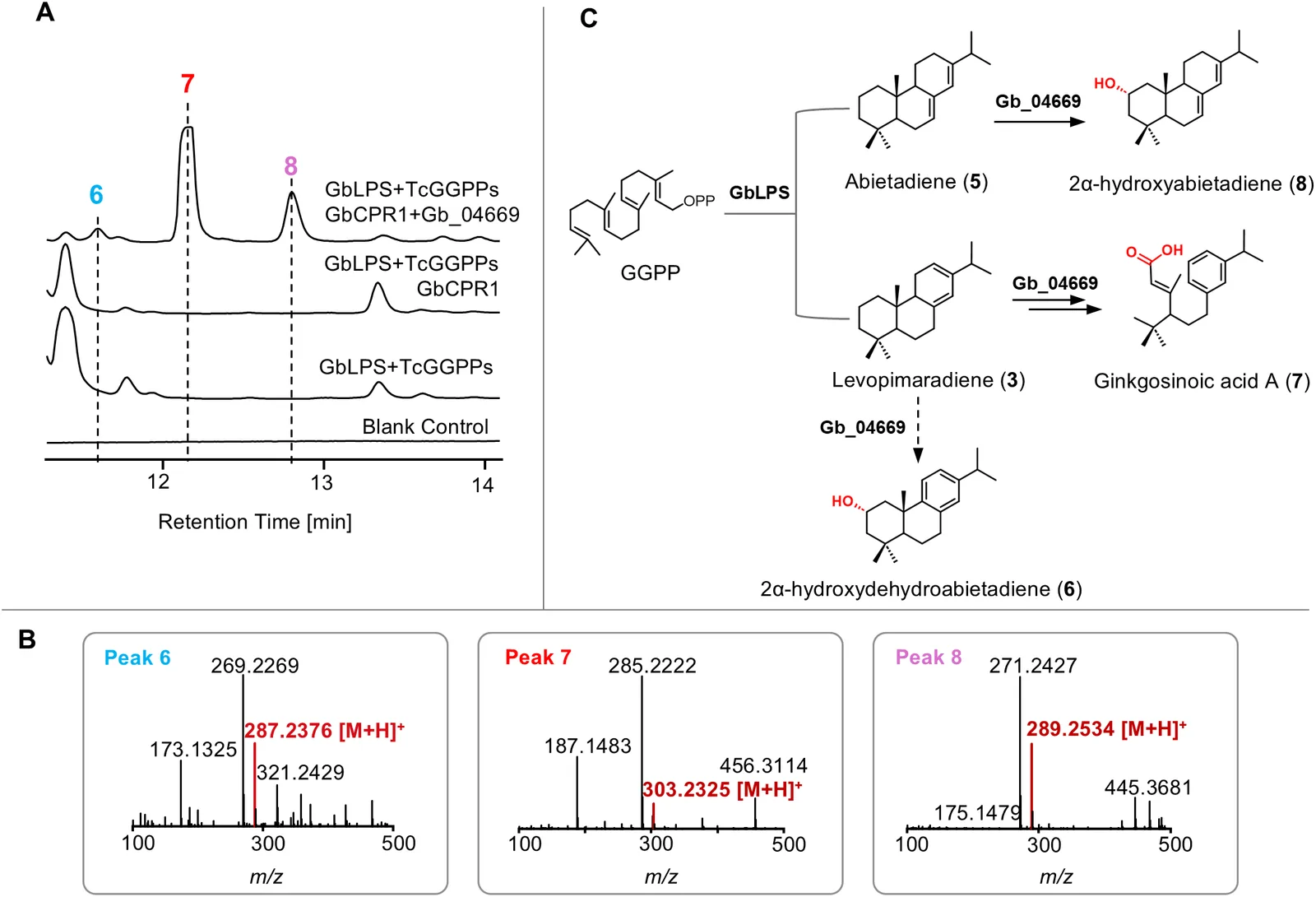
Detection of Gb_04669-dependent oxidized diterpenoid products in engineered yeast. **(A)** LC–PDA chromatograms of extracts from yeast strains carrying different combinations of genes: GbLPS + TcGGPPS + GbCPR1 + Gb_04669, GbLPS + TcGGPPS + GbCPR1, GbLPS + TcGGPPS, or no introduced diterpenoid pathway. Peaks 6–8 were specifically detected or strongly increased when Gb_04669 was introduced into the GbLPS + TcGGPPS + GbCPR1 background. **(B)** MS spectra of peaks 6–8. Protonated molecular ions are highlighted in red. **(C)** Proposed product assignments and reaction relationships for Gb_04669 in the yeast diterpenoid substrate background. Peaks 7 and 8 were assigned as ginkgosinoic acid A and 2α-hydroxyabietadiene, respectively, whereas peak 6 was tentatively assigned as a putative 2α-hydroxydehydroabietadiene.

The NMR data for product 8 differ from the previously published data for 2*α*-hydroxylevopimaradiene. Our results support the assignment of product 8 as a hydroxylated abietadiene derivative (Forman et al., 2022). The NOE correlation between H-2 and the C-20 methyl group further supported the stereochemical assignment of the C-2 hydroxyl group in 2*α*-hydroxyabietadiene. Whole-cell yeast assays revealed that the accumulation of product 8 was dependent on the expression of Gb_04669. Along with the detection of ginkgosinoic acid A in the same engineered strain, these data reveal a broader profile of Gb_04669-dependent oxidized products within the GbLPS-supplied substrate pool.

To further examine whether this activity could extend to other structurally related diterpenes with an abietane skeleton, GbLPS was replaced by a pair of genes responsible for synthesizing miltiradiene: SmCPS/SmKSL1 (both from *Salvia miltiorrhiza*) or SmCPS/CfTPS3 (from *S. miltiorrhiza* and *Coleus forskohlii*, respectively), generating strains yCL6 and yCL7 (**Supplementary Table S1**) (Bai et al., 2024). Under the same yeast expression conditions, no oxidation product associated with Gb_04669 expression was detected in either miltiradiene background. This result suggests that Gb_04669 does not act broadly on all related abietane diterpenes, and that the position of the double bond in the diterpene substrate may influence its catalytic activity. Therefore, although Gb_04669 can be associated with the oxidation of multiple products generated by GbLPS in yeast, it still retains a degree of substrate selectivity.

### Gb_33836 is associated with isocupressic acid formation in engineered yeast

We generated a set of yeast strains for CYP450 screening. The screening results revealed that Gb_33836 exhibits catalytic activity when co-expressed with TcGGPPS, GbLPS, and GbCPR1 (yCL8, **Supplementary Table S1**). Two new products accumulated in the Gb_33836-expressing strain (**Figure 4A**). These metabolites were identified as product 10 with an *m/z* of 321.2426 [M+H]^+^ (**Figure 4D**) and product 11 with an *m/z* of 349.2737 [M+H]^+^ (**Figure 4D**), suggesting that they may be oxidation products of diterpenes. Both compounds were detected in cell extracts rather than in the culture medium, indicating intracellular accumulation of the oxidized diterpenoids.

**Figure 4:**
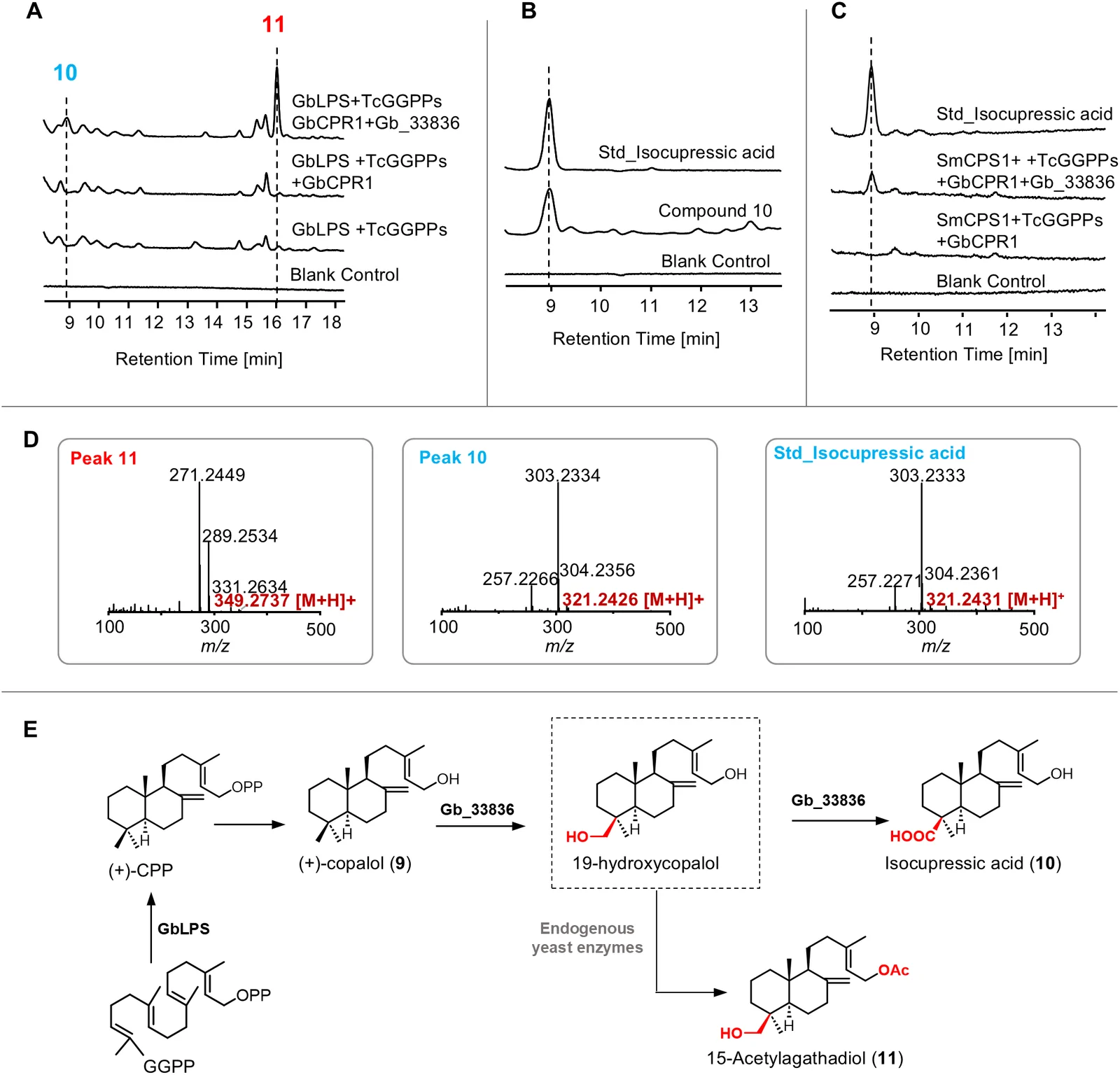
LC -PDA and MS analysis of Gb_33836-associated oxidation products in engineered yeast. **(A)** LC -PDA chromatograms at 190 nm of extracts from yeast strains carrying GbLPS + TcGGPPS + GbCPR1 + Gb_33836, GbLPS + TcGGPPS + GbCPR1, GbLPS + TcGGPPS, and Blank Control. Peaks 10 and 11 were specifically detected when Gb_33836 was introduced into the GbLPS + TcGGPPS + GbCPR1 background. **(B)** LC -PDA comparison of compound 10 with an authentic isocupressic acid standard. The matching retention time supported the assignment of compound 10 as isocupressic acid. **(C)** LC -PDA chromatograms at 190 nm of extracts from yeast strains carrying SmCPS + TcGGPPS + GbCPR1 + Gb_33836, SmCPS + TcGGPPS + GbCPR1, and Blank Control, together with the authentic isocupressic acid standard. A peak corresponding to isocupressic acid was specifically detected when Gb_33836 was introduced into the SmCPS + TcGGPPS + GbCPR1 background, further supporting that Gb_33836 acts on (+)-copalol (9). **(D)** MS spectra of peak 11, peak 10, and the authentic isocupressic acid standard, with the protonated molecular ions highlighted in red. Peak 10 showed the same protonated molecular ion as the standard at m/z 321.24, whereas peak 11 showed a protonated molecular ion at m/z 349.27. **(E)** Proposed reaction relationship for Gb_33836 in the GbLPS product background. GbLPS generates a (+)-CPP intermediate, which may be hydrolyzed by endogenous yeast enzymes to form (+)-copalol (9). Gb_33836 is associated with further oxidation of this compound to isocupressic acid (10), probably via C-19 oxidized intermediates. Additionally, 15-acetylagathadiol (11) is proposed to arise from further modification of a hydroxylated intermediate, potentially involving endogenous yeast enzymes.

Large-scale fermentation of the Gb_33836-expressing strain enabled the isolation and purification of two compounds. Product 10 was unambiguously identified as isocupressic acid based on NMR data, and comparison with an authentic standard (**Supplementary Figures S12**–**S14**; **Supplementary Table S9**). All observed chemical shift values were in excellent agreement with literature data (Qiao et al., 2019; Manley-Harris et al., 2025). The structural assignment was further confirmed by comparison with an authentic standard, which showed identical retention time in high-performance liquid chromatography (HPLC) and HRMS data (**Figures 4B, D**). Based on analyses by one- and two-dimensional NMR techniques, as well as HRMS (*m/z* 349.2737 [M+H]^+^), product 11 was identified as the esterification product of hydroxylated copalol and was named 15-acetylagathadiol (**Supplementary Figures S15**–**S21**; **Supplementary Table S10**), which has been found in *Cryptomeria japonica* (Wen-Chiung et al., 1994). Products 10 and 11 are both derived from (+)-copalol. Additionally, (+)-copalol is the alcohol product formed by the dephosphorylation of (+)-copalyl diphosphate (CPP). The compound (+)-CPP is typically synthesized through the action of (+)-copalyl diphosphate synthase on GGPP (Gao et al., 2021). The mechanism by which (+)-CPP undergoes non-selective hydrolysis to produce (+)-copalol is not fully understood and may involve endogenous phosphatases present in yeast cells. Given that GbLPS is a bifunctional class II/I diTPS that proceeds through a (+)-CPP intermediate before producing levopimaradiene and related diterpene skeletons (Cyr et al., 2007), we propose that GbLPS functions similarly to (+)-CPP synthase, as this bifunctional enzyme also produces the (+)-CPP intermediate during the synthesis of levopimaradiene/abietadiene (Schepmann et al., 2001). To further clarify the substrate context associated with Gb_33836 activity, the known (+)-CPP synthase SmCPS was introduced into the yeast platform to generate a simplified (+)-CPP derived substrate background (yCL9, **Supplementary Table S1**) (Cui et al., 2015). GC–MS analysis showed that the SmCPS-expressing strain produced peak 9, which was assigned as (+)-copalol based on comparison with the NIST 17 mass spectral database and the reported spectrum by Gao et al. (2023) (**Supplementary Figure S3**). After coexpression of SmCPS/TcGGPPS/Gb_33836/GbCPR1 (yCL10, **Supplementary Table S1**), a peak with the same retention time as the authentic isocupressic acid standard was detected (**Figure 4C**). This peak was not observed in the negative control (SmCPS/TcGGPPS/GbCPR1) or the blank control strain (**Supplementary Table S1**). These results further confirm that Gb_33836 acts on (+)-copalol.

These findings suggest that Gb_33836 can catalyze consecutive oxidations at the C-19 position of (+)-copalol, resulting in the formation of an alcohol and its corresponding carboxylic acid. The aldehyde intermediate is unstable and was not detected in yCL8 and yCL10 under our experimental conditions. In the strain that produces mixed diterpene olefins, Gb_33836 shows activity only towards bicyclic diterpenes, indicating a substrate preference of Gb_33836 for the bicyclic parent structure. Based on these observations, Gb_33836 was identified as the enzyme responsible for the synthesis of isocupressic acid (**Figure 4E**). The strain produced isocupressic acid at a titer of 211.24 ± 9.98 μg/L in shake-flask cultivation (mean ± SD, n = 3).

### Tissue expression of Gb_04669 and Gb_33836 in *Ginkgo biloba*

Transcripts of both Gb_04669 and Gb_33836 were detected in leaf, sarcotesta, and stem tissues from all three *G. biloba* individuals (**Supplementary Figure S22**; **Supplementary Table S11**). Both genes showed their highest measured transcript abundance in stem. These data provide plant expression context for the functional observations in yeast.

### Evolutionary divergence of CYP720B enzymes in gymnosperms

Gb_33836 belongs to the CYP720B subfamily, which is vital for the biosynthesis of diterpene resin acids (DRAs) (Hamberger et al., 2011). DRAs are specialized metabolic products known as coniferous resin defense compounds, which accumulate in oleoresin produced by the CYP720B family (Hamberger et al., 2011). CYP720B genes are present in various conifer genomes, with only a few identified: *Pinus taeda* PtCYP720B1, *Picea sitchensis* PsCYP720B4 (Hamberger et al., 2011) and *Pinus banksiana* PbCYP720B2/B12 (Geisler et al., 2016). The first two enzymes catalyze a three-step oxidation process that converts diterpene olefins, such as levopimaradiene and dehydroabietadiene, into DRAs. This reaction is similar to the conversion of *ent*-kaurenoic acid from *ent*-kaurene in gibberellin biosynthesis (Hamberger et al., 2011). The third enzyme, PbCYP720B2/B12, catalyzes the conversion of 13-hydroxy-8(14)-abietene—a product of the levopimaradiene/abietadiene synthase (LAS)—into the corresponding C-18 carboxylic acid (Geisler et al., 2016). Therefore, the combination of diTPS and CYP720B enzymes produces a variety of defense compounds.

The *G. biloba* genome includes at least twelve CYP720B genes. Two of these genes, Gb_33836 (isocupressic acid synthase) and Gb_33837, share 79% sequence identity, while the other CYP720B genes show lower identity with Gb_33836, ranging from 59% to 66%. Forman et al. found that when GbCYP720B33 (Gb_36690) was co-expressed with GbLPS, two minor products were produced (Forman et al., 2022); however, they were unable to purify sufficient quantities of these products for structural identification. Additionally, GbCYP720B31 (Gb_08057) hydroxylates ginkgosinoic acid C, forming ginkgolactone C, which is then further oxidized to ginkgolactone D (Forman et al., 2022).

We compared the CYP720B genes among various Pinaceae species and *G. biloba*. Phylogenetic analysis revealed that these CYP720B genes can be divided into four distinct clades (**Figure 5A**). PsCYP720B4 and PbCYP720B2/B12 are located in clades I and II, respectively. Gb_33836 was placed in clade IV. Although the function of most clade III members remains unknown, our phylogenetic results imply potential differences in substrate preferences among the enzymes in clades I–IV; however, this inference requires direct experimental verification through comparative biochemical characterization. Nevertheless, this finding further validates the existing classification of these four clusters. The combined action of diTPSs and CYP720B generates a variety of oxidized diterpenoid compounds, including isocupressic acid. Based on the distinct phylogenetic positions, we speculate that the CYP720B genes from *G. biloba* and Pinaceae may contribute to distinct product profiles (**Figure 5B**). Notably, CYP720B appears to be gymnosperm-specific, and further biochemical studies are needed to clarify its evolutionary divergence and functional roles.

**Figure 5:**
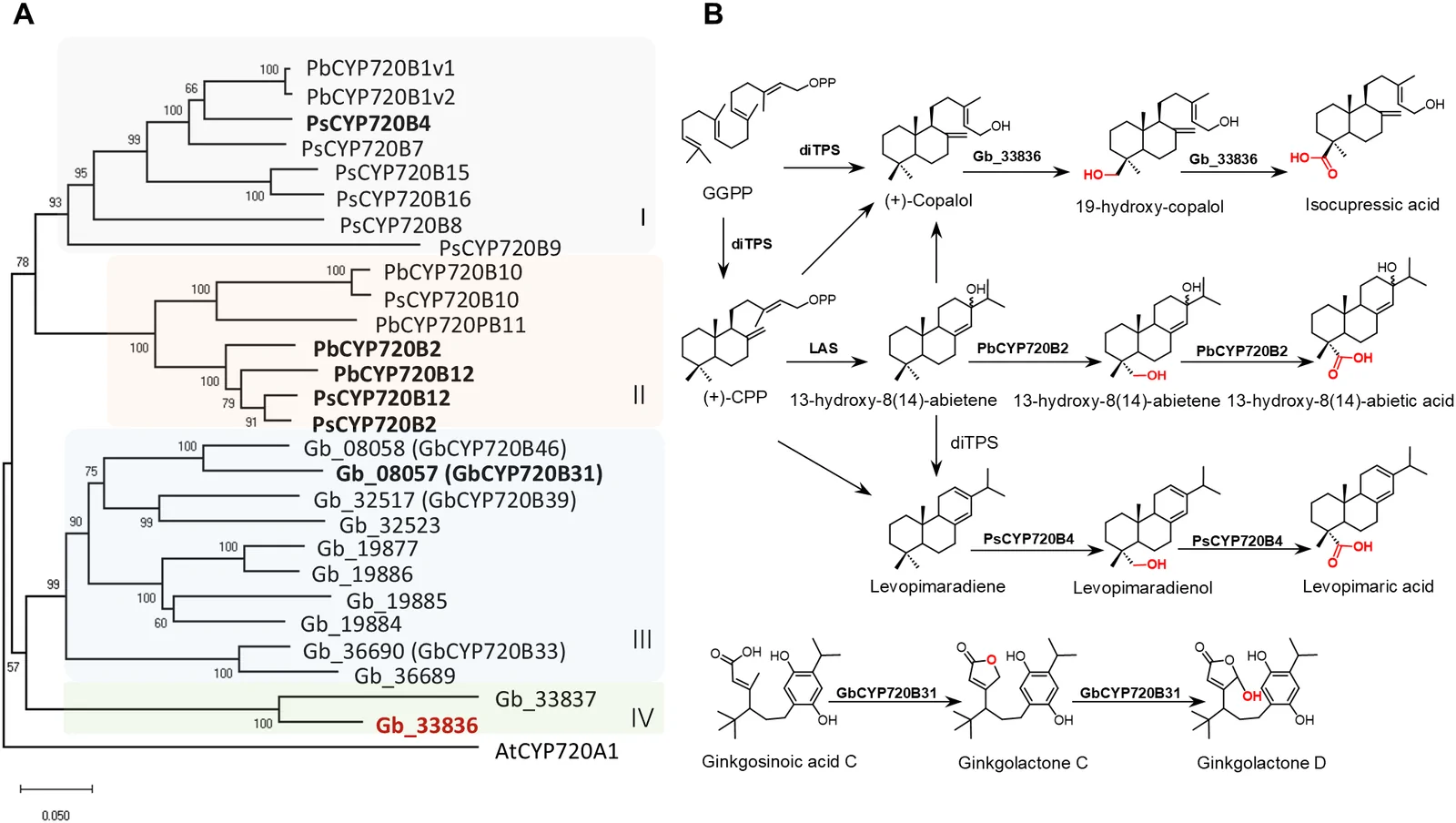
Phylogenetic placement of Gb_33836 within gymnosperm CYP720B enzymes and comparison of reported oxidation profiles. **(A)** Phylogeny of the gymnosperm-specific CYP720B family from Pinaceae plants and G. biloba. CYP720Bs are classified into four clades (I to IV) based on phylogenetic tree analyses. Previously identified CYP720B enzymes are shown in bold black font. The CYP720B enzyme discovered in this study are highlighted in red. Arabidopsis thaliana AtCYP720A1 is used as an outgroup. At, Arabidopsis thaliana; Pb, Pinus banksiana; Ps, Picea sitchensis, Gb, Ginkgo biloba. **(B)** Diagram of reactions catalyzed by CYP720B enzymes in diterpenoid biosynthesis. Clade I enzyme PsCYP720B4 catalyzes a three-step oxidation reaction, converting diterpene olefins like levopimaradiene and dehydroabietadiene into DRAs; Clade II enzyme PbCYP720B2/B12 facilitates the conversion of 13-hydroxy-8(14)-abietene into the corresponding C-18 carboxylic acid; Clade III enzyme GbCYP720B31 facilitates the oxidation of intermediates involved in the biosynthesis of ginkgolides; Clade IV enzyme Gb_33836 catalyzes consecutive oxidations at the C-19 position of (+)-copalol, resulting in the formation of an alcohol and the corresponding acid.

## Discussion

The genome of *G. biloba* encodes a large cytochrome P450 family, yet the specific functions of the vast majority of its members in diterpene metabolism remain largely unknown. In this study, we used a constructed diterpenoid-producing yeast chassis to functionally screen 118 P450 candidate genes from *G. biloba* and successfully identified two oxidases with clear catalytic activities—Gb_04669 and Gb_33836. These two enzymes not only act on distinctly different diterpene scaffolds but also represent either new members or novel functional extensions of their respective families, and provide key experimental evidence for understanding the molecular basis of diterpenoid oxidative diversity.

In engineered yeast, Gb_04669 catalyzed the formation of 2*α*-hydroxyabietadiene, and ginkgosinoic acid A was also detected. The detection of ginkgosinoic acid A is consistent with the activity of GbCYP7005C1 reported by Forman et al. The proposed mechanism for ginkgosinoic acid A starts with the hydroxylation of levopimaradiene at the C2 position. This is followed by the opening of rings A and B, which leads to the formation of ginkgosinoic acid A (Forman et al., 2022). Unlike the previous study, however, we did not detect 2*α*-hydroxylevopimaradiene. Instead, we observed the accumulation of 2*α*-hydroxyabietadiene. While no oxidized products were detected in a miltiradiene-producing yeast background, this suggests that Gb_04669 has substrate flexibility but does not extend to all abietane-type substrates. Gb_04669 may therefore preferentially recognize abietane-type substrates with specific structural features, although its precise substrate range remains to be determined. Similar C-2 oxidation reactions have been reported for enzymes from other CYP families. For example, CYP71BE52 from *Salvia pomifera* catalyzes the C-2 hydroxylation of ferruginol (Ignea et al., 2016), whereas CYP72D19 from *Tripterygium wilfordii* catalyzes the C-2 hydroxylation of dehydroabietic acid (Gao et al., 2023). The CYP7005 enzyme family demonstrates significant activity and is essential for the structural diversity of diterpene products. Together, these findings indicate that C-2 oxidation is a modification that appears across different CYP families and contributes to the structural diversification of abietane-type diterpenoids.

The second finding is that Gb_33836 was confirmed to catalyze the C-19 oxidation of the bicyclic labdane-type substrate (+)-copalol to produce isocupressic acid, with a titer of 211.24 ± 9.98 μg/L in the yeast chassis. The SmCPS-based assay provided further *in vivo* evidence in yeast that (+)-copalol is the substrate of Gb_33836, and that the C-19 oxidation likely proceeds via a sequential alcohol-aldehyde-acid pathway. Gymnosperms produce a variety of diterpenoid metabolites. For example, in pine and spruce species, DRAs act as primary defense compounds (Ma et al., 2019). Isocupressic acid, also belonging to the labdane resin acids group, has been identified in coniferous trees from the Pinaceae and Cupressaceae families. This includes species such as Ponderosa pine (*Pinus ponderosa*) (Gardner et al., 1994; Hlaili et al., 2025), *Thuja standishii* (Iwamoto et al., 2003), *Juniperus oblonga* (Qiao et al., 2019), and *Cupressus macrocarpa* (Wang et al., 2004). From an ecological function perspective, isocupressic acid has demonstrated phytotoxic activity in various species (Hlaili et al., 2025); meanwhile, its pharmacological activity has also attracted attention, particularly its role as an abortifacient in late-term cattle pregnancy (Wang et al., 2004). The carboxylic acid group is important for the phytotoxic activity of the compound (Hlaili et al., 2025), suggesting that the sequential oxidation at C-19 is not only a key step in the biosynthesis of this compound but may also be an important structural determinant of its biological activity and toxicity. Recently, Lin et al. engineered *Streptomyces* hosts to produce *ent*-isocupressic acid. They introduced *ent*-CPP synthase (a class II diterpene synthase, eCDPS) and a cytochrome P450 monooxygenase (eAOx). *Ent*-CPP synthase generates *ent*-copalol, which is the hydrolysis product of *ent*-CPP, while eAOx oxidizes the C-19 methyl group of labdane diterpenes (Lin et al., 2025). A BLAST analysis shows that eAOx is a CYP450 protein derived from *Streptomyces*; however, it shares only 23.44% sequence identity with Gb_33836. Although the two enzymes catalyze similar types of reactions, they act on substrates of different stereochemistry and produce the corresponding stereoisomers, suggesting a distant evolutionary relationship and distinct stereochemical selectivity. This contrast further highlights the uniqueness and research value of Gb_33836, as a plant‐derived member of the CYP720B family, in terms of substrate recognition and catalytic selectivity.

Characterized Pinaceae CYP720Bs, such as PtCYP720B1 and PsCYP720B4, catalyze consecutive C-18 oxidation of tricyclic diterpenes (such as abietane-, pimarane-, and dehydroabietane-type), converting diterpene olefins into DRAs (Hamberger et al., 2011; Geisler et al., 2016). Although individual CYP720Bs differ in their preference for diterpene precursors, they generally retain the core reaction feature of the C-18 methyl group on ring A. In contrast, *G. biloba* CYP720Bs display more diverse product profiles. GbCYP720B31, previously identified, acts on ginkgosinoic acid C, a structurally rearranged intermediate derived from a diterpenoid precursor. Whereas Gb_33836 in the present study supports the formation of isocupressic acid from (+)-copalol in engineered yeast. Thus, the substrates catalyzed by CYP720B enzymes include not only the tricyclic diterpenes commonly found in Pinaceae, but also ginkgosinoic acid C and bicyclic labdane-type compounds, indicating that this family possesses relatively broad substrate recognition and catalytic properties. This observation is consistent with the phylogenetic analysis, which categorized these enzymes into different CYP720B clades (**Figure 5A**). However, only a limited number of CYP720Bs have been characterized to date, and there has been no direct comparison of representative enzymes from different clades under identical experimental conditions. Consequently, the relationship between phylogenetic position and catalytic function within the CYP720B family requires further investigation.

The engineered yeast used in this study offers advantages for large-scale functional screening of plant P450s. The yeast-based approach avoids interference from plant endogenous metabolic networks. It enables rapid comparison of the activity of many candidate genes under a uniform metabolic background. This platform identifies CYP candidates that are compatible with the heterologous system. Of the 118 candidates screened, only two produced detectable products. This low hit rate is likely due to several factors. These include intrinsic enzyme activity, variable CYP expression in yeast, poor compatibility between CYPs and CPRs, and the absence of upstream, pathway-specific intermediates in yeast, which prevents downstream CYPs from recognizing their native substrates. Therefore, the absence of a detectable product for a given candidate does not necessarily mean that it lacks catalytic function in plants. Further screening rounds using the oxidized intermediates generated by the two positive candidates may reveal additional oxidases involved in diterpenoid metabolism.

## Conclusions

In summary, our engineered-yeast screening identified two *G. biloba* CYP450s, Gb_04669 and Gb_33836, that collectively illuminate early diterpenoid oxidative diversification in this species. Gb_04669 expands the known substrate scope of the CYP7005C family to multiple abietane-type skeletons, whereas Gb_33836 catalyzes the oxidation of a bicyclic labdane-type substrate ((+)-copalol) to yield isocupressic acid. This function is distinct from that of previously characterized Pinaceae CYP720B enzymes, which oxidize tricyclic diterpene substrates. This research provides new insights into the functional diversification of CYP7005C and CYP720B subfamilies. Additionally, further screening with a variety of oxidized substrates may uncover more CYP450 activities and help clarify the organization of diterpenoid metabolism.

## Methods

### Identification and prioritization of CYP450 candidates using bioinformatics

Candidate cytochrome P450 genes were collected from GinkgoDB, a genome database for *G. biloba*, and from published *G. biloba* CYP450 datasets (Gu et al., 2022). Candidate sequences were examined using homology searches, conserved domain analysis, and predicted protein features. Sequences were retained when they contained a conserved cytochrome P450 domain and characteristic P450 motifs, including the heme binding motif FxxGxRxCxG, the PERF motif, the K helix ExxR motif, and a predicted N-terminal membrane anchor region when present. Phylogenetic analysis was conducted in MEGA 11 using the maximum likelihood method, and the final tree was visualized and annotated using iTOL.

### Plasmid construction

The coding sequences used in this study were commercially synthesized by GENEWIZ (Jiangsu, China) and codon-optimized for expression in *S. cerevisiae* (**Supplementary Table S5**). GbLPS was expressed as the previously reported engineered variant T79LPS^M593I/Y700F^, which lacks the N-terminal 79 amino acids and carries the M593I and Y700F substitutions, and TcGGPPS was coexpressed to enhance geranylgeranyl diphosphate supply. GbCPR1 and candidate CYP450 genes were cloned into compatible pESC vectors by restriction enzyme mediated ligation or homologous recombination, with all candidate CYP450 genes placed under the GAL10 promoter. Plasmid configurations are listed in **Supplementary Table S6**. Recombinant plasmids were verified by Sanger sequencing of the vector insert junctions and selected coding regions before yeast transformation.

### Screening of candidate CYP450 enzymes in engineered yeast

A total of 118 candidate CYP450 genes were individually coexpressed with GbCPR1 in an engineered yeast strain containing GbLPS and TcGGPPS. For each candidate CYP450 assay, three independent transformants were cultivated and analyzed under identical conditions. Controls included the corresponding empty-vector strain, a strain expressing GbLPS and TcGGPPS, and a strain additionally expressing GbCPR1 without a candidate CYP450. All the expression products of candidate genes were initially screened by GC–MS, and selected strains showing candidate-dependent signals were further examined by LC–MS. Candidate-dependent products were prioritized based on the absence of corresponding peaks in controls. The putative products were further characterized by LC–PDA, LC–HRMS, and NMR spectroscopy.

### Yeast heterologous expression and cultivation

Recombinant plasmids were introduced into the engineered *S. cerevisiae* strain JCR27 using the Frozen EZ Yeast Transformation II kit (Zymo Research, USA). The strain genotype is listed in **Supplementary Table S1**. Transformants were selected on the appropriate synthetic dropout agar medium according to the plasmid markers and incubated at 30 °C for 2–4 days. A single colony was inoculated into 3 mL of the corresponding dropout medium containing 2% (w/v) glucose and cultivated at 30 °C and 220 rpm for 12–16 h. Thirty microliters of the preculture was then transferred into 3 mL of fresh glucose-containing dropout medium and cultivated for an additional 24 h. Cells were harvested at 3,000 rpm for 3 min at room temperature, washed with sterile water, and resuspended in 3 mL of the corresponding induction medium containing 2% (w/v) galactose and 2% (w/v) raffinose. Cultures were incubated at 30 °C and 220 rpm for 72 h.

### Metabolite extraction and sample preparation

After 72 h of cultivation, cells were harvested at 4,500 rpm for 10 min at room temperature. Because the target diterpenoids were predominantly cell associated, the culture supernatants were discarded. Each cell pellet was extracted with 400 µL of methanol and 100 µL of acetone in the presence of 50 µL of 0.5 mm glass beads. Cells were disrupted using a FastPrep-24 homogenizer (MP Biomedicals, Irvine, CA, USA) at 4.5 m/s for two 30 s cycles. After centrifugation at 13,000 rpm for 10 min, the supernatant was collected and evaporated to dryness in a vacuum centrifugal concentrator at 25 °C. Dried extracts were either immediately reconstituted for instrumental analysis or stored at −20 °C.

### Analysis of terpenoids

#### Gas chromatography-mass spectrometry analysis

The extract was reconstituted in 100 µL of ethyl acetate. The analysis was performed on a Shimadzu QP2020 GC-MS instrument equipped with a DB-5ms column (30 m × 0.25 mm, 0.25 µm). One microliter of sample was injected using splitless mode at 280 °C. Helium was the carrier gas. The oven temperature program was as follows: 90 °C for three minutes; increased to 200 °C at 25 °C/min (held for three minutes); increased to 290 °C at 15 °C/min (held for four minutes); and increased to 300 °C at 10 °C/min (held for five minutes). Mass spectra were obtained in full-scan mode over *m/z* 60–500 using electron ionization at 70 eV. The ion source and interface temperatures were 220 °C and 280 °C, respectively. The diterpene products were provisionally identified by comparing them with the NIST mass spectral library and reported characteristic fragment ions.

#### Liquid chromatography-mass spectrometry analysis

For LC–MS analysis, dried extracts were reconstituted in 100 µL of methanol and centrifuged at 13,000 rpm for 10 min. Analyses were performed using a Shimadzu LCMS–8040 triple quadrupole mass spectrometer coupled to an LC system equipped with an SPD-M20A photodiode array detector. Separation was achieved on a Kinetex C18 column (100 × 2.1 mm, 2.6 µm, 100 Å; Phenomenex) at 30 °C. Mobile phase A was water containing 0.1% (v/v) formic acid, and mobile phase B was acetonitrile containing 0.1% (v/v) formic acid. The flow rate was 0.30 mL/min, and the injection volume was 15 µL. The gradient was: 0.01 min, 20% B; 11.00 min, 80% B; 21.00 min, 90% B; 22.00–27.00 min, 100% B; and 28.00–30.00 min, 20% B. PDA spectra were recorded from 190 to 600 nm, with chromatograms monitored primarily at 190 nm. Mass spectra were acquired in positive and negative electrospray ionization modes over *m/z* 100–1500. The nebulizing and drying gas flow rates were 3 and 15 L/min, respectively, and the heat block and desolvation line temperatures were 400 °C and 250 °C, respectively. Selected ion monitoring was used when required, with target ions adjusted according to the analytes.

#### Ultra-fast liquid chromatography analysis and quantification of product 10

UFLC analyses were conducted using a Shimadzu Prominence system (Shimadzu, Japan). The separation was performed on a Unimate^®^ AQ-C18 column (3.0 × 150 mm, 3 µm), with a flow rate of 0.50 mL/min. The injection volume was 15 µL. The mobile phases used were the same as those in the LC–MS analysis. The gradient program was: 0.01 min, 50% B; 18.00 min, 95% B; 19.00 min, 100% B; 29.00 min, 100% B; 30.00 min, 50% B; 38.00 min, 50% B, with a total run time of 39.0 min. PDA spectra were acquired from 190–400 nm.

Product 10 was quantified using an authentic isocupressic acid standard purchased from BioBioPha (China; product no. BBP00361; purity, 96%). Standard solutions at 10, 15, 30, 35, and 45 μg/mL were prepared in methanol to establish an external calibration curve. The calibration curve was constructed by plotting the chromatographic peak area at 190 nm against the corresponding isocupressic acid concentration and showed good linearity over the range of 10–45 μg/mL (Y = 101396.90X + 1,780,285.50, R² = 0.9997), where Y represents the peak area and X represents the isocupressic acid concentration (μg/mL, **Supplementary Figure S23**). For quantification, three independent 15-mL induced yeast cultures were harvested by centrifugation. The cell pellet from each culture was extracted with 600 μL of methanol, 150 μL of acetone, and 50 μL of 0.5-mm glass beads. The extracts were evaporated to dryness and reconstituted in 100 μL of methanol. The reconstituted extracts were centrifuged, and the resulting supernatants were analyzed by UFLC. The isocupressic acid concentration in the final extract (Cextract, μg/mL) was calculated using the external calibration curve. The fermentation titer was calculated according to the following equation: titer (μg/L) = Cextract (μg/mL) × 0.100 mL/0.015 L. Results are presented as mean ± SD from three independent biological replicates (**Supplementary Table S12**).

#### Liquid chromatography–high-resolution mass spectrometry analysis and data acquisition

LC–HRMS analyses were performed using an Agilent 1290 Infinity II LC system coupled with an Agilent 6545 Q-TOF mass spectrometer. Chromatographic separation was achieved using the same Kinetex C18 column (100 × 2.1 mm, 2.6 µm, 100 Å; Phenomenex). The mobile phases were the same as mentioned above. The flow rate was 0.30 mL/min. The gradient program was as follows: 0.00 min, 20% B; 11.00 min, 80% B; 21.00–27.00 min, 100% B; and 35.00 min, 20% B. The injection volume was 1.0 µL. Mass spectra were acquired in full-scan mode over *m/z* 100–1000.

### Metabolite purification by semi-preparative HPLC

Large-scale fermentation was performed under the same cultivation conditions described above. The yeast cells were harvested, and the metabolites were extracted from the cell pellets, as described in the Metabolite Extraction section. The combined crude extract was concentrated and subjected to semi-preparative HPLC using an Agilent 1260 Infinity II system equipped with a YMC-Pack ODS-A column (250 × 10.0 mm, 5 µm, 12 nm). The mobile phases were the same as those used for the UFLC analysis of product 10, but the flow rate was increased to 3.0 mL/min. The gradient program was as follows: 0.01 min, 50% B; 18.00 min, 95% B; 19.00 min, 100% B; 29.00 min, 100% B; 30.00 min, 50% B; and 38.00 min, 50% B, for a total run time of 39.0 min. PDA spectra were acquired from 190–400 nm. The injection volume was 100 µL. Fractions corresponding to the target peaks were collected, concentrated, and verified by analytical LC–MS/PDA before NMR analysis.

### Structure characterization by NMR spectroscopy

Purified compounds were dissolved in CDCl_3_ and analyzed using a Bruker Avance III 600 MHz NMR spectrometer. ¹H and ¹³C NMR and DEPT-135 spectra, together with two-dimensional COSY, HSQC, HMBC, and NOESY spectra, were acquired using standard pulse sequences. Chemical shifts were referenced to the residual solvent signals of CDCl_3_. Spectra were processed using MestReNova software. Structural assignments were based on the combined one- and two-dimensional NMR data and comparison with authentic standards or published data.

### Absolute quantitative RT–qPCR

Fresh leaf, stem, and sarcotesta tissues were collected on 11 August 2026 from three mature *G. biloba* individuals grown in the Baicao Garden, Shanghai, China. Samples from the same plant were treated as matched samples, with each plant representing one biological replicate (n = 3).

Total RNA was extracted using a SteadyPure Plant RNA Extraction Kit (AG21019; Accurate Biology). RNA concentration and purity were assessed using a DS-11+ Spectrophotometer, and integrity was evaluated by 1% agarose gel electrophoresis. Genomic DNA removal and reverse transcription were performed in 20 μL using an Evo M-MLV RT Mix Kit with gDNA Clean for qPCR Ver. 2 (AG11728; Accurate Biology). For Gb_04669, 1 μg of total RNA was used for each leaf sample, whereas 14 μL of RNA solution was used for each sarcotesta and stem sample, with the actual RNA input accounted for in subsequent calculations. For Gb_33836, 300 ng of total RNA was used for each sample. Reverse transcription was performed at 37 °C for 15 min and 85 °C for 5 s, and the cDNA was used without dilution.

Quantitative PCR was performed using a QuantStudio 6 Flex system and SYBR Green Pro Taq HS Premix with ROX (AG11735; Accurate Biology). Each 20-μL reaction contained 10 μL of 2× premix, 0.5 μL of 40× Dilution Buffer, 0.4 μL each of 10 μM forward and reverse primers, and 2 μL of cDNA for Gb_04669 or 1 μL for Gb_33836. Amplification comprised 95 °C for 30 s, followed by 40 cycles of 95 °C for 5 s and 60 °C for 30 s. Three technical reactions were performed per biological sample, and their Ct values were averaged.

Absolute quantification used plasmid standards containing the target sequences. The Gb_04669 and Gb_33836 standard plasmids were 4,916 and 4,014 bp, respectively (**Supplementary Table S6**). Tenfold serial dilutions were prepared from 3 ng μL⁻¹ stocks, and copy numbers were calculated as concentration (g μL⁻¹) × 6.022 × 10²³/[plasmid length (bp) × 660]. Standard curves were generated by plotting Ct against log_10_ copies per microliter (**Supplementary Figure S22**). Mean Ct values were converted using the corresponding regression equation and corrected for qPCR template volume, reverse-transcription volume, and RNA input. Results are reported as qPCR-derived target copies per microgram of total RNA.

## Data Availability

The datasets presented in this study can be found in online repositories. The names of the repository/repositories and accession number(s) can be found in the article/**Supplementary Material**.
