# Supplementary material for: Functional identification of two cytochrome P450 enzymes involved in diterpenoid oxidation in Ginkgo biloba
Source: Front Plant Sci. 2026 Sep 16;17:1943006. doi: 10.3389/fpls.2026.1943006 (PMC13623857; doi:10.3389/fpls.2026.1943006)
Supplement: Supplementary file 1 [file SupplementaryFile1.pdf]

Supplementary Material

1. Supplementary Figures

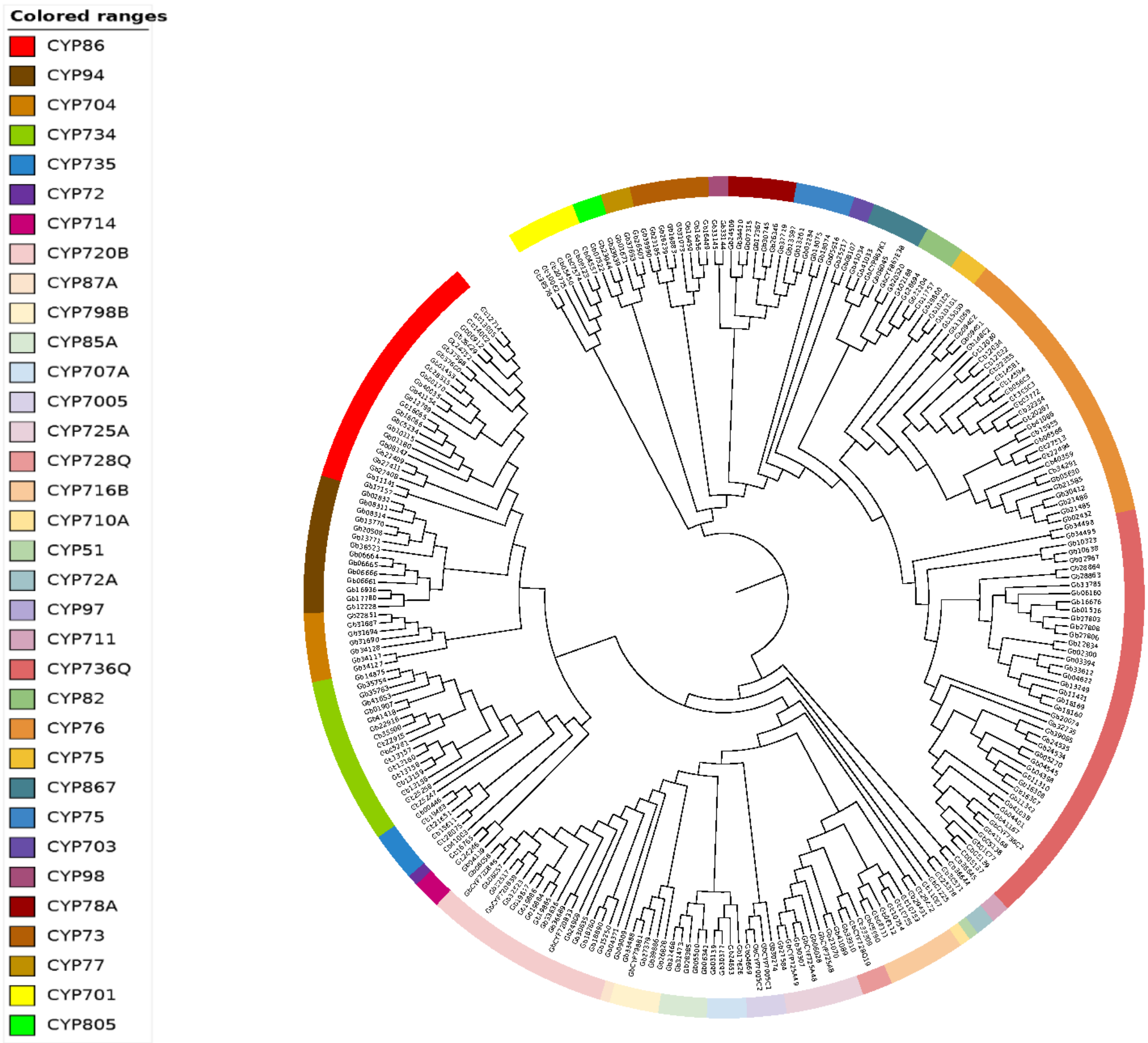

Figure S1. Phylogenetic analysis of cytochrome P450s from *Ginkgo biloba*. A phylogenetic tree was constructed using the analysed amino acid sequences of the cytochrome P450 proteins from *Ginkgo biloba*.

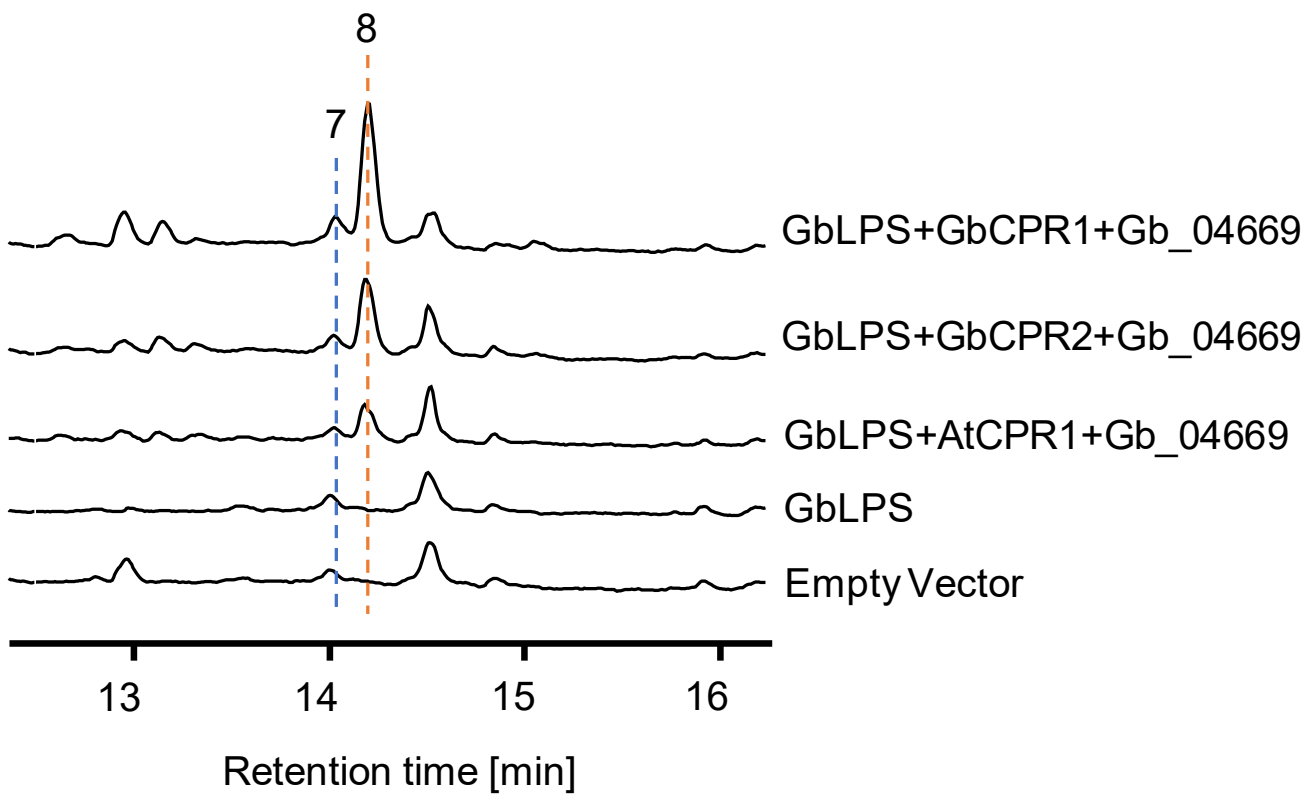

Figure S2. A comparison of product formation facilitated by various cytochrome P450 reductases (CPRs). Representative gas chromatography–mass spectrometry (GC–MS) chromatograms show extracts from strains that co-express GbLPS, Gb_04669, and various CPRs, specifically GbCPR1, GbCPR2, and AtCPR1. These are compared to strains expressing only GbLPS and empty vector strains. Two product-related peaks, labelled 6 and 8, are highlighted. The dashed lines indicate the retention times of peaks 7 and 8, demonstrating the differences in product accumulation among the CPR partners.

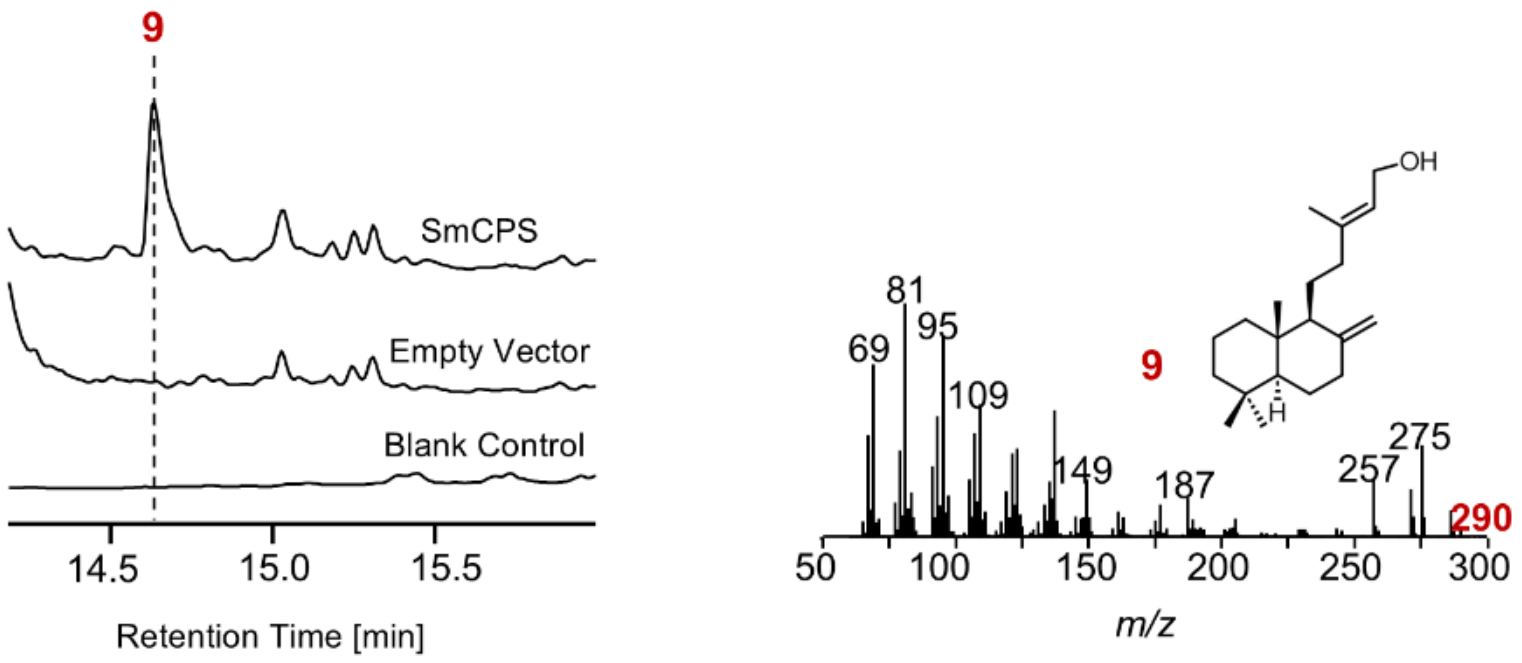

Figure S3. GC–MS analysis of peak 9 detected in the SmCPS expressing yeast strain. (A) Representative GC–EIC chromatograms of extracts from yeast strains expressing SmCPS, the corresponding empty vector control, and the blank control. Peak 9 was specifically detected in the SmCPS expressing strain, whereas no corresponding peak was observed in the empty vector or blank control. (B) EI mass spectrum of peak 9 acquired by GC–MS. The spectrum displayed a molecular ion at m/z 290 together with major fragment ions at m/z 275, 257, 187, 149, 109, 95, 81, and 69. Peak 9 was assigned as (+)-copalol by comparison with the NIST17 mass spectral database and the previously reported mass spectrum described by from Ke Gao *et al* (Gao et al., 2024).

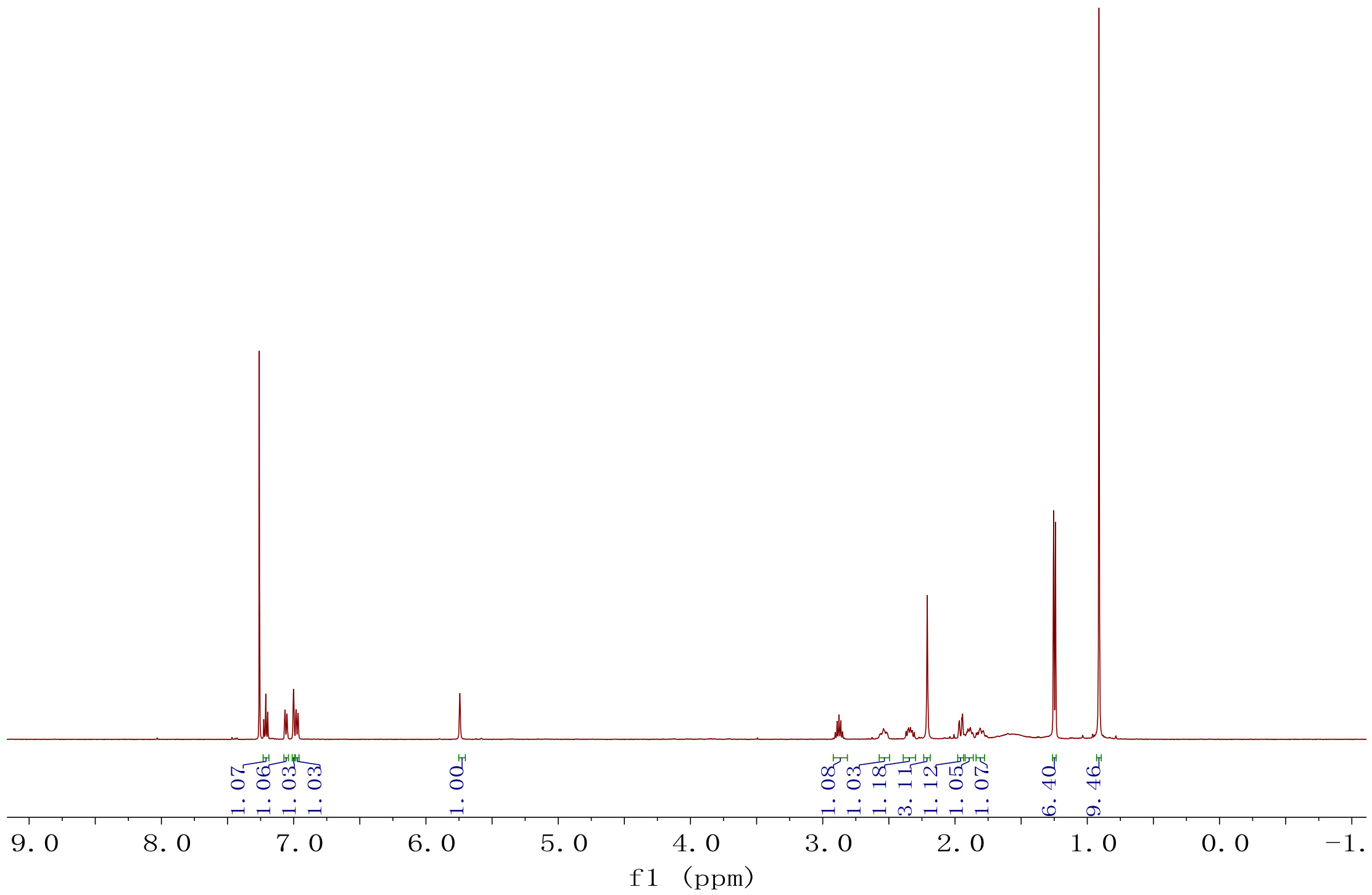

Figure S4. ^{1}H NMR spectrum (600 MHz) of compound 7 in chloroform-d.

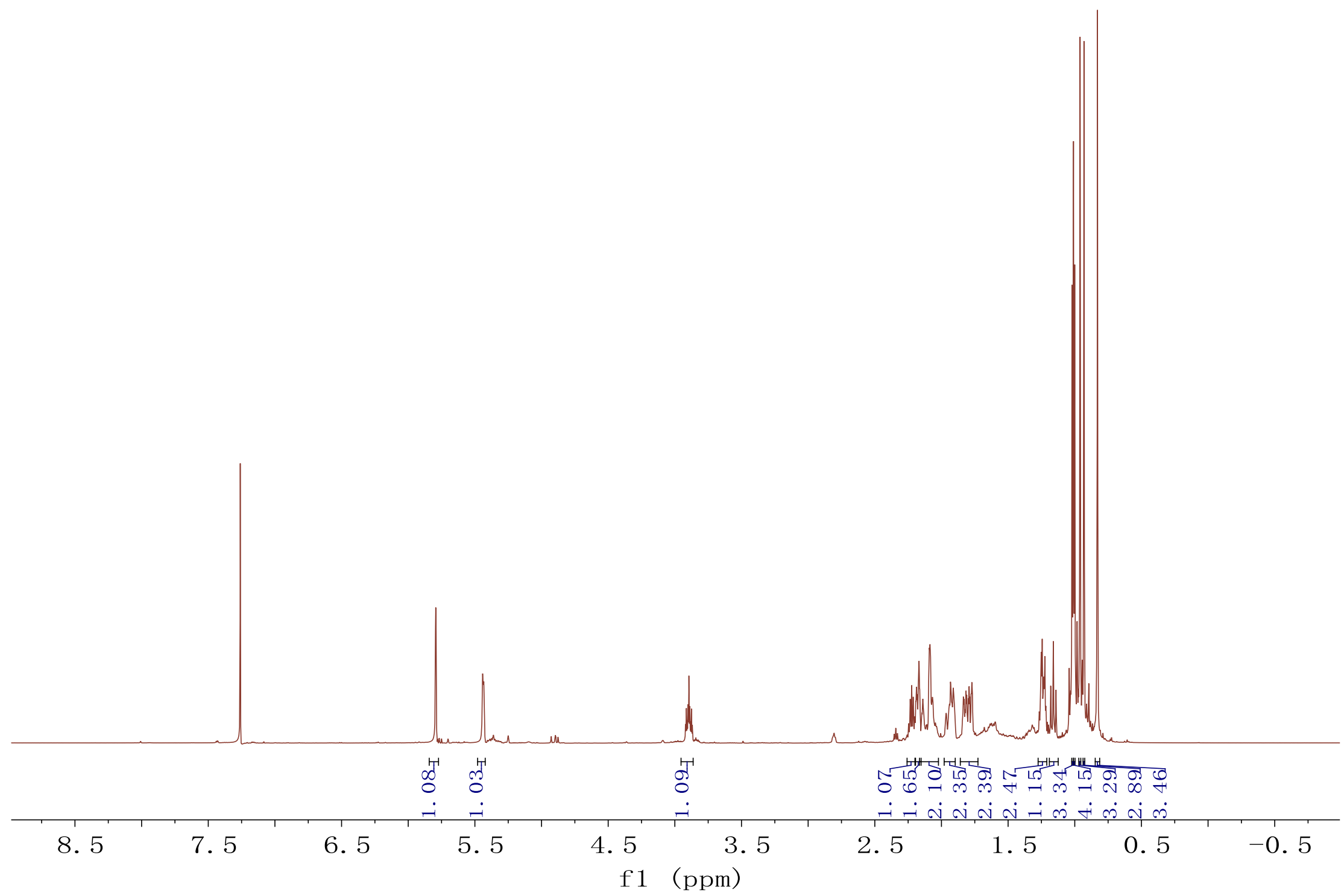

Figure S5. ^{1}H NMR spectrum (600 MHz) of compound 8 in chloroform-d.

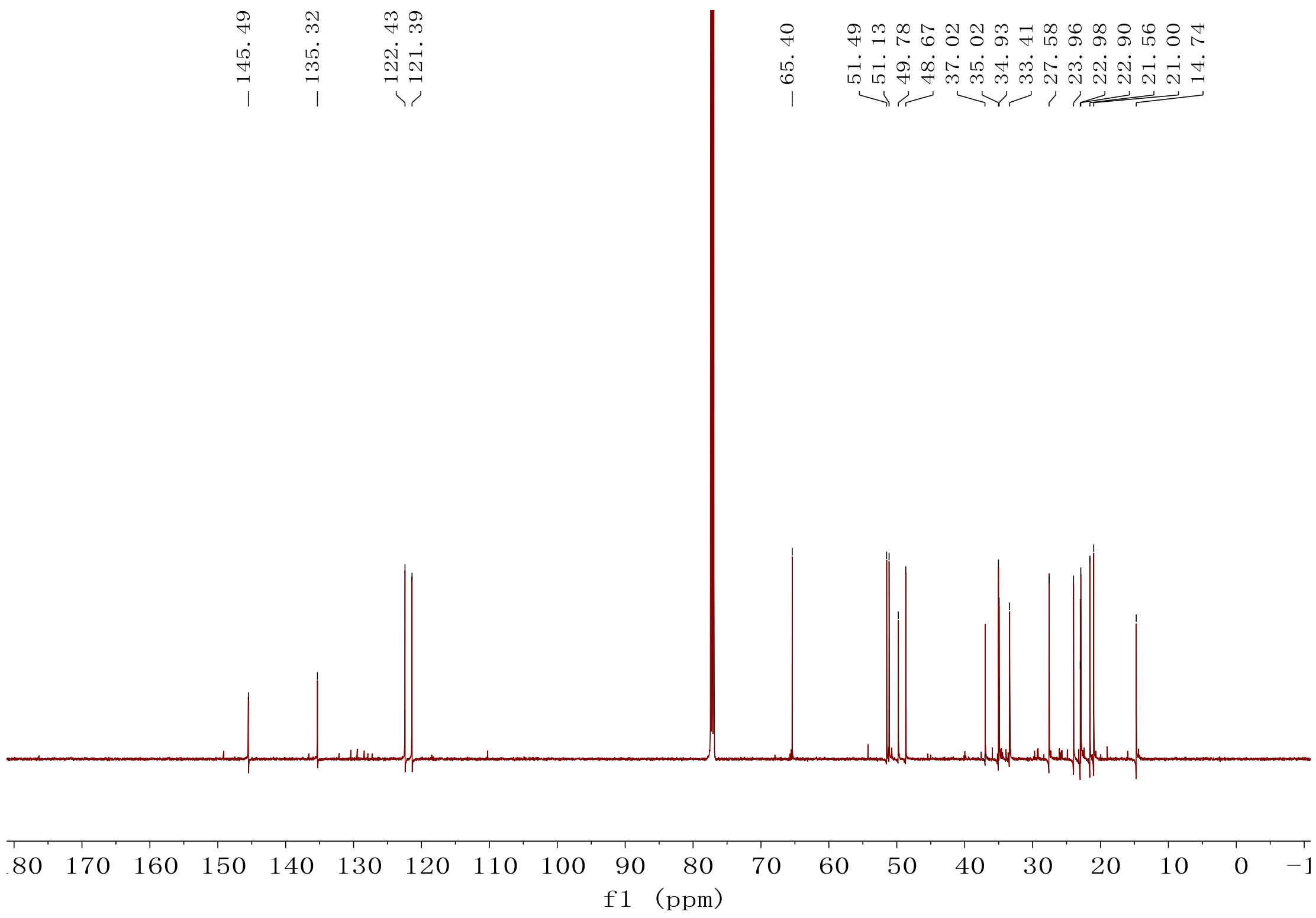

Figure S6. ^{13}C NMR spectrum (151 MHz) of compound 8 in chloroform-d.

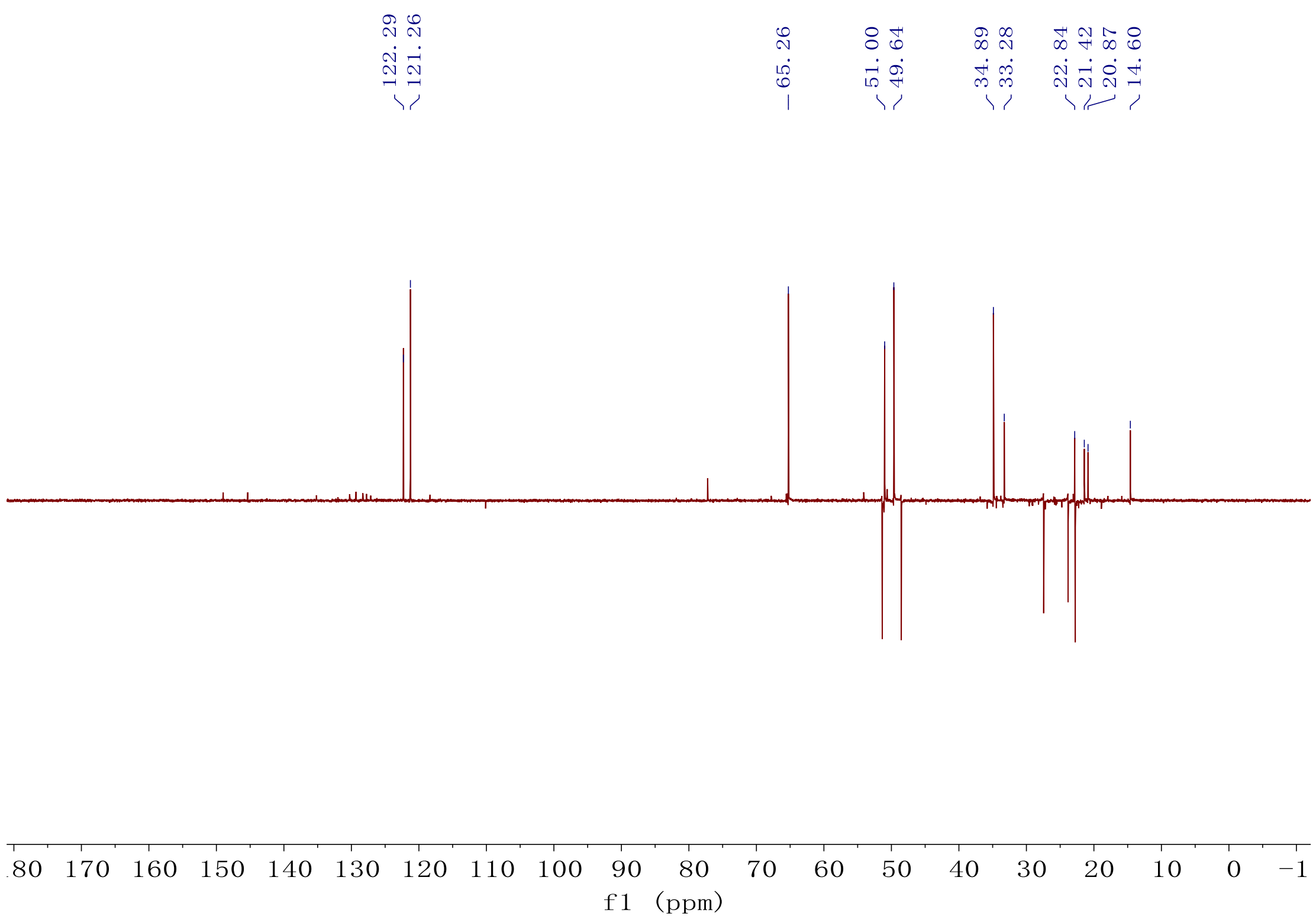

Figure S7. DEPT135 NMR spectrum of compound 8 in chloroform-d.

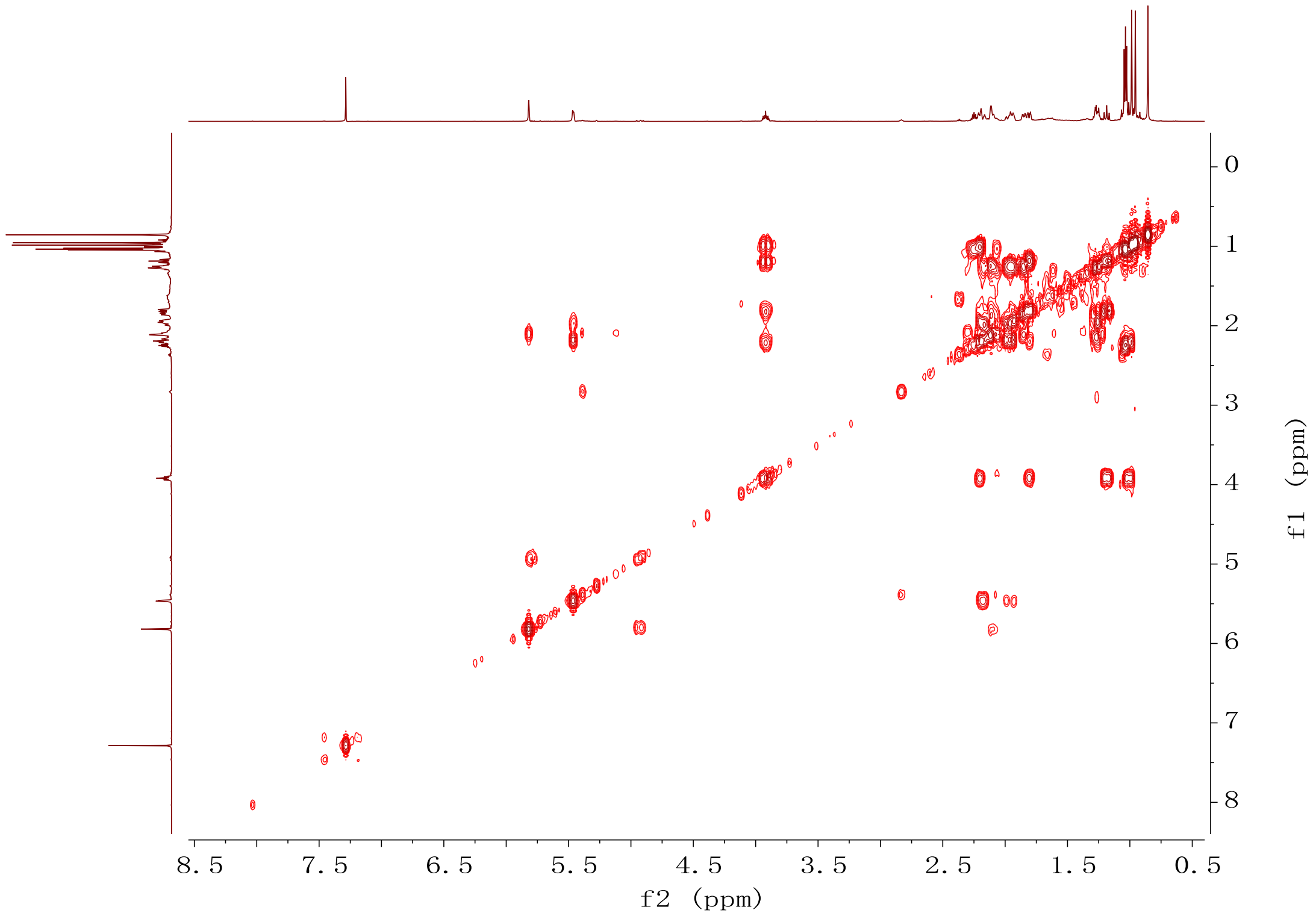

Figure S8. H-H COSY NMR spectrum of compound 8 in chloroform-d.

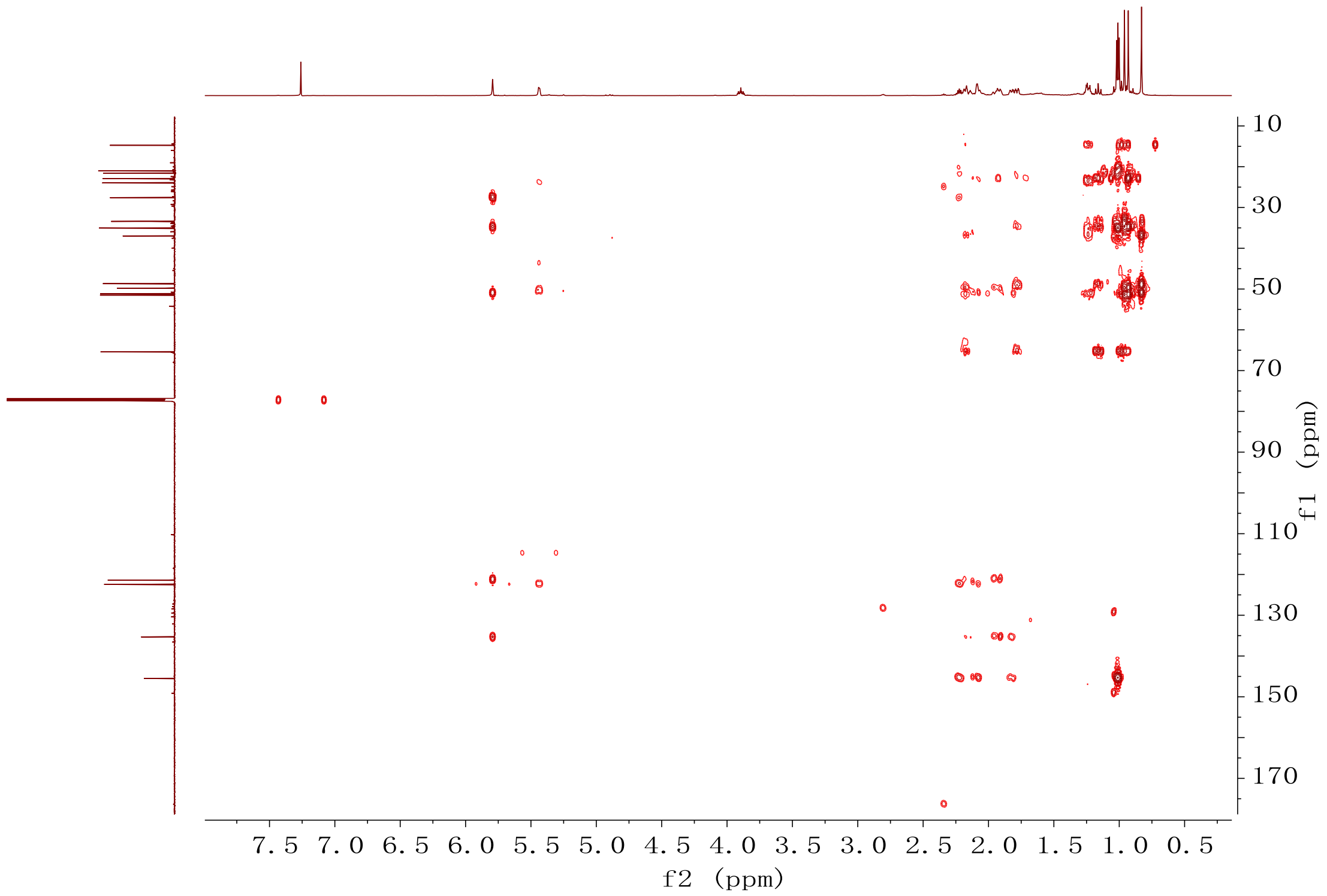

Figure S9. HMBC NMR spectrum of compound 8 in chloroform-d.

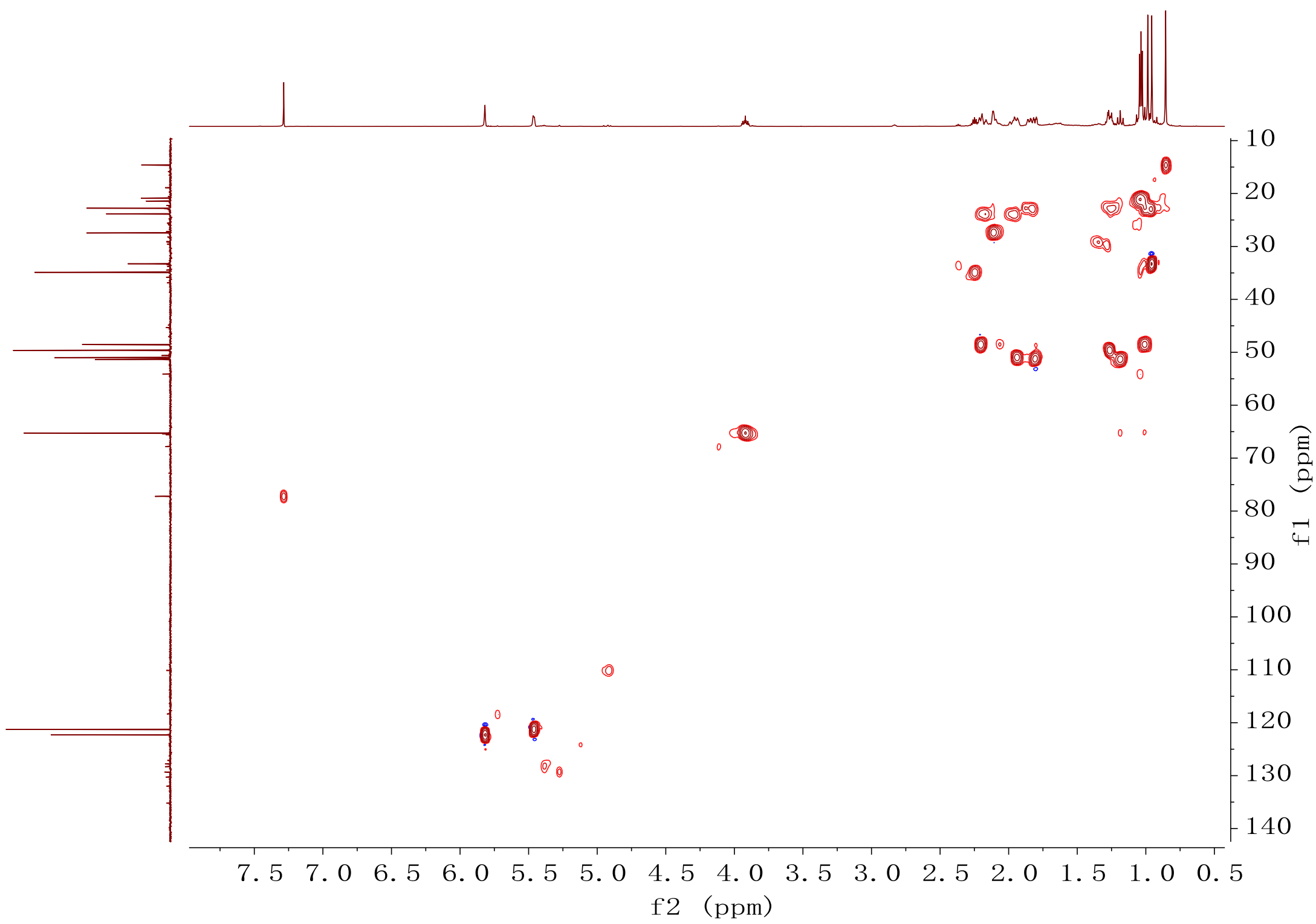

Figure S10. HSQC NMR spectrum of compound 8 in chloroform-d.

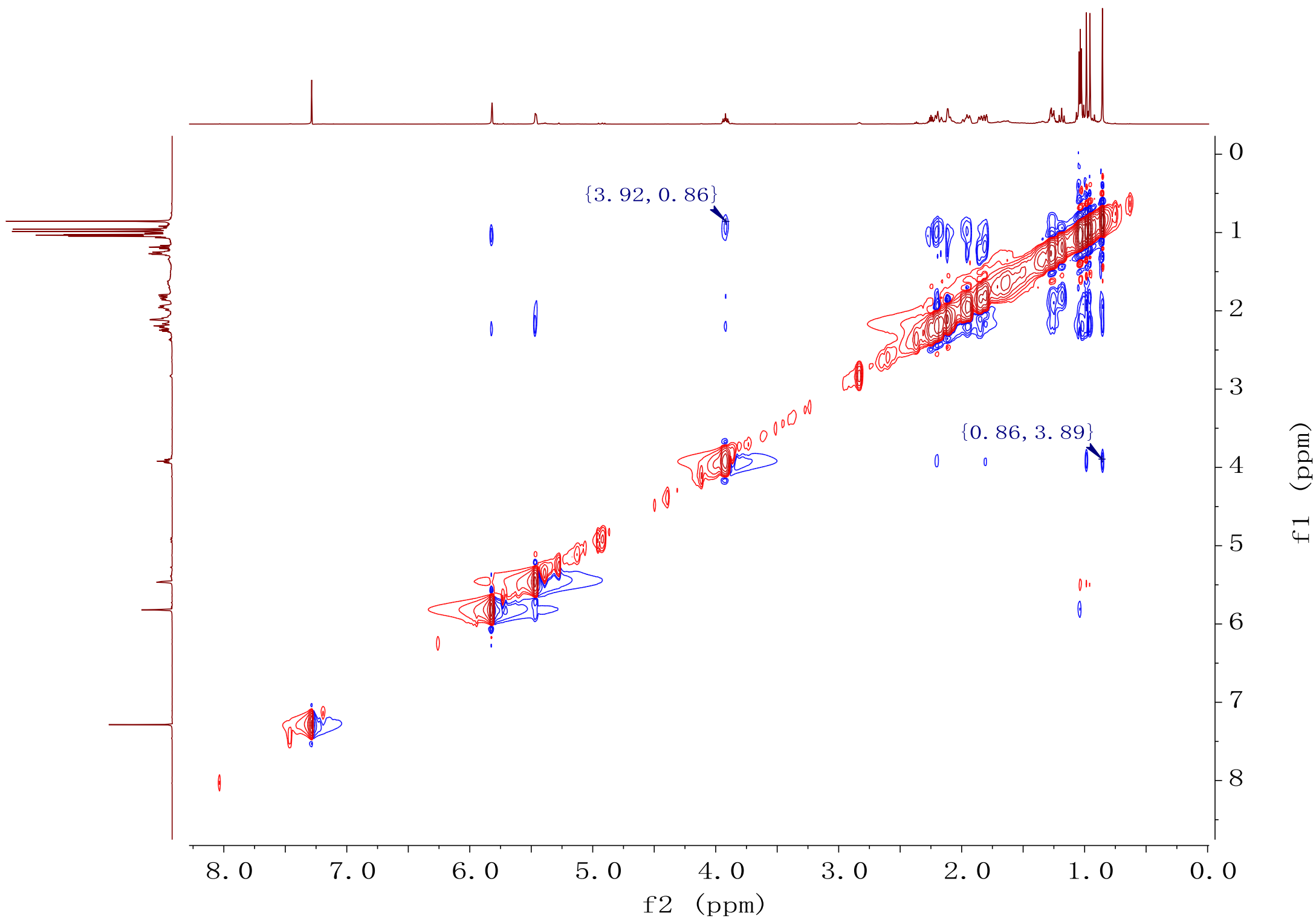

Figure S11. NOESY NMR spectrum of compound 8 in chloroform-d.

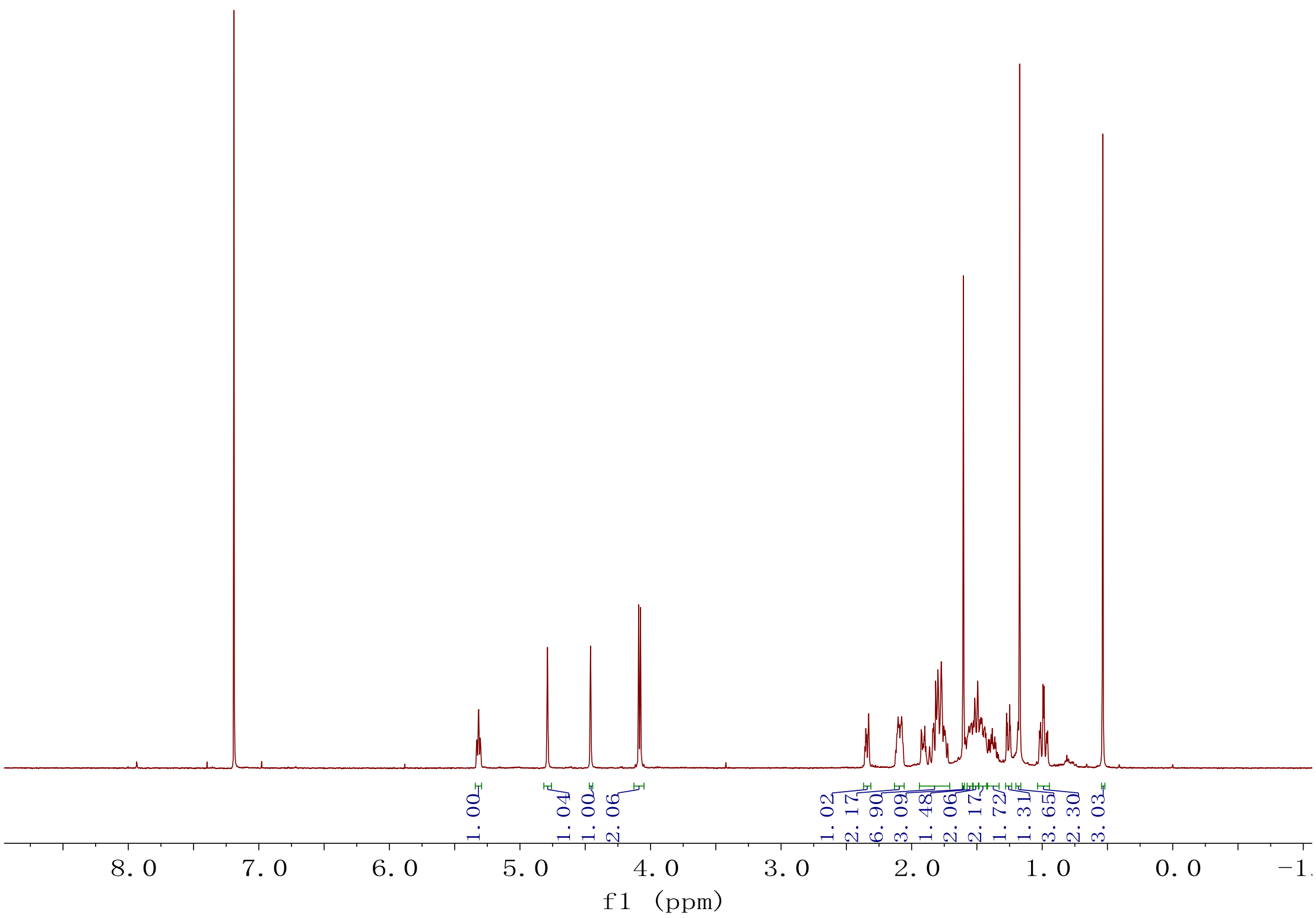

Figure S12. ^{1}H NMR spectrum (600 MHz) of compound 10 in chloroform-d.

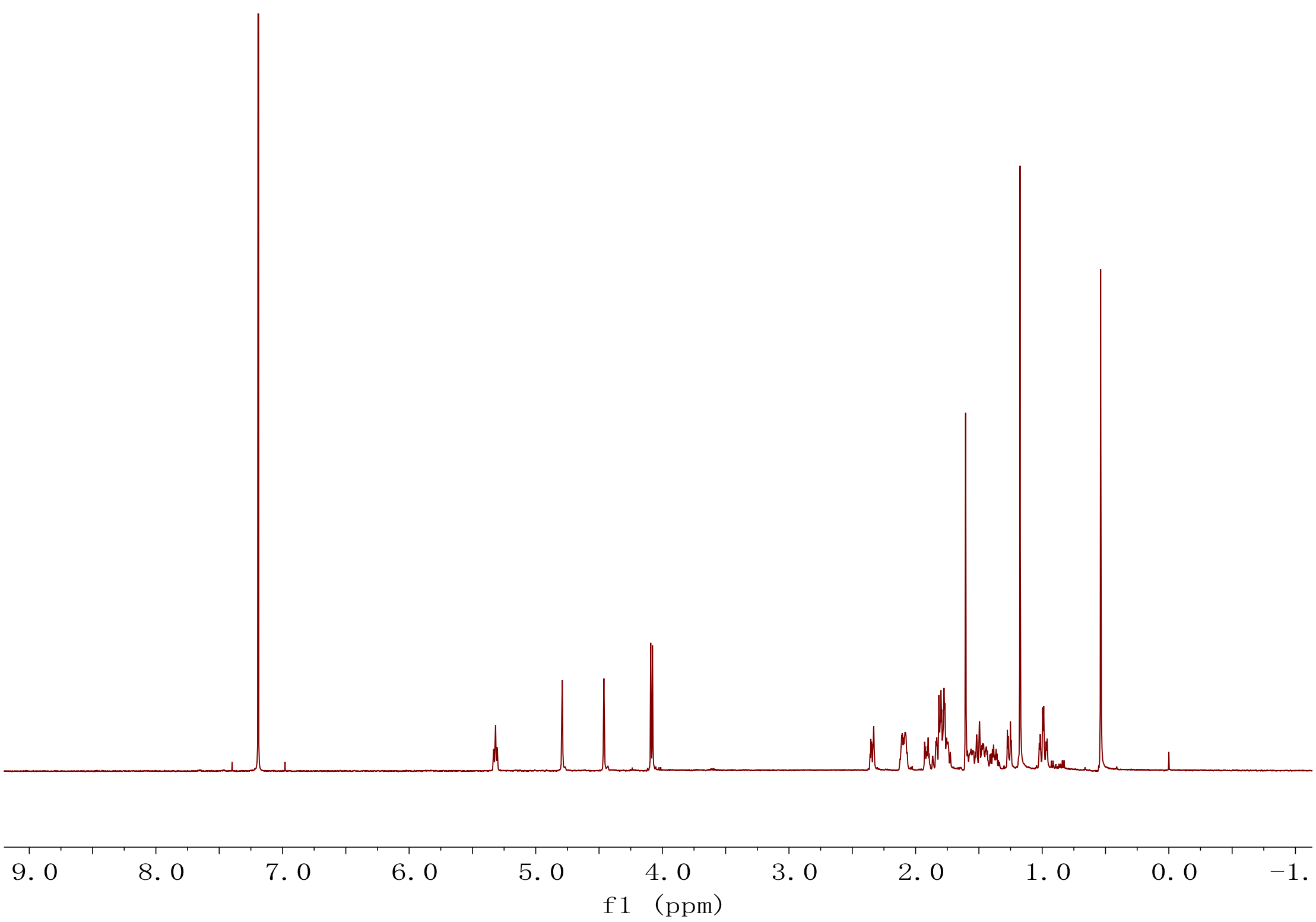

Figure S13. ^{1}H NMR spectrum (600 MHz) of isocupressic acid standard in chloroform-d.

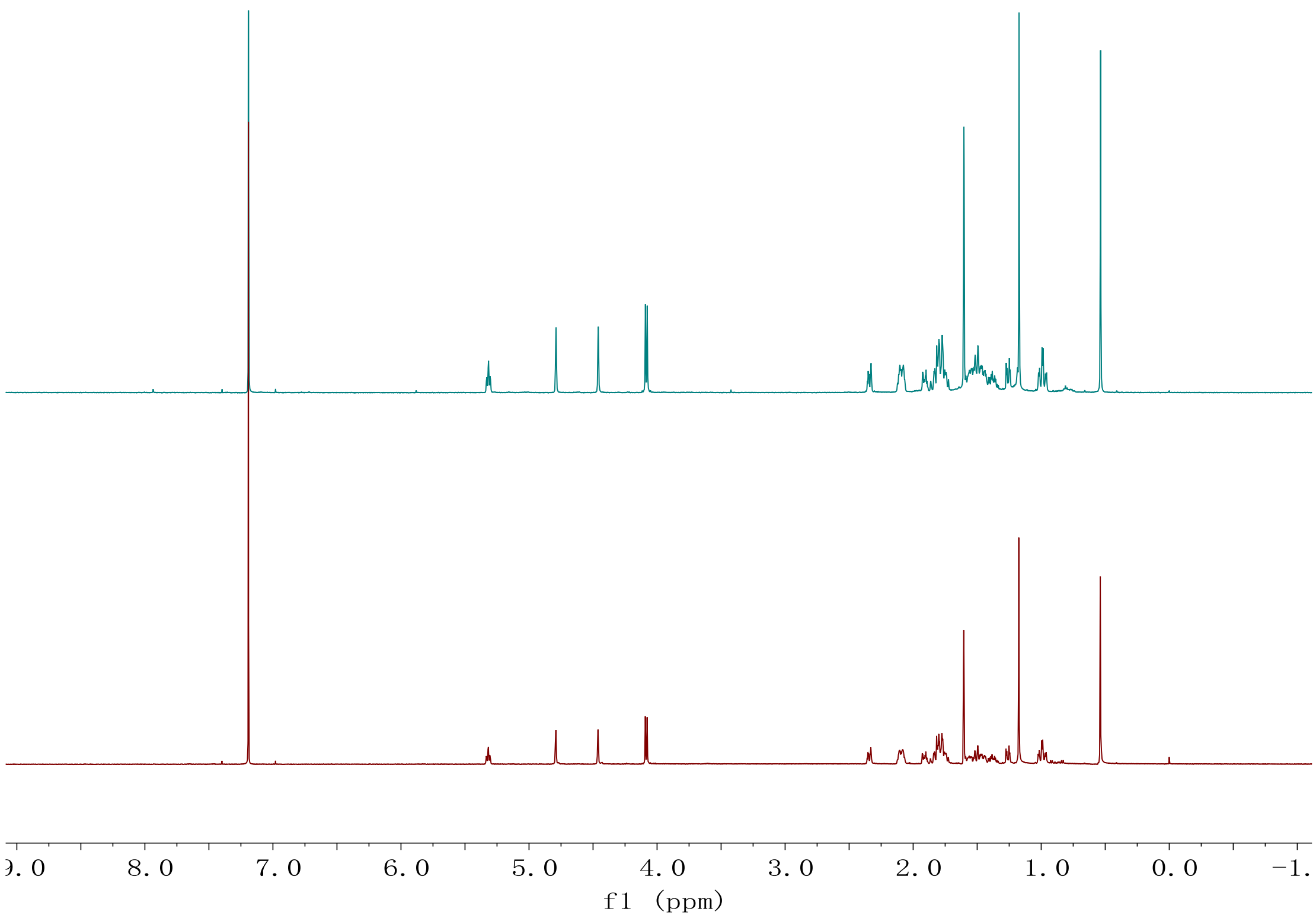

Figure S14. ^{1}H NMR spectrum (600 MHz) of isocupressic acid standard compared with compound 10 in chloroform-d.

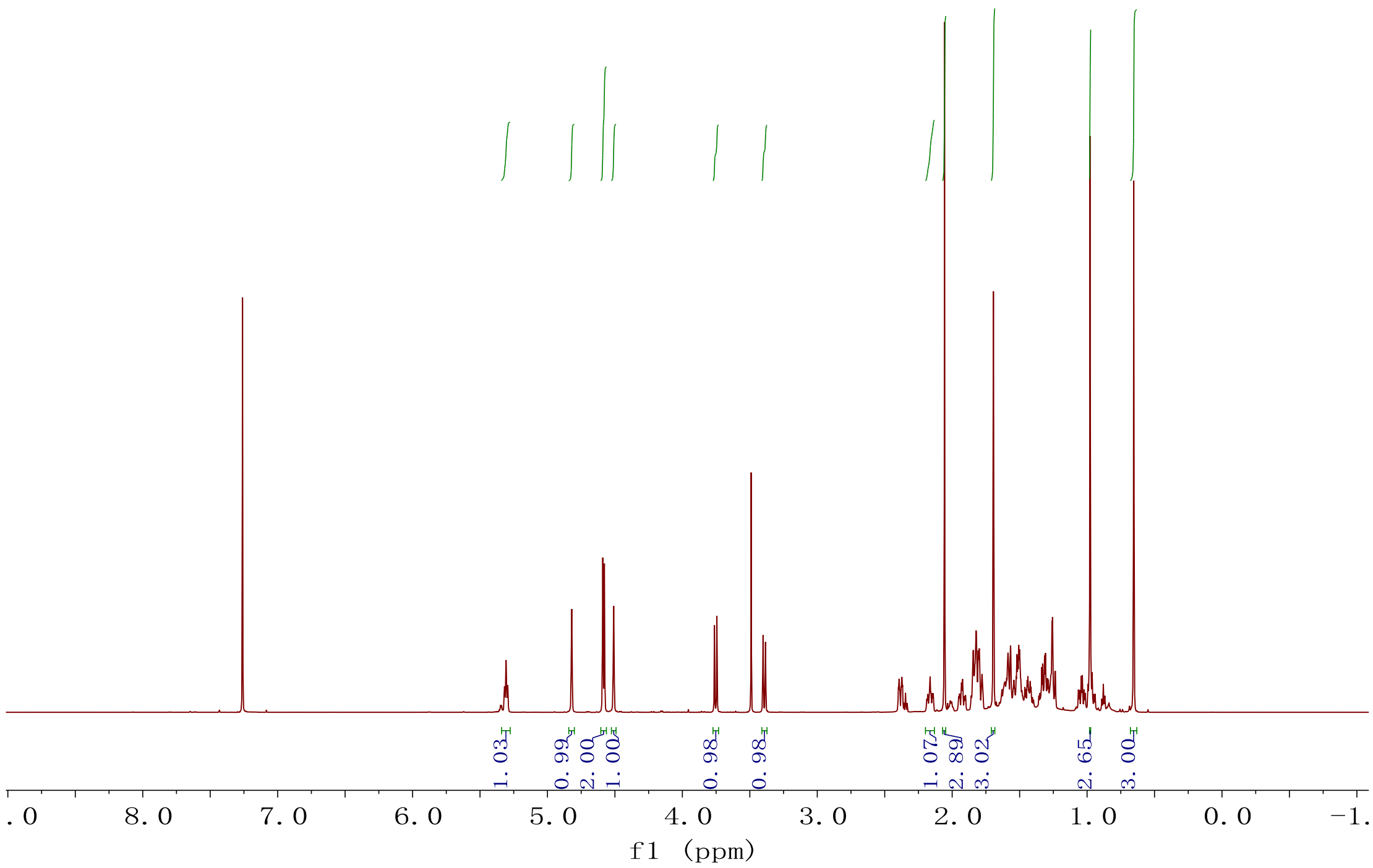

Figure S15. ^{1}H NMR spectrum (600 MHz) of compound 11 in chloroform-d.

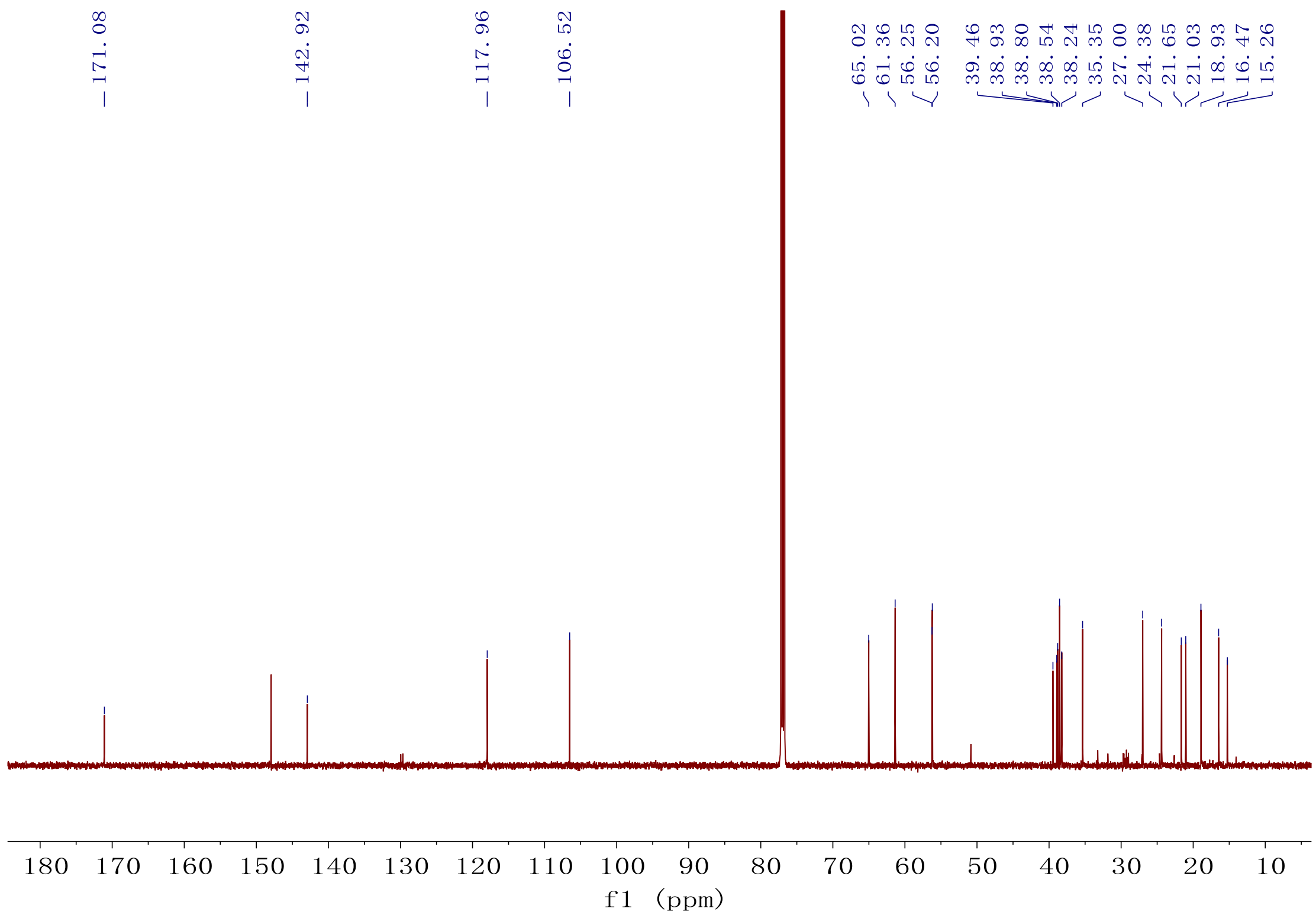

Figure S16. ^{13}C NMR spectrum (151 MHz) of compound 11 in chloroform-d.

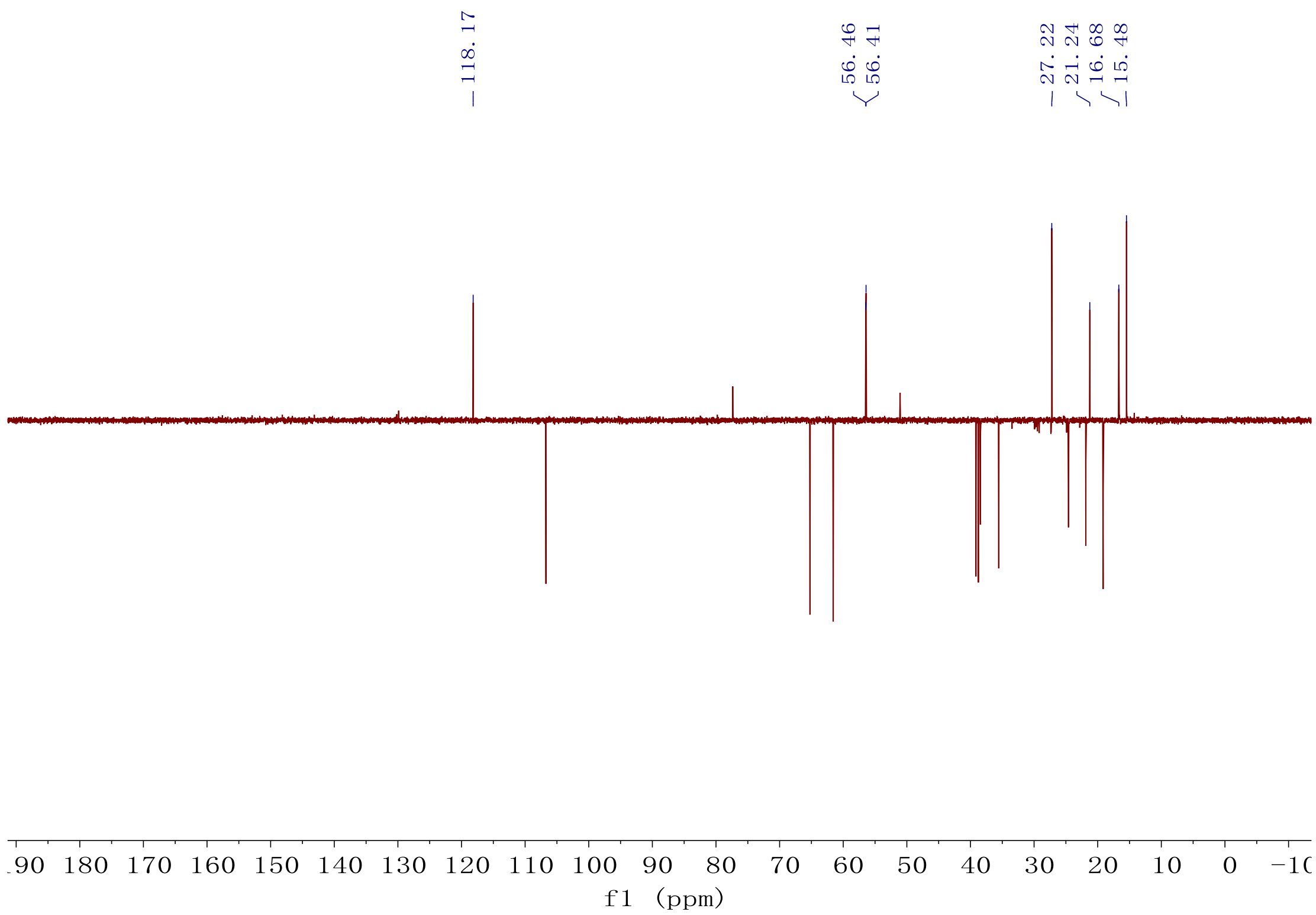

Figure S17. DEPT135 NMR spectrum of compound 11 in chloroform-d.

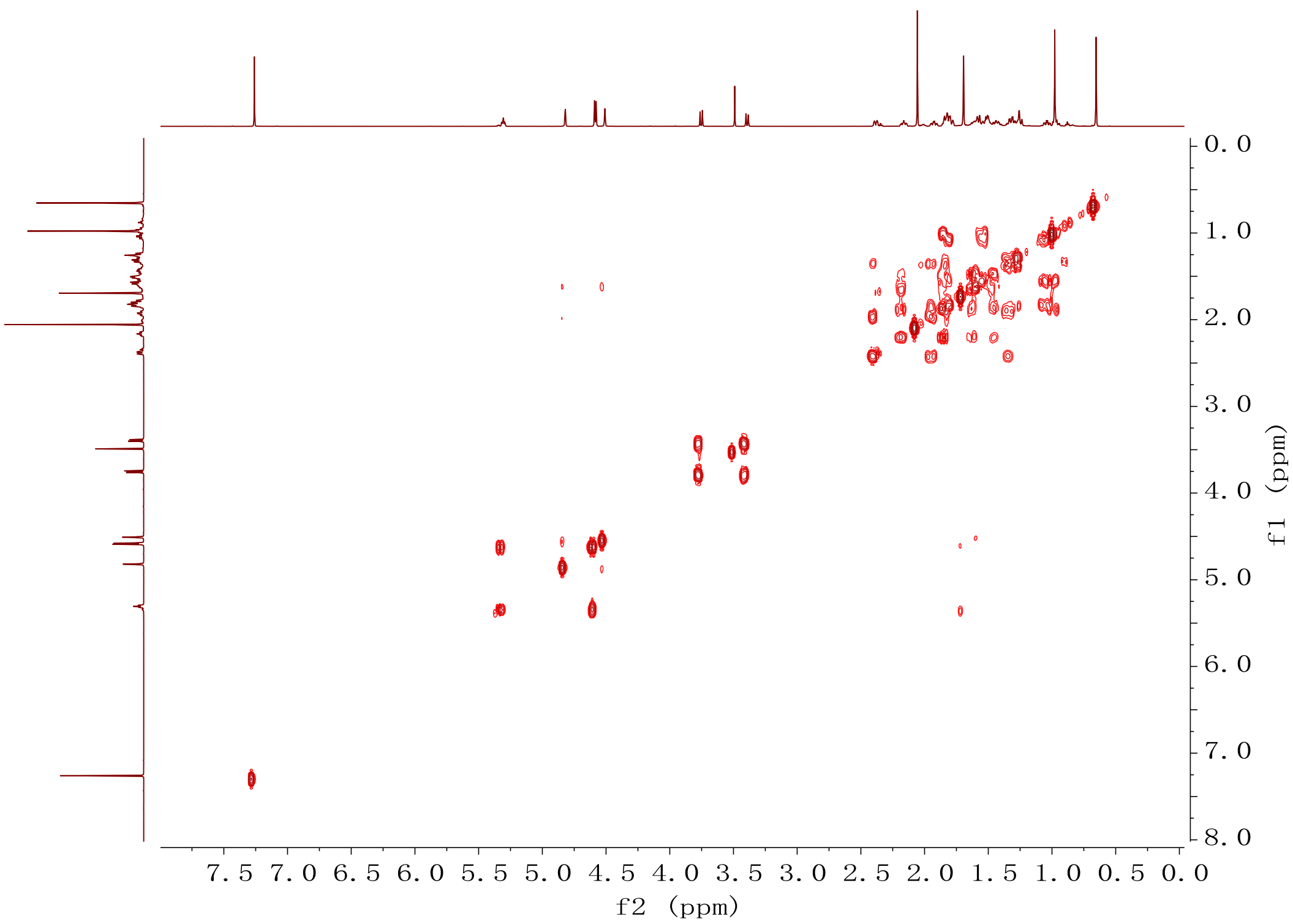

Figure S18. H-H COSY NMR spectrum of compound 11 in chloroform-d.

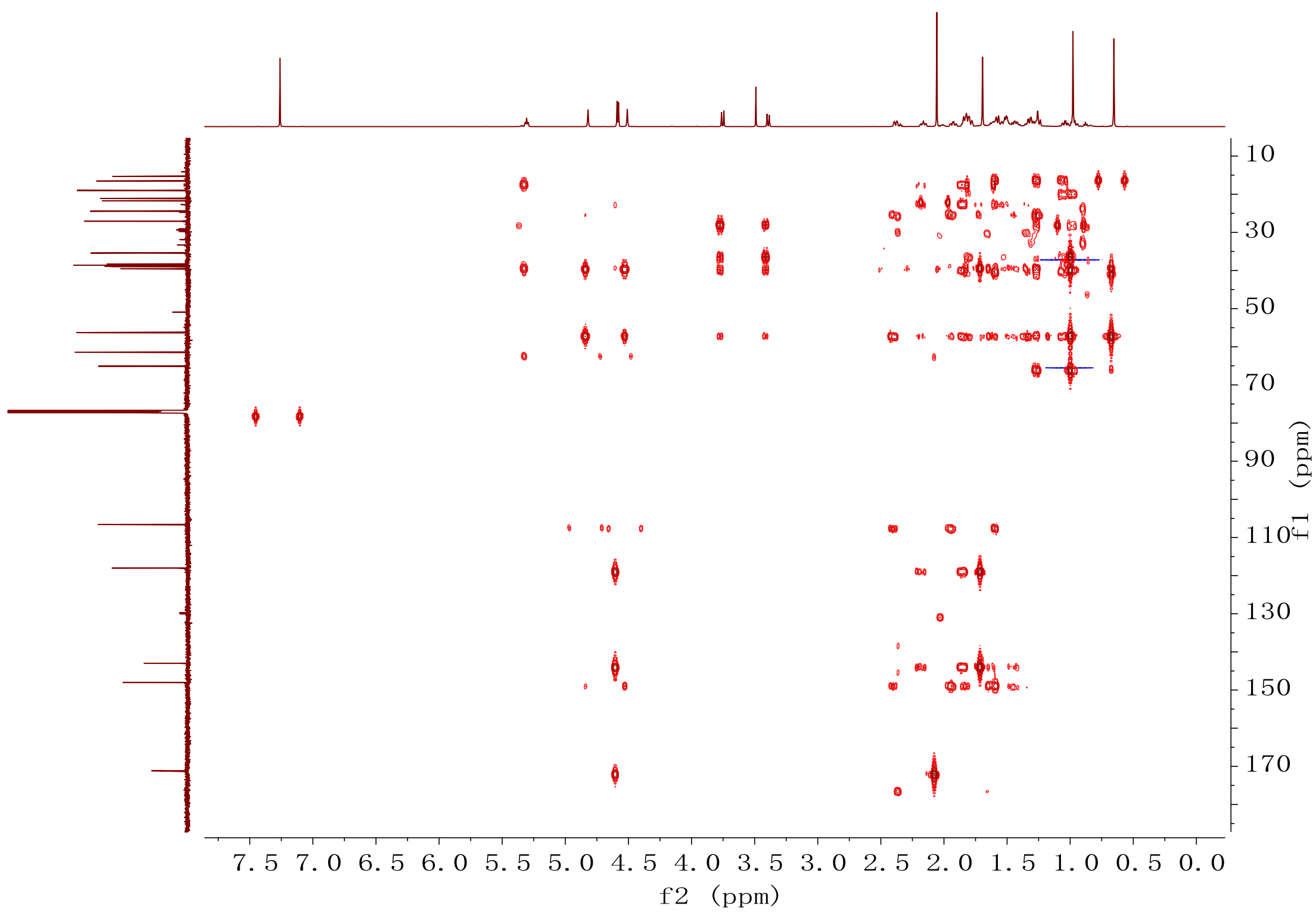

Figure S19. HMBC NMR spectrum of compound 11 in chloroform-d.

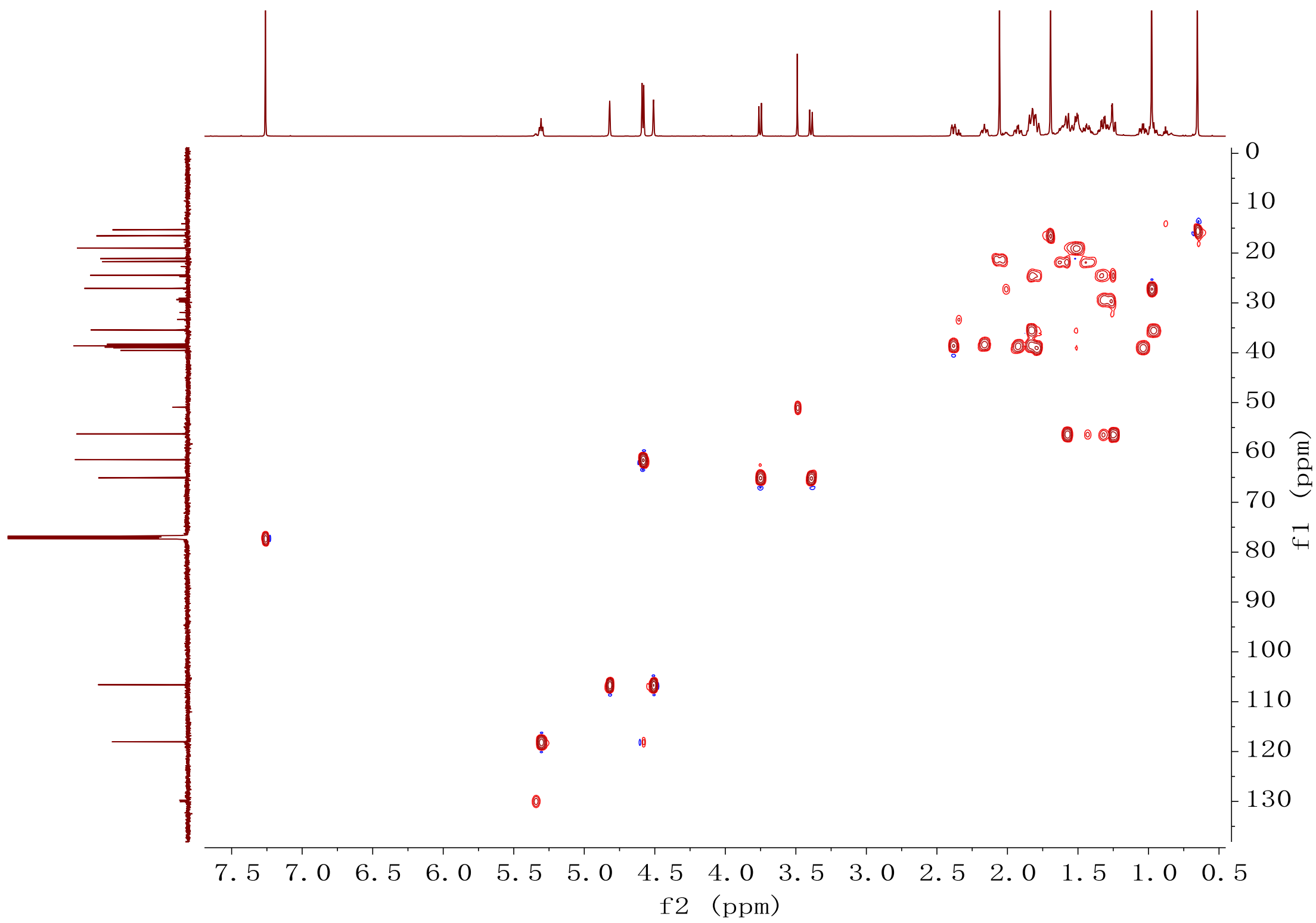

Figure S20. HSQC NMR spectrum of compound 11 in chloroform-d.

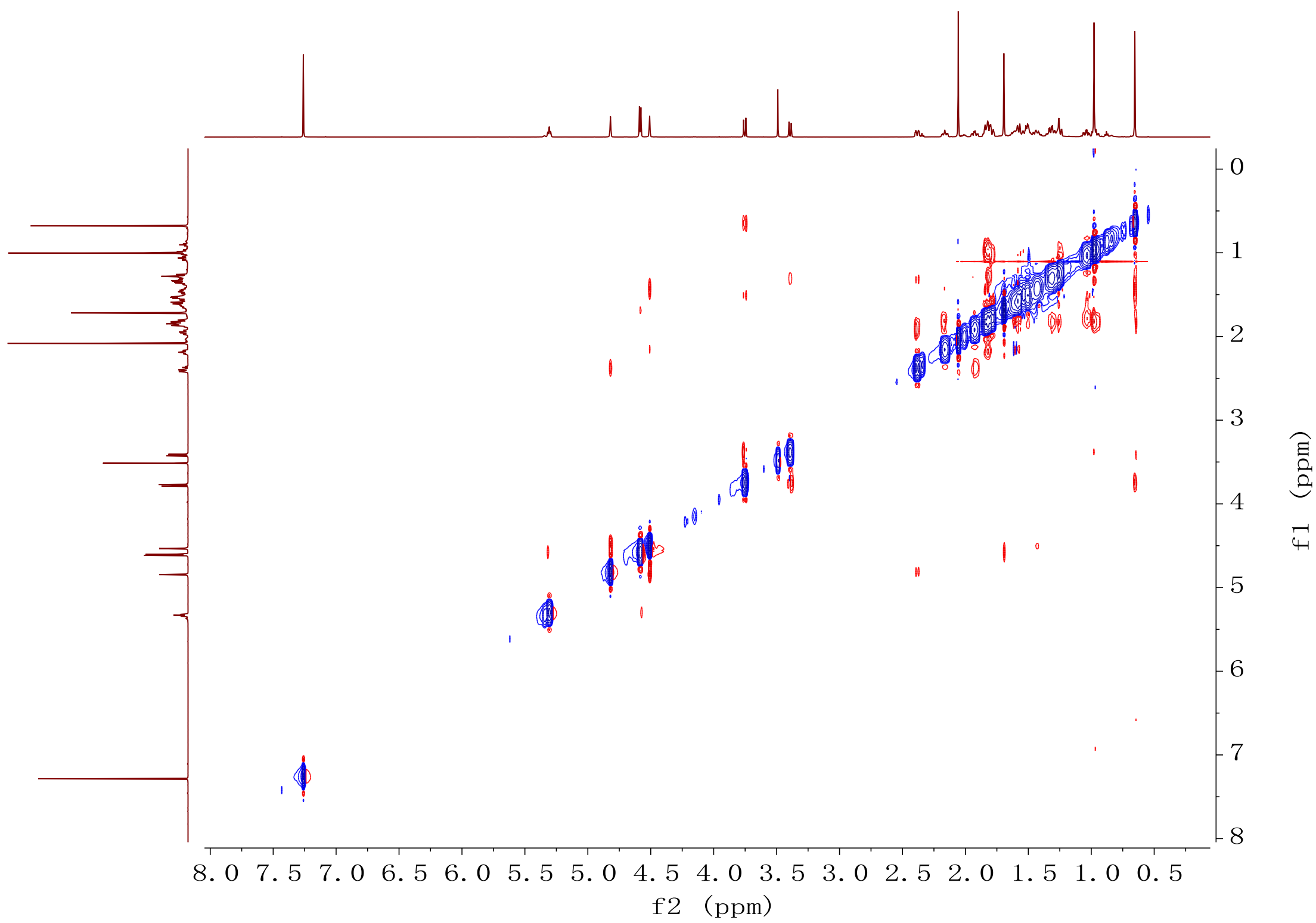

Figure S21. NOESY NMR spectrum of compound 11 in chloroform-d.

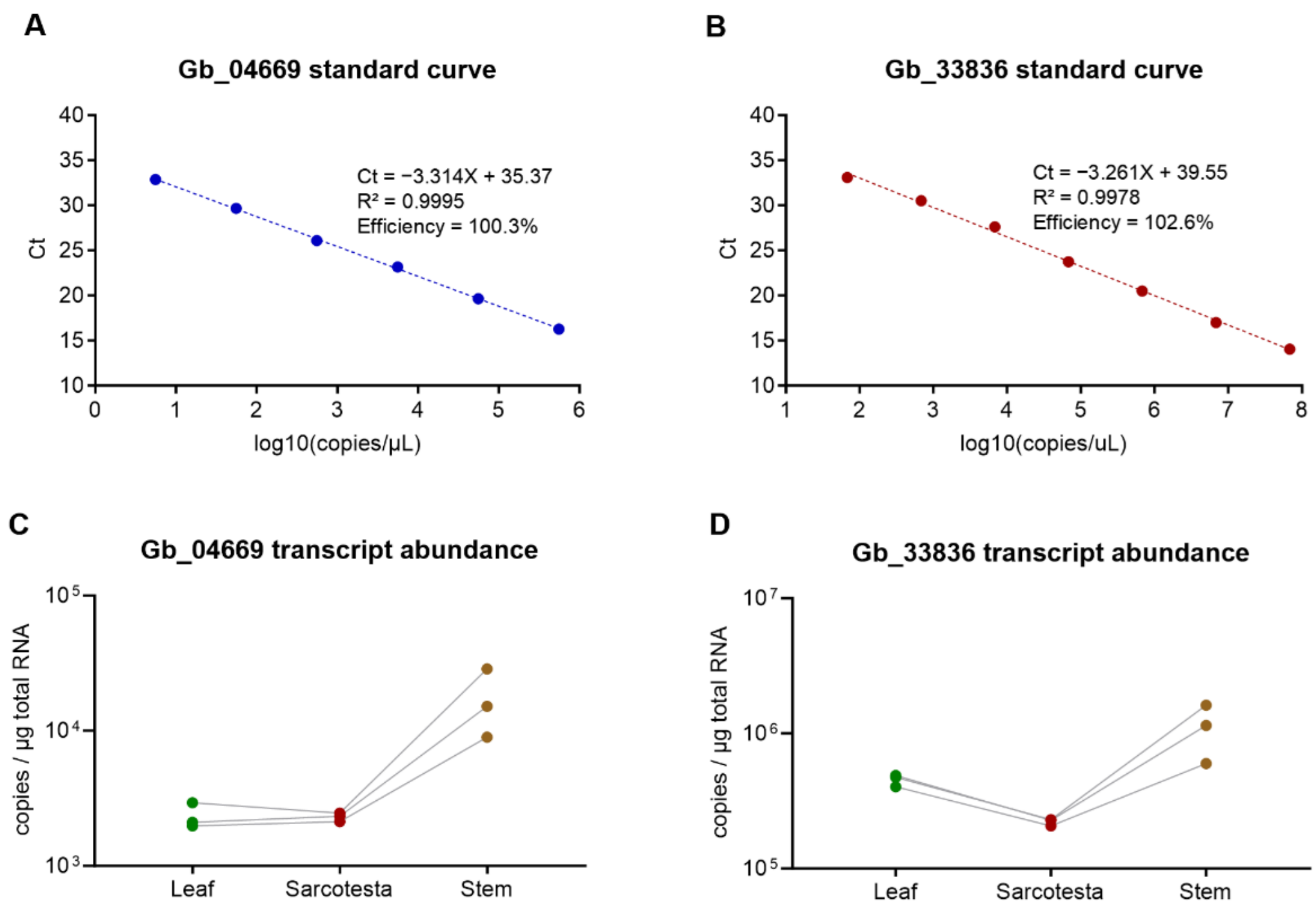

Figure S22. Plasmid standard curves and qPCR-derived transcript copy numbers of Gb_04669 and Gb_33836 in *Ginkgo biloba* tissues. (A, B) Plasmid-based standard curves for Gb_04669 and Gb_33836, respectively. The amplification efficiencies were 100.3% for Gb_04669 and 102.6% for Gb_33836. (C, D) qPCR-derived transcript copy numbers per microgram of total RNA in leaf, sarcotesta, and stem tissues. Each point represents one independent plant (n = 3), and values obtained from the same plant are connected by lines.

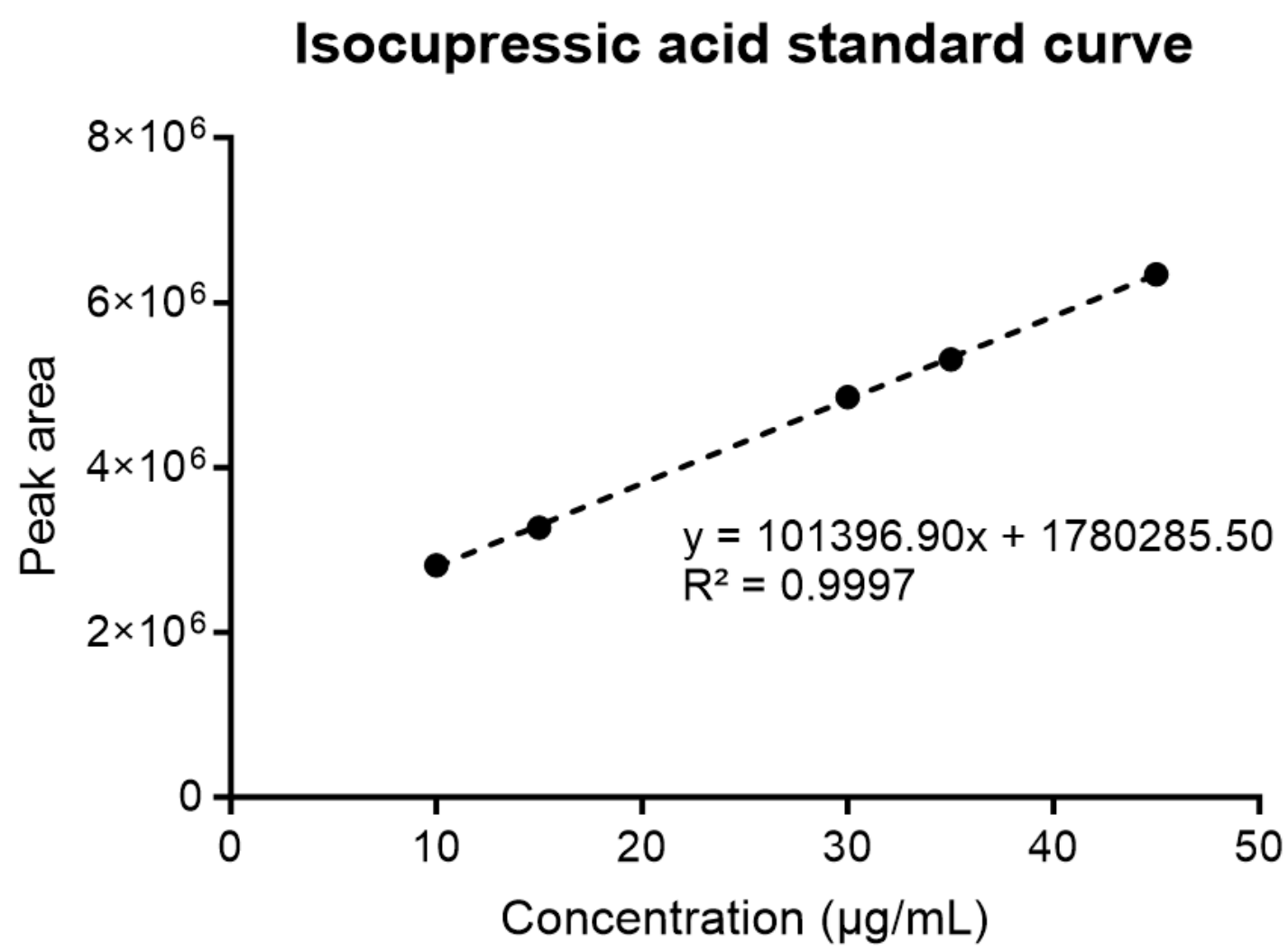

Figure S23. External calibration curve for isocupressic acid. The calibration curve was generated using isocupressic acid standards at 10, 15, 30, 35, and 45 µg mL^{-1}. Peak areas were measured by UFLC–PDA. Linear regression yielded the equation (y = 101396.90x + 1780285.50), with (R^2 = 0.9997). Here, (x) represents the isocupressic acid concentration and (y) represents the chromatographic peak area.

2. Supplementary Tables

Table S1. Strains used in the study.

Yeast Strain	Description	Genotype	Function	Reference
CEN.PK2-1D		*MatALPHA; ura3-52, trp1-289, leu2-3,112,his3Δ1; MAL2-8c; SUC2*	Wild type yeast strain	(Entian and Kötter, 2007)
JCR-27		*CEN.PK2-1D ChrXII-2Δ::HphMX_PTEF1_cas9_TCYC1, ChrXI-3Δ::PGAL1_ERG8_TCYC1, PGAL10_tHMG1_TGAL10, PGAL7_ERG12_TADH1, ChrX-3Δ::PGAL1_ERG13_TCYC1, PGAL10_tHMG1_THMG1, ChrXII-4Δ::PGAL1_IDI1_TCYC1, PGAL10_ERG10_TGAL10, PGAL7_MVD1_TADH1*	terpene precursor-providing yeast strain	(Siemon et al., 2020)
YZL-141		*gal1Δ, gal7Δ, gal10Δ:: TRP1_PGAL10-tHMG1*	terpene precursor-providing yeast strain	*(Ma et al., 2019)*
yCL1	CEN.PK2-1D carrying pCL186	Centromeric LEU2, P_{GPD}–*GbLPS*–T_{ADH1}	diterpene skeleton formation	This study

yCL1N	JCR-27 carrying pAG415GPD-ccdB	Centromeric LEU2, P_{GPD}–*ccdB*–T_{CYC1}	Negative control for yCL1	This study
yCL2	JCR-27 carrying pCL87	2μ (episomal) URA3, P_{GAL10}–*TcGGPPs*–T_{ADH1}; P_{GAL1}–*LPS (ΔN79, M593I/Y700F)*–T_{CYC1}	diterpene skeleton formation	This study
yCL2N	JCR-27 carrying pESC-Ura	2μ (episomal) URA3, bidirectional P_{GAL1}/P_{GAL10}, empty vector.	Negative control for yCL2	This study
yCL3	JCR-27 carrying pCL87 and pCL90	2μ (episomal) URA3, P_{GAL10}–*TcGGPPs*–T_{ADH1}; P_{GAL1}–*LPS(ΔN79, M593I/Y700F)*–T_{CYC1}. 2μ (episomal) LEU2, P_{GAL10}–*GbCPR1*–T_{ADH1}; P_{GAL1}–*Gb_04669*–T_{CYC1}.	Functional characterization of Gb_04669 (CYP450) supported by GbCPR1.	This study
yCL3N	JCR-27 carrying pCL87 and pCL88	2μ (episomal) URA3, P_{GAL10}–*TcGGPPs*–T_{ADH1}; P_{GAL1}–*LPS(ΔN79, M593I/Y700F)*–T_{CYC1}. 2μ (episomal) LEU2, P_{GAL10}–*GbCPR1*–T_{ADH1}.	Negative control for yCL3	This study
yCL4	JCR-27 carrying pCL87 and pCL91	2μ (episomal) URA3, P_{GAL10}–*TcGGPPs*–T_{ADH1}; P_{GAL1}–*LPS(ΔN79, M593I/Y700F)*–T_{CYC1}. 2μ (episomal) LEU2, P_{GAL10}–*GbCPR2*–T_{ADH1}; P_{GAL1}–*Gb_04669*–T_{CYC1}.	Functional characterization of Gb_04669 (CYP450) supported by GbCPR2.	This study
yCL4N	JCR-27 carrying pCL87 and pCL89	2μ (episomal) URA3, P_{GAL10}–*TcGGPPs*–T_{ADH1}; P_{GAL1}–*LPS(ΔN79, M593I/Y700F)*–T_{CYC1}. 2μ (episomal) LEU2, P_{GAL10}–*GbCPR2*–T_{ADH1}.	Negative control for yCL4	This study

yCL5	JCR-27 carrying pCL87 and pCL92	2μ (episomal) URA3, P_{GAL10}–*TcGGPPs*–T_{ADH1}; P_{GAL1}–*LPS(ΔN79, M593I/Y700F)*–T_{CYC1}. 2μ (episomal) LEU2, P_{GAL10}–*AtCPR1*–T_{ADH1}; P_{GAL1}–*Gb_04669*–T_{CYC1}.	Functional characterization of Gb_04669 (CYP450) supported by AtCPR1.	This study
yCL5N	JCR-27 carrying pCL87 and pESC-LEU-AtCPR1	2μ (episomal) URA3, P_{GAL10}–*TcGGPPs*–T_{ADH1}; P_{GAL1}–*LPS(ΔN79, M593I/Y700F)*–T_{CYC1}. 2μ (episomal) LEU2, P_{GAL10}–*AtCPR1*–T_{ADH1}.	Negative control for yCL5	This study
yCL6	JCR-27 carrying pCL334	2μ (episomal) HIS3, P_{GAL10}–SmCPS–T_{ADH1}; P_{GAL1}–CfTPS3–T_{CYC1}.	Formation of the miltiradiene skeleton	This study
yCL6N	JCR-27 carrying pESC-HIS	2μ (episomal) HIS3, bidirectional P_{GAL1}/P_{GAL10}, empty vector	Negative control for yCL6, yCL7	This study
yCL7	JCR-27 carrying pCL335	2μ (episomal) HIS3, P_{GAL10}–SmCPS–T_{ADH1}; P_{GAL1}–SmKSL1–T_{CYC1}.	Formation of the miltiradiene skeleton	This study
yCL8	JCR-27 carrying pCL87, pCL88 and pCL248	2μ (episomal) URA3, P_{GAL10}–*TcGGPPs*–T_{ADH1}; P_{GAL1}–*LPS(ΔN79, M593I/Y700F)*–T_{CYC1}. 2μ (episomal) LEU2, P_{GAL10}–GbCPR1–T_{ADH1}. 2μ (episomal) HIS3, P_{GAL10}–Gb_33836–T_{ADH1}.	Functional characterization of Gb_33836 (CYP450) supported by GbCPR1.	This study
yCL8N	JCR-27 carrying pCL87, pCL88 and pESC-HIS	2μ (episomal) URA3, P_{GAL10}–*TcGGPPs*–T_{ADH1}; P_{GAL1}–*LPS(ΔN79, M593I/Y700F)*–T_{CYC1}. 2μ (episomal) LEU2, P_{GAL10}–GbCPR1–T_{ADH1}. 2μ (episomal) HIS3, bidirectional P_{GAL1}/P_{GAL10}, empty vector	Negative control for yCL8	This study

yCL9	JCR-27 carrying pCL433	2µ (episomal) HIS3, P_{GAL10}–SmCPS–T_{ADH1}	Formation of the (+)-copalol	This study
yCL9N	JCR-27 carrying pESC-HIS	2µ (episomal) HIS3, bidirectional P_{GAL1}/P_{GAL10}, empty vector	Negative control for yCL9	This study
yCL10	JCR-27 carrying pCL433, pCL418 and pCL342	2µ (episomal) HIS3, P_{GAL10}–SmCPS–T_{ADH1}; 2µ (episomal) URA3, P_{GAL10}–*TcGGPPs*–T_{ADH1}; 2µ (episomal) LEU2, P_{GAL10}–GbCPR1–T_{ADH1}; P_{GAL1}–*Gb_33836*–T_{CYC1}	Functional characterization of Gb_33836 (CYP450) supported by GbCPR1.	This study
yCL10N	JCR-27 carrying pCL433, pCL418 and pCL88	2µ (episomal) HIS3, P_{GAL10}–SmCPS–T_{ADH1}; 2µ (episomal) URA3, P_{GAL10}–*TcGGPPs*–T_{ADH1}; 2µ (episomal) LEU2, P_{GAL10}–GbCPR1–T_{ADH1}	Negative control for yCL10	This study

Table S2. Linkage analysis: Numerous P450 genes are distributed in the vicinity of GbLPS.

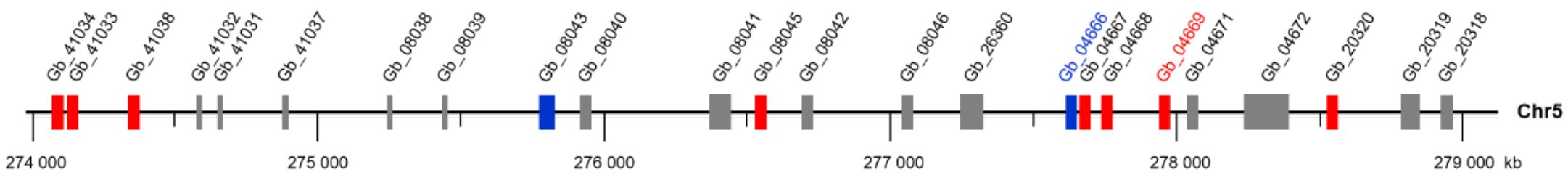

Gene	Annotation
Gb_41034	ATG29904.1(CYP867B5 [Taxus chinensis])
Gb_41033	ATG29904.1(CYP867B5 [Taxus chinensis])
Gb_41038	ATG29932.1(CYP750C17 [Taxus chinensis])
Gb_41032	RWR88624.1(transposon Tf2-1 polyprotein [Cinnamomum micranthum f. kanehirae])
Gb_41031	-
Gb_41037	ABK25004.1(unknown [Picea sitchensis])
Gb_08038	PTQ27689.1(hypothetical protein MARPO_0187s0012 [Marchantia polymorpha])
Gb_08039	-
Gb_08043	AAR02861.1(taxadiene synthase [Taxus baccata])
Gb_08040	-
Gb_08041	-
Gb_08045	ATG29904.1(CYP867B5 [Taxus chinensis])
Gb_08042	-
Gb_08046	ABK25147.1(unknown [Picea sitchensis])
Gb_26360	RWR85496.1(putative receptor-like protein kinase isoform X1 [Cinnamomum micranthum f. kanehirae])
Gb_04666	Bifunctional levopimaradiene synthase
Gb_04667	PWA76955.1(cytochrome P450 [Artemisia annua])
Gb_04668	XP_012066128.1(abscisic acid 8'-hydroxylase 3 [Jatropha curcas])
Gb_04669	XP_010063878.1(abscisic acid 8'-hydroxylase 3 [Eucalyptus grandis])
Gb_04671	XP_010250834.1(transcription factor GTE9 [Nelumbo nucifera])
Gb_04672	ABK24599.1(unknown [Picea sitchensis])
Gb_20320	ATG29904.1(CYP867B5 [Taxus chinensis])
Gb_20319	ADE76625.1(unknown [Picea sitchensis])
Gb_20318	ADE76314.1(unknown [Picea sitchensis])

Table S3. Distribution of P450 genes within ±10 genes of known MVA pathway genes in linkage analysis

Gene of MVA pathway	Abbreviation	Location in genome	CYP450 (Left 10 genes)	CYP450 (Right 10 genes)
Acetyl-CoA C-acetyltransferase	**ACAT**	Gb_28732 (Chr4)	-	-
3-Hydroxy-3-methylglutaryl coenzyme A reductase	**HMGR**	Gb_26601 (Chr10)	-	-
	**HMGR1**	Gb_13780 (Chr10)	-	-
	**HMGR2**			
Mevalonate kinase	**MVK**	Gb_20761 (Chr9)	Gb_20775 (KO1, 4CDS, 1.4Mb)	-
Phosphomevalonate kinase	**PMK**	Gb_31215 (Chr11)	-	-
Mevalonate disphosphate decarboxylase	**MVD**	Gb_31698 (Chr11)	-	-
1-Hydroxy-2-methyl-2-(E)-butenyl 4-diphosphate reductase	**IDS1**	Gb_21731 (Chr3)	-	-
	**IDS2**	Gb_41470 (Chr10)	-	-
	**IDS3**	Gb_23677 (Chr6)	-	
Farnesyl diphosphate synthetase	**FPS**	Gb_29808-Gb_29812 (Chr3)	-	-

Table S4. Candidate CYP450s used in this Study

plasmid	Gene	Discription	Source
pCL187	pESC-Trp-Gb_03394	Cytochrome P450 monooxygenases	This Study
pCL188	pESC-Trp-Gb_41418	Cytochrome P450 monooxygenases	This Study
pCL189	pESC-Trp-Gb_01407	Cytochrome P450 monooxygenases	This Study
pCL190	pESC-Trp-Gb_41167	Cytochrome P450 monooxygenases	This Study
pCL191	pESC-Trp-Gb_01677	Cytochrome P450 monooxygenases	This Study
pCL192	pESC-Trp-Gb_28864	Cytochrome P450 monooxygenases	This Study
pCL193	pESC-Trp-Gb_26944	Cytochrome P450 monooxygenases	This Study
pCL194	pESC-Trp-Gb_36690	Cytochrome P450 monooxygenases	This Study
pCL195	pESC-Trp-Gb_31078	Cytochrome P450 monooxygenases	This Study
pCL196	pESC-Trp-Gb_30278	Cytochrome P450 monooxygenases	This Study
pCL197	pESC-Trp-Gb_41080	Cytochrome P450 monooxygenases	This Study
pCL198	pESC-Trp-Gb_08045	Cytochrome P450 monooxygenases	This Study
pCL199	pESC-Trp-Gb_24737	Cytochrome P450 monooxygenases	This Study
pCL200	pESC-Trp-Gb_32517	Cytochrome P450 monooxygenases	This Study
pCL201	pESC-Trp-Gb_41080	Cytochrome P450 monooxygenases	This Study
pCL202	pESC-Trp-Gb_27584	Cytochrome P450 monooxygenases	This Study
pCL203	pESC-Trp-Gb_34708	Cytochrome P450 monooxygenases	This Study
pCL204	pESC-Trp-Gb_02415	Cytochrome P450 monooxygenases	This Study
pCL205	pESC-His-Gb_17780	Cytochrome P450 monooxygenases	This Study
pCL206	pESC-His-Gb_05138	Cytochrome P450 monooxygenases	This Study
pCL207	pESC-His-Gb_16620	Cytochrome P450 monooxygenases	This Study
pCL208	pESC-His-Gb_02234	Cytochrome P450 monooxygenases	This Study
pCL209	pESC-His-Gb_30635	Cytochrome P450 monooxygenases	This Study
pCL210	pESC-His-Gb_28422	Cytochrome P450 monooxygenases	This Study
pCL211	pESC-His-Gb_31083	Cytochrome P450 monooxygenases	This Study
pCL212	pESC-His-Gb_04622	Cytochrome P450 monooxygenases	This Study
pCL213	pESC-His-Gb_27803	Cytochrome P450 monooxygenases	This Study

pCL214	pESC-His-Gb_00367	Cytochrome P450 monooxygenases	This Study
pCL215	pESC-His-Gb_02300	Cytochrome P450 monooxygenases	This Study
pCL216	pESC-His-Gb_33612	Cytochrome P450 monooxygenases	This Study
pCL217	pESC-His-Gb_12030	Cytochrome P450 monooxygenases	This Study
pCL218	pESC-His-Gb_34291	Cytochrome P450 monooxygenases	This Study
pCL219	pESC-His-Gb_33310	Cytochrome P450 monooxygenases	This Study
pCL220	pESC-His-Gb_05270	Cytochrome P450 monooxygenases	This Study
pCL221	pESC-His-Gb_06435	Cytochrome P450 monooxygenases	This Study
pCL222	pESC-His-Gb_08057	Cytochrome P450 monooxygenases	This Study
pCL223	pESC-His-Gb_08112	Cytochrome P450 monooxygenases	This Study
pCL224	pESC-His-Gb_09401	Cytochrome P450 monooxygenases	This Study
pCL225	pESC-His-Gb_13160	Cytochrome P450 monooxygenases	This Study
pCL226	pESC-His-Gb_13597	Cytochrome P450 monooxygenases	This Study
pCL227	pESC-His-Gb_05603	Cytochrome P450 monooxygenases	This Study
pCL228	pESC-His-Gb_14584	Cytochrome P450 monooxygenases	This Study
pCL229	pESC-His-Gb_23944	Cytochrome P450 monooxygenases	This Study
pCL230	pESC-His-Gb_35763	Cytochrome P450 monooxygenases	This Study
pCL231	pESC-His-Gb_09402	Cytochrome P450 monooxygenases	This Study
pCL232	pESC-His-Gb_11421	Cytochrome P450 monooxygenases	This Study
pCL233	pESC-His-Gb_11694	Cytochrome P450 monooxygenases	This Study
pCL234	pESC-His-Gb_14719	Cytochrome P450 monooxygenases	This Study
pCL235	pESC-His-Gb_15030	Cytochrome P450 monooxygenases	This Study
pCL236	pESC-His-Gb_23185	Cytochrome P450 monooxygenases	This Study
pCL237	pESC-His-Gb_25247	Cytochrome P450 monooxygenases	This Study
pCL238	pESC-His-Gb_25258	Cytochrome P450 monooxygenases	This Study
pCL239	pESC-His-Gb_25584	Cytochrome P450 monooxygenases	This Study
pCL240	pESC-His-Gb_30635	Cytochrome P450 monooxygenases	This Study
pCL241	pESC-His-Gb_33488	Cytochrome P450 monooxygenases	This Study
pCL242	pESC-His-Gb_33489	Cytochrome P450 monooxygenases	This Study

pCL243	pESC-His-Gb_36644	Cytochrome P450 monooxygenases	This Study
pCL244	pESC-His-Gb_41653	Cytochrome P450 monooxygenases	This Study
pCL245	pESC-His-Gb_08058	Cytochrome P450 monooxygenases	This Study
pCL246	pESC-His-Gb_19884	Cytochrome P450 monooxygenases	This Study
pCL248	pESC-His-Gb_33836	Cytochrome P450 monooxygenases	This Study
pCL249	pESC-His-Gb_30412	Cytochrome P450 monooxygenases	This Study
pCL250	pESC-His-Gb_11069	Cytochrome P450 monooxygenases	This Study
pCL251	pESC-His-Gb_14802	Cytochrome P450 monooxygenases	This Study
pCL252	pESC-His-Gb_09401	Cytochrome P450 monooxygenases	This Study
pCL253	pESC-His-Gb_20320	Cytochrome P450 monooxygenases	This Study
pCL255	pESC-His-Gb_14734	Cytochrome P450 monooxygenases	This Study
pCL256	pESC-His-Gb_03772	Cytochrome P450 monooxygenases	This Study
pCL257	pESC-His-Gb_05630	Cytochrome P450 monooxygenases	This Study
pCL258	pESC-His-Gb_14581	Cytochrome P450 monooxygenases	This Study
pCL259	pESC-His-Gb_14594	Cytochrome P450 monooxygenases	This Study
pCL260	pESC-His-Gb_05990	Cytochrome P450 monooxygenases	This Study
pCL261	pESC-His-Gb_36689	Cytochrome P450 monooxygenases	This Study
pCL262	pESC-His-Gb_11087	Cytochrome P450 monooxygenases	This Study
pCL263	pESC-His-Gb_12032	Cytochrome P450 monooxygenases	This Study
pCL264	pESC-His-Gb_04557	Cytochrome P450 monooxygenases	This Study
pCL265	pESC-His-Gb_10868	Cytochrome P450 monooxygenases	This Study
pCL266	pESC-His-Gb_22494	Cytochrome P450 monooxygenases	This Study
pCL267	pESC-His-Gb_19886	Cytochrome P450 monooxygenases	This Study
pCL268	pESC-His-Gb_41034	Cytochrome P450 monooxygenases	This Study
pCL269	pESC-His-Gb_10753	Cytochrome P450 monooxygenases	This Study
pCL270	pESC-His-Gb_21585	Cytochrome P450 monooxygenases	This Study
pCL271	pESC-His-Gb_19885	Cytochrome P450 monooxygenases	This Study
pCL272	pESC-His-Gb_21485	Cytochrome P450 monooxygenases	This Study
pCL273	pESC-His-Gb_20775	Cytochrome P450 monooxygenases	This Study

pCL274	pESC-His-Gb_19218	Cytochrome P450 monooxygenases	This Study
pCL275	pESC-His-Gb_08355	Cytochrome P450 monooxygenases	This Study
pCL276	pESC-His-Gb_19877	Cytochrome P450 monooxygenases	This Study
pCL277	pESC-His-Gb_33309	Cytochrome P450 monooxygenases	This Study
pCL278	pESC-His-Gb_30307new	Cytochrome P450 monooxygenases	This Study
pCL279	pESC-His-Gb_06028	Cytochrome P450 monooxygenases	This Study
pCL280	pESC-His-Gb_01225	Cytochrome P450 monooxygenases	This Study
pCL281	pESC-His-Gb_08111	Cytochrome P450 monooxygenases	This Study
pCL282	pESC-His-Gb_07574	Cytochrome P450 monooxygenases	This Study
pCL283	pESC-His-GbCYP7005C1	Cytochrome P450 monooxygenases	This Study
pCL284	pESC-His-GbCYP867E38	Cytochrome P450 monooxygenases	This Study
pCL285	pESC-His-Gb_24246	Cytochrome P450 monooxygenases	This Study
pCL286	pESC-His-Gb_12034	Cytochrome P450 monooxygenases	This Study
pCL287	pESC-His-Gb_20267	Cytochrome P450 monooxygenases	This Study
pCL288	pESC-His-Gb_10754	Cytochrome P450 monooxygenases	This Study
pCL305	pESC-His-Gb_02300	Cytochrome P450 monooxygenases	This Study
pCL306	pESC-His-Gb_12228	Cytochrome P450 monooxygenases	This Study
pCL307	pESC-His-Gb_12714	Cytochrome P450 monooxygenases	This Study
pCL308	pESC-His-Gb_36429	Cytochrome P450 monooxygenases	This Study
pCL309	pESC-His-Gb_28315	Cytochrome P450 monooxygenases	This Study
pCL310	pESC-His-Gb_28863	Cytochrome P450 monooxygenases	This Study
pCL311	pESC-His-Gb_37719	Cytochrome P450 monooxygenases	This Study
pCL312	pESC-His-Gb_28694	Cytochrome P450 monooxygenases	This Study
pCL313	pESC-His-Gb_13157	Cytochrome P450 monooxygenases	This Study
pCL314	pESC-His-Gb_05137	Cytochrome P450 monooxygenases	This Study
pCL315	pESC-His-Gb_41038	Cytochrome P450 monooxygenases	This Study
pCL316	pESC-His-Gb_41086	Cytochrome P450 monooxygenases	This Study
pCL317	pESC-His-Gb_15955	Cytochrome P450 monooxygenases	This Study
pCL318	pESC-His-Gb_27513	Cytochrome P450 monooxygenases	This Study

pCL319	pESC-His-Gb_10755	Cytochrome P450 monooxygenases	This Study
pCL320	pESC-His-Gb_11826	Cytochrome P450 monooxygenases	This Study
pCL321	pESC-His-Gb_16765	Cytochrome P450 monooxygenases	This Study

Table S5. Synthetic coding sequences of genes used for functional characterization in this study.

Gene	Sequence (5′–3′)
**GbLPS**	ATGCTTAATGCGGATTATCATCCAGCTGTCTGGAAGGACGATTTCATCGACTCTCTA<br>ACATCCCCTAATTCCCACGCGACATCGAAATCAAGCGTCGATGAGACAATCAATAA<br>AAGAATCCAGACATTGGTGAAGGAAATCCAGTGCATGTTTCAGTCCATGGGCGAC<br>GGTGAAACGAATCCATCTGCATATGATACAGCTTGGGTGGCAAGAATTCCGTCAAT<br>TGACGGCTCTGGTGCACCCCAATTTCCCCAAACGCTTCAATGGATTCTGAACAATC<br>AACTGCCAGATGGCTCGTGGGGTGAGGAGTGCATTTTTCTGGCGTATGACAGAGTT<br>TTAAACACTCTCGCCTGCCTCCTCACTCTCAAAATATGGAATAAGGGCGACATTCA<br>AGTGCAGAAAGGGGTTGAGTTTGTGAGAAAACACATGGAAGAAATGAAGGACGA<br>AGCTGACAATCACAGGCCAAGTGGATTCGAGGTCGTGTTTCCTGCAATGTTAGATG<br>AAGCAAAAAGCTTGGGATTGGATCTTCCTTATCACCTCCCTTTCATCTACCAAATCC<br>ACCAAAAGCGCCAGAAAAAGCTTCAAAAGATTCCCCTCAATGTTCTTCATAACCAT<br>CAGACGGCGTTGCTCTACTCTCTGGAGGGTTTGCAAGATGTGGTGGACTGGCAAGA<br>GATCACAAATCTTCAATCAAGAGACGGATCATTTTTAAGCTCCCCTGCATCTACTG<br>CTTGTGTCTTCATGCACACTCAAAACAAACGATGCCTCCACTTTCTCAACTTCGTGC<br>TCAGCAAATTTGGCGACTACGTTCCTTGCCATTACCCACTTGATCTATTTGAACGCC<br>TCTGGGCTGTCGATACAGTTGAACGCTTGGGAATCGATCGCTATTTCAAGAAAGAA<br>ATCAAAGAATCTCTGGATTACGTTTATAGGTACTGGGACGCCGAAAGAGGCGTGG<br>GATGGGCAAGATGCAATCCTATTCCTGATGTCGATGACACTGCCATGGGTCTTAGA<br>ATCCTGAGACTTCATGGATACAATGTATCTTCAGATGTTCTGGAGAATTTCAGAGA<br>CGAGAAAGGAGACTTCTTTTGCTTTGCCGGTCAAACGCAAATTGGTGTGACCGATA<br>ATCTTAACCTTTATAGATGTTCACAAGTATGTTTTCCGGGAGAAAAGATAATGGAA<br>GAAGCTAAGACCTTCACTACAAATCATCTCCAAAATGCTCTTGCCAAAAACAACGC<br>ATTTGATAAGTGGGCTGTCAAGAAGGATCTTCCTGGAGAGGTGGAGTATGCTATAA<br>AGTATCCGTGGCATAGAAGTATGCCAAGATTGGAGGCAAGAAGTTACATAGAGCA<br>ATTTGGATCAAATGATGTCTGGCTGGGGAAGACTGTGTATAAGATGCTATATGTGA<br>GCAACGAAAAATATTTGGAGCTGGCCAAATTGGACTTCAATATGGTGCAGGCCTTA<br>CACCAAAAGGAGACTCAACACATTGTCAGCTGGTGGAGAGAATCGGGATTCAATG<br>ATCTTACATTCACCCGCCAGCGGCCTGTGGAAATCTATTTCTCAGTGGCGGTTAGTA<br>TGTTTGAGCCAGAATTCGCTGCTTGTAGAATTGCCTATGCCAAGACTTCTTGCCTCG<br>CAGTTATTCTAGACGATCTTTACGACACCCACGGATCTCTGGATGATCTTAAATTGT<br>TCTCTGAAGCGGTCCGAAGATGGGATATCTCTGTGCTGGATAGCGTTCGGGATAAT<br>CAGTTGAAAGTTTGCTTCCTAGGGCTGTACAACACAGTGAATGGATTTGGAAAAGA<br>TGGACTCAAGGAACAAGGCCGTGATGTGCTGGGCTATCTTCGAAAAGTATGGGAG<br>GGCTTGCTCGCATCGTTCACCAAAGAAGCCGAATGGTCGGCAGCAAAGTATGTGCC<br>GACATTCAACGAATATGTGGAAAATGCCAAAGTGTCCATAGCACTTGCGACAGTCG<br>TACTAAACTCAATCTTTTTCACTGGAGAATTACTTCCTGATTACATTTTACAGCAAG<br>TAGACCTTCGGTCCAAATTTCTGCATCTTGTGTCTTTGACTGGACGACTAATCAATG<br>ACACCAAGACTTACCAGGCCGAGAGAAACCGTGGTGAATTGGTTTCCAGCGTACA<br>GTGCTACATGAGGGAAAATCCGGAGTGCACAGAGGAAGAAGCTCTAAGTCATGTT<br>TATGGTATCATCGACAACGCACTGAAGGAATTGAATTGGGAGTTGGCCAACCCAGC<br>GAGCAATGCCCCATTGTGTGTGAGAAGACTGCTGTTCAACACTGCAAGAGTGATGC<br>AGCTGTTTTATATGTACAGAGATGGCTTTGGTATCTCTGACAAAGAGATGAAAGAC<br>CATGTCAGCCGAACTCTTTTCGATCCTGTGGCGTAG
**TcGGPPS**	ATGGCATACACCGCTATGGCTGCTGGTACTCAATCTTTGCAATTGAGAACTGTTGCT<br>TCCTACCAAGAATGTAACTCTATGAGATCTTGTTTTAAGTTGACTCCATTTAAGTCT<br>TTCCATGGTGTTAATTTTAACGTTCCTTCTTTGGGTGCTGCTAACTGTGAAATTATG<br>GGTCATTTGAAATTGGGTTCTTTGCCATATAAGCAATGTAGTGTTTCTTCAAAATCC<br>ACTAAGACTATGGCTCAATTGGTTGATTTAGCTGAAACTGAAAAAGCTGAAGGTAA<br>AGATATTGAATTCGATTTTAACGAATACATGAAGTCAAAGGCTGTTGCTGTTGATG<br>CTGCTTTGGATAAAGCTATTCCATTGGAATATCCAGAAAAAATTCATGAATCTATG<br>AGATATTCTTTGTTGGCTGGTGGTAAAAGAGTTAGACCAGCTTTGTGTATTGCTGCT<br>TGTGAATTGGTTGGTGGTTCTCAAGATTTGGCTATGCCAACTGCTTGTGCTATGGAA<br>ATGATTCATACTATGTCTTTGATTCATGATGATTTGCCATGTATGGATAATGATGAT<br>TTTAGAAGAGGTAAACCAACTAATCATAAAGTTTTTGGTGAAGATACTGCTGTTTT<br>GGCTGGTGATGCTTTGTTGTCTTTTGCTTTTGAACATATTGCTGTTGCTACTTCTAAA<br>ACTGTTCCATCTGATAGAACTTTGAGAGTTATTTCTGAATTGGGTAAAACTATTGGT<br>TCTCAAGGTTTGGTTGGTGGTCAAGTTGTTGATATTACTTCTGAAGGTGATGCTAAT

	GTTGATTTGAAAACTTTGGAATGGATTCATATTCATAAAACTGCTGTTTTGTTGGAA<br>TGTTCTGTTGTTTCTGGTGGTATTTTGGGTGGTGCTACTGAAGATGAAATTGCTAGA<br>ATTAGAAGATATGCTAGATGTGTTGGTTTGTTGTTTCAAGTTGTTGATGATATTTTG<br>GATGTTACTAAATCTTCTGAAGAATTGGGTAAAACTGCTGGTAAAGATTTGTTGAC<br>TGATAAAGCTACTTATCCAAAATTGATGGGTTTGGAAAAAGCTAAAGAATTTGCTG<br>CTGAATTGGCTACTAGAGCTAAAGAAGAATTGTCTTCTTTTGATCAAATTAAAGCT<br>GCTCCATTGTTGGGTTTGGCTGATTATATTGCTTTTAGACAAAATTAA
**Gb_CPR1**	ATGCAGACAAATTCGAACACCGTAGAGAGTGCCCAGGGTCTGGGAAAGAGTTTAT<br>TGGAAATATCTCGCCTGCATCAGATATTGGCCTTGGTGAATAGCAAAGGAGATCTG<br>ACGACTATGACGGGGTCTACGTTGATTTTGAGAGACAATCCTCAATTGTTAATGTT<br>GTTGACGACTTCTCTGGCCGTAGCGATTACATGCCTTTTGCTGTTGATATGGCGAAG<br>ATCAGGATCTAGCGTGTCAAAATCACCAGAGAAGCCGACGCCTTTGGTATCAGAG<br>AAAGAGCCGGAGACAGAAGACGATGTTGGGAAGAAAAAAGTGACGGTTTTCTTTG<br>GTACTCAAACTGGTACTGCTGAAGGATTTGCCAAGGCTCTAGCTGAGGAGGGTAAA<br>GCGAGATACGATAAGGCGATCTTTAAAGTAGTAGATCTGGATGACTATGCAGCAG<br>ACGACGACGAGTACGAAGAGAAATTAAAGAAGGAAAATTTAGCTTTCTTTATGCT<br>GGCAACGTATGGAGATGGGGAGCCCACAGATAATGCGGCCAGGTTTTACAAGTGG<br>TTTGCTGAGGGAAAAGAGAGAGGATCTTGGCTTTCGAATCTCACATATGGTGTATT<br>TGGATTAGGCAATAGACAGTATGAGCATTTTAATAAGGTGGCCAAGACTGTAGATG<br>AAGTATTAAATGAGCAAGGTGCGAAGCAGCTTGTTCCATTGGGTCTTGGAGATGAT<br>GATCAGTGCATTGAGGATGACTTTACATCCTGGAGAGAGCAGGTGTGGCCTGAGCT<br>GGATTTGCTACTACGTGATGATAGTGATGAGCCGACAAGGGCTACACCTTACACTG<br>CTGCCGTGCTTGAGTATCGGGTAGAAATTCATGAATCTTCGGTTCCACTGTATGAG<br>GAAACTTATGCAAATAAGCAGAATGGTCAAGCTGTGTATGATCTTCATCACCCATG<br>CAGAGCAAATGTAGCAGTCCGGAGGGAGCTTCATACTCCTTTGTCTGACCGTTCTT<br>GTATCCATTTGGAATTTGAAATCTCAGACACTGGTCTTATGTATGAAACTGGAGAT<br>CATGTTGGTGTCTATGCAGAAAATAGTGCTGAAACTGTGGATGAAGCAGCAAAGTT<br>ATTAGGGTACCCACTAGACACTGTATTCTCAGTTCATATTGACAAAGAGGATGGCA<br>CACCACTTGGAGGAAGCTCCTTGCCACCGCCTTTTCCTGGTCCATGTACCTTACAAA<br>CTGCTCTTGCGCATTATGCAGATTTGTTAAATCCTCCTAGAAAGGCTGCTCTGGTTG<br>CGTTGGCTGCCCATGCCTCTGATCCAGCTGAAGCAGAGCGGTTGAAGTTCCTCTCA<br>TCACCGTCTGGAAAGGATGAGTATTCCCAATGGATTATGGGCAGTCAAAGAAGTCT<br>TTTAGAAGTGATGAAAGAGTTCCCATCAGCAAAGCTGCCTCTTGGTGTCTTCTTTGC<br>AGCAGTATCCCCTCGTCTACCACCTCGATATTATTCTATTTCTTCCTCTCCCAGGTTT<br>TCACCATCTAGGATACATGTCACATGTGCTCTAGTTTATGGACCCACCCCAACTGGT<br>AGAATTCACAAAGGTGTTTGTTCTACCTGGATGAAGAATTCCTTGCCTTCTGAAGA<br>AAGCCATGATTGTAGCTGGGCACCAATCTTTGTTAGACAGTCAAATTTCAAATTAC<br>CAGCAGATTCTTCTGTTCCTATTGTTATGGTGGGCCCTGGAACTGGTTTGGCACCTT<br>TTAGAGGCTTTTTGCAGGAAAGAGCGGCACTTAAAGAATCTGGTGAGAAACTTGG<br>ACCAGCTGTTCTCTTTTTTGGGTGCAGGAATCGCCAAATGGACTACATTTATGAAG<br>ATGAACTGAAAGGCTTTGTAGAGAAAGGAGCACTGACAGACCTCATTGTCGCTTTC<br>TCTCGCGAAGGAACAACCAAGGAGTATGTGCAGCACAAGTTGGTGGAGAAGGCAT<br>CTTATATCTGGAATCTTATTTCTCAAGGTGGTTATCTGTATGTATGTGGTGATGCAA<br>AAGGCATGGCCAGGGATGTCCATAGGACATTACACACTGTTGTGCAAGAGCAAGA<br>ATCTGTGGATAGCACCAAAGCAGAGGCTATAGTGAAGAAATTACAGACAGATGGA<br>AGGTATTTGCGAGATGTATGGCATCATCATCATCATCATTGA
**Gb_CPR2**	ATGGGTAATATGTTGTCTTTTGTTTCTTCTTCTTTGAATCCATCTTCTTTGCAAGTTG<br>ATCCATTTAATAAATTGTCTTTTACTGGTTCTTTTGCTATGGATCCAATTCCATTGGC<br>TTTGTCTACTTCTTTGTTGATTTTGTCTTTTTCTATTATTATTGGTTCTATTGCTTTGG<br>TTTTTTTGAGATTTTTGCCATCTAATGCTAATGTTTCTAAAGCTGTTCAAATTAAAG<br>AAAAATCTACTACTGTTGAAATTTTGAAAAATCCATTTCCACAACAACCAGATAAA<br>AAAACTGTTATGGTTTTTTTTGGTACTCAAACTGGTACTGCTGAAAGATTTGCTCAA<br>GAATTGGCTGAAGAAGGTAAAATGAGATATAATGCTAAAGTTACTTTTAAAGCTGT<br>TGATTTGGAACAATATGGTGAAGATGATGATGAATATGAAACTAAATTGGGTAAA<br>GAAAAATTTGCTATTTTTACTTTGGCTACTTATGGTGATGGTGAACCAACTGATAAT<br>GCTCAAAGATTTTATAAATGGATTACTCAAGAAGGTGGTAGAAAAGAAGATTGGTT<br>GTCTGGTTTGACTTATGCTGTTTTTGGTTTGGGTAATAAACAATATGAACATTTTAA<br>TAAAACTGGTTTGACTGTTGATGAAGCTTTGTCTAAACAAGGTGCTAAAAGAGTTA<br>TTTCTTGTGGTTTGGGTGATGATGAAAATATGATTGAAGATGATTTTGCTGCTTGGA<br>GAGAACAATTGTGGCCAGAATTGGATGCTTTGATTAGAGATGAAGATGATGAACC

	AACTTGTTCTACTCCATATACTGCTACTATTCCAAAATATAGATTGGTTATTCATGA<br>TAAAAATGCTGTTATTAATGGTGAACCATATGCTCCAAGAAGAAATGGTGAAGCTA<br>TTTTTGATGTTCATCATCCATATAGAACTTCTGTTTTGGTTAGAAGAGAATTGCATA<br>CTCCATTGTCTGATAGATCTTGTACTCATTTGGAATTTGATATTGCTGGTTCTGGTTT<br>GACTTATGAAGCTGGTGATCATGTTGGTGTTTATGCTGAAAATGAACAAGAAACTA<br>TTGAAACTGCTGCTAAAATTTTGGGTTGTCCATTGGATACTGTTTTTTCTTTGCATA<br>AAGATTCTGAAACTGGTGAACCATTGCCAGGTTGTGATTCTTTGCCAGTTCCATTTC<br>CAGCTCCATGTACTTTGCAAACTGCTTTGGCTAGATATGCTGATTTGTTGAATCCAC<br>CAAGAAAAGGTGCTTTGGCTGCTTTGGCTGCTTATGCTTCTGATAATAAAGAAGCT<br>GATCAATTGAAAAGATTGTCTTCTTCTGAAGGTAAAGATGAATATTCTCAATGGAT<br>TTTGTCTTGTCAAAGATCTTTGTTGGAAGTTTTGGCTGATTTTCCATCTGTTAATGTT<br>CCATTGGAAGTTTTTTTTGGTGCTGTTGCTCCAAGATTGTTGCCAAGATTTTATTCTA<br>TTTCTTCTTCTCCATCTTTTGCTCCAACTGGTATTCATGTTACTTGTTCTTTGGTTCA<br>AGGTCCATCTCCAACTGGTAGAATTCATCATGGTGTTTGTTCTACTTGGATGAAAA<br>AATCTATTCCATATACTGAAGAAGGTAATGAATGTTCTTCTGCTCCAATTTTTATTA<br>GACAATCTAATTTTAAATTGCCAGCTGATCCAACTATTCCAATTATTATGGTTGGTC<br>CAGGTACTGGTTTTGCTCCATTTAGAGGTTTTTTGCAAGAAAGAGCTGCTTTGAAA<br>AAATCTGGTTTGCAATTGGGTACTGCTGCTTTGTTTTTTGGTTGTAGAAATAGAAAA<br>CAAGATTTTATTTATGAAGAAGAATTGGCTCAATTTTTGGAACAAGGTATTTTGTCT<br>AGATTGGATGTTGCTTTTTCTAGAGAAGGTCCAAAAAAAGAATATGTTCAAAATAA<br>AATTTTGGAAGAAGCTTCTTATGTTTGGAAATTGGTTTCTGAAGGTGGTTATTTTTA<br>TGTTTGTGGTGATGCTAAAGGTATGGCTAGAGATGTTCATCAAACTTTGATTAATAT<br>TATTCAAGAACAAGAATGTGTTAATGGTTCTGAAGCTGAAAAAGTTTTGAAAAAAT<br>TGCAAACTGAAGGTAGATATTTGAGAGATGTTTGGTAA
**At_CPR1**	ATGACTTCTGCCTTGTATGCCTCTGATTTGTTCAAGCAATTGAAGTCCATTATGGGC<br>ACCGATTCTTTGTCTGATGATGTTGTTTTGGTTATCGCTACTACCTCTTTGGCTTTGG<br>TTGCTGGTTTTGTTGTTCTGTTGTGGAAAAAGACTACCGCTGATAGATCTGGTGAAT<br>TGAAACCATTGATGATCCCCAAATCTTTGATGGCCAAAGATGAAGATGATGACTTG<br>GACTTAGGTTCTGGTAAGACTAGAGTTTCCATTTTCTTCGGTACTCAAACTGGTACT<br>GCTGAAGGTTTTGCTAAAGCTTTGTCCGAAGAAATCAAGGCCAGATACGAAAAAG<br>CTGCCGTTAAGGTTATTGATTTGGATGATTATGCTGCCGATGACGACCAATACGAA<br>GAAAAGTTGAAGAAAGAAACCTTGGCCTTCTTCTGTGTTGCTACTTATGGTGATGG<br>TGAACCTACTGATAATGCTGCTAGATTTTACAAGTGGTTCACCGAAGAGAACGAAA<br>GAGATATCAAGTTGCAACAATTGGCCTACGGTGTTTTTGCTTTGGGTAATAGACAA<br>TACGAGCACTTCAACAAGATCGGTATCGTTTTGGATGAAGAGTTGTGTAAAAAGGG<br>TGCCAAGAGATTGATTGAAGTTGGTTTGGGTGATGATGACCAGTCTATCGAAGATG<br>ATTTTAACGCCTGGAAAGAATCCTTGTGGTCTGAATTGGATAAGTTGTTGAAGGAC<br>GAAGATGACAAATCTGTTGCTACACCATACACTGCTGTTATTCCAGAGTATAGAGT<br>TGTTACTCACGATCCAAGATTCACGACTCAAAAGTCTATGGAATCTAACGTTGCTA<br>ACGGTAACACCACCATCGATATTCATCATCCATGTAGAGTTGATGTCGCCGTCCAA<br>AAAGAATTGCATACTCATGAATCCGACAGATCCTGCATTCATTTGGAATTCGATAT<br>TTCCAGAACCGGTATTACTTACGAAACCGGTGATCATGTTGGTGTTTACGCTGAAA<br>ATCACGTTGAAATCGTTGAAGAAGCCGGTAAGTTGTTAGGTCATTCATTGGATTTG<br>GTGTTCTCCATTCATGCCGACAAAGAAGATGGTTCTCCTTTGGAATCTGCTGTTCCA<br>CCACCATTTCCAGGTCCATGTACTTTAGGTACTGGTTTGGCTAGATATGCTGACTTG<br>TTGAATCCACCAAGAAAGTCTGCTTTAGTTGCTTTGGCTGCTTATGCTACTGAACCA<br>TCTGAAGCCGAAAAATTGAAACATTTGACTTCCCCAGATGGTAAGGACGAATATTC<br>TCAATGGATAGTTGCCTCTCAGAGGTCTTTGTTGGAAGTTATGGCTGCTTTTCCATC<br>TGCTAAACCACCATTGGGTGTTTTTTTTGCTGCTATTGCTCCAAGATTGCAACCTAG<br>GTATTACTCCATTTCTTCATCACCAAGATTGGCCCCATCTAGAGTTCATGTTACATC<br>TGCTTTGGTTTATGGTCCAACTCCAACTGGTAGAATTCATAAGGGTGTTTGTTCTAC<br>CTGGATGAAGAACGCTGTTCCAGCTGAAAAATCTCATGAATGTTCTGGTGCCCCAA<br>TTTTCATTAGAGCTTCTAATTTCAAGCTGCCAAGCAATCCATCTACTCCAATAGTTA<br>TGGTTGGTCCAGGTACAGGTTTAGCTCCTTTTAGAGGTTTCCTACAAGAAAGGATG<br>GCCTTGAAAGAGGATGGCGAAGAATTGGGTTCTTCCTTGTTGTTTTTTGGTTGCAGA<br>AACAGACAGATGGATTTCATCTATGAGGACGAGTTGAACAACTTCGTTGATCAAGG<br>TGTTATCTCCGAATTGATTATGGCCTTTTCTAGAGAAGGTGCCCAGAAAGAATATG<br>TCCAACATAAGATGATGGAAAAAGCCGCTCAAGTTTGGGACCTAATCAAAGAAGA<br>AGGATACTTGTACGTTTGCGGTGATGCTAAAGGTATGGCTAGAGATGTTCATAGAA<br>CATTGCATACCATCGTCCAAGAACAAGAAGGTGTTTCATCTTCTGAAGCTGAAGCT

	ATCGTTAAGAAGTTGCAAACTGAAGGTAGATACTTGAGAGATGTCTGGTGA
**Gb_04669**	ATGGAGTACTTGGATTTAATTCCTACAACGAATGGATATGGTATTTGGGCGGTTGTT GCACCTCTTCTGCTACTCTTATGGTTTATTGTAGGAATGAATAGATCACACAAGTAT TCGACTAAACTTCCGCCGGGCACATTTGGACTACCCATCATAGGGGAATCGTTGGA GTTTCTAAACCACATCAATGGAGATCACATGCATGACTTTGTTGGCAAGAGAAAAG AGAGGTATGGCAACATATTCCGGAGCCACATATTTGGGAAGCCATACCTGTTCGTG ATGGGGCAAAAGGCAGCAAAAATGATGATACAAGGGGATGGAGACCAGTTTCAAG TGTATCAGTTCCACAACGTTAACAAACTCTTAGGTCGTGGCTCGGTCATTCTGGCCT CTGGAGAAGATCACAAGCGCATTCGACATTCCACAAGTGACATCTTCACACCCGAA GGCCTCAGACAATACGTTCTCCTCATTGAGGGAGTGGCTCTCCAATCCATCATGTCT TCATGGACTGTCGGCAAGCTTCTCAATGCCGAAGAGGAGGCCACCAAGTATGCCTT TTATATCACAGCAGAGCTGGTTCTAAGCTGGAAGCCCGGTCCTCTTGTAGATGAAT TCATCCAGCTCTCCAAGGACTTGATGATGGGCATGTTACGAGCGCCCATCAATCTC CCTGGTTTTTCCTATCACAATTCGATCAAAGCAAGGCAGCGGATGGTGTCGATTTTC GAACAGGAGATAAATCGGAGAGAAATGGAGGGTGGGCGTGGTGAAAAGAAAGGA GACATCTTGGAGAGAATGTTGGATGTCCACCGAGAGGGAAATGGAGAGAAATTAT CGAAGCTAGAAATCGTGGACCAGCTCGTTACTTTCATTCTCGCAGGTCATGAGACC ACCGTGGGAACGATTTCGTTTATAGTGAGATACTTGAGTCAGAGGCCCCAATGCCT CGAAACGTTGCGGGCTGAGCACCAAGAGATTCAACGATCCAAACCACCCAACACG CCATTGACTTGGATGGATGTCAAGAGGATGGTTTACACCGACAAGGTTATTAGTGA AGCACTTAGGATGGCTACTATTGTGCCATGGTTGCATAGGAAAGTGAATGAAGACA TGGATTTTGAAGGGTATACGATTCCCAAAGGTTGGATGATAATGCTTGATATTCGA GACACCCACTATGATCCTGACTACTATTCAAACCCTGACGTGTTTACTCCTGAGAG ATTTGACCAGGCGCCAAAGCCATACACATTCTTGCCCTTTTCAGGAGGCAAGAAGA TATGTCTGGGAAAAGATCTTGCGAGATTGGAGATGGCAATTTTCCTTTATCACTTG ACTATCAACTTCAGGTGGGAAGTTGAAGAGGTCGCGAAAGGAATTGAATTTCTCGG AATGGCGAGACCTAAAGGTGGAGTGCCCTTTAGAGTTTTAGAGAAGAAATTACAT GAACTCAACGTCTAA
**Gb_33836**	ATGGCTGATCATTTTTTGTTGTTGTTGACGTTGGGCGTTGTGTGTGTGGCGTTGGTC GTGCAGTTGCAGAGGTGGTTCACGACACGCGTCAGCCCTAAGAAACGCGATGGAG AACACCACAAATTGGTTCCAGGCTCAATGGGATGGCCGTTGATTGGAGAGACTATC AGTTGGTATCTCTGCATGTCCAATCATCATCATCCCCGACACTTCGTCGATGAACGA CAGCGCCGGTATGGAGACATTTTCAGATCCAATTTGTTTGGAAGAAAGGTTATTGT GTCCGTGGATCCCCATTTCAACAGGTATGTGATGCAGAATGAGGGAAGGCTGTTCG AAGCTCGATACCCAAAGCCTTTTGTAGAATTGATGGGCAAATACGGCTTACTCACA GTCCATGGAGAACTGCAAAGAAGGCTTCACGGAACTGCAGTCAATTTGCTCAAGAT AGAGAAGCTTCGAATCCATTTCATGGATGATATTGACTTTATTCTGCACAATGCATT GTCCACATGGGAAGGAAAGAATATACTTCTTCAAGAGGAGTGCCATCGGATGACTT TGAATATGATGGCAAAACAACTGTTGGACTTATCGCCTTCCAAGGAGACGGATGAG ATCGTTAAACAGTTTGACGACTTTTCAAAAGCAGTGCTTACCCTTCCGGTCAAAATT CCAGGCTCAAAATATGCCAGAGGCATTAAGGCTAGGGATTTTCTCAAGAGAAAAG TTTATGAGAAAATAGAGGAGAGAAGGAAAAATCCCCAAATCGTTCATAACGATTT GTTGACAAAACTTCTGAATGAAGAAACCCTTTCTGACGAAATTGTGTCCGATTTTCT TGTATTTCTGCTATTTGCGGGCCACGAGACCTCGTCCAGATCCATGGCTTTCAGTAT CAAGTTTCTTACCGAGTGTCCCAAGGCATTGGAACAATTAAGAGAGGAGCACGAC GCCTTGCATAAGAGAAAAGGCAATGAAAAGCTTACTTGGGATGACTATAAATCTAT GAAATTCACTCAATGCGTCGTTAACGAAACGCTTCGATTAGGGAACATTGCGCCAT TTGTTTACAGAGAAGCAAAAGAAGACATTAAAGTGAAAGATTATCTTATTCCGAAG GGCTCGACAATCATGGCATTCATTTCCGCTGTTCATGTGGACAACAAATACTACCC TGCAGCCCTGAAATTCGACCCATGGCGTTGGGAAGGATACGATCAAGAGGTTTCAA ATAGTCCATCGTATACGCCTTTCGGAGGAGGTGCTAGACTCTGTCCAGGAGCCCAT TTGGGAAGGCTTGAGATGGCGCTTTTTCTTCATCACTTTCTCATCAAGTTTAGGTGG AAGGTTCTTAAAGATGACAAAATCTCCTCCTTTCCTTTTTCTCACTTAGTCGAGGGA TTTCCCATTCAAGTGCAAAGCAGAGAATACATATGTGATTTATCACATAAAGCATA G
**SmCPS**	ATGGCCTCTTTGTCCTCTACCATTTTGTCTAGATCTCCAGCTGCTAGAAGAAGAATT ACTCCAGCTTCTGCTAAATTGCATAGACCAGAATGTTTTGCTACTTCTGCTTGGATG GGTTCCTCTTCTAAAAACTTGTCTTTGAGCTACCAGCTGAACCACAAGAAAATTTCT GTTGCTACTGTTGATGCCCCACAAGTTCATGATCACGATGGTACTACTGTTCATCAA GGTCATGATGCCGTTAAGAACATTGAAGATCCAATCGAGTACATCAGAACCTTGTT

	GAGAACTACTGGTGATGGTAGAATTTCCGTTTCTCCATATGATACTGCTTGGGTTGC
	TATGATCAAAGATGTTGAAGGTAGAGATGGTCCACAATTCCCATCTTCATTGGAAT
	GGATCGTCCAAAATCAATTGGAAGATGGTTCTTGGGGTGACCAAAAGTTGTTTTGT
	GTTTACGATAGACTGGTCAACACCATTGCTTGTGTTGTTGCTTTGAGATCTTGGAAT
	GTTCATGCCCATAAGGTTAAGAGAGGTGTCACTTACATCAAAGAAAACGTCGACA
	AATTGATGGAAGGTAACGAAGAACATATGACCTGTGGTTTTGAAGTTGTCTTTCCA
	GCTTTGTTGCAAAAGGCTAAGTCTTTGGGTATTGAGGATTTGCCATATGATTCACCA
	GCTGTTCAAGAAGTTTACCACGTTAGAGAACAGAAGTTGAAGAGAATCCCATTGG
	AAATCATGCACAAGATCCCTACTAGCTTGTTGTTCTCTTTGGAAGGTTTGGAAAATT
	TGGACTGGGACAAGTTGTTGAAGTTGCAATCTGCTGATGGTTCCTTTTTGACTTCTC
	CATCTTCTACTGCTTTCGCCTTTATGCAAACTAAGGACGAAAAGTGCTACCAATTCA
	TCAAGAACACTATCGACACTTTTAATGGTGGTGCTCCACATACTTATCCAGTTGATG
	TTTTTGGTAGATTGTGGGCCATTGATAGATTGCAAAGATTGGGTATCTCCAGGTTTT
	TCGAACCAGAAATTGCTGATTGCTTGTCCCATATTCATAAGTTCTGGACTGATAAG
	GGTGTGTTCTCTGGTAGAGAATCTGAATTCTGCGATATCGATGATACCTCTATGGGT
	ATGAGATTGATGAGAATGCATGGTTACGATGTTGATCCAAACGTCTTGAGAAACTT
	CAAGCAAAAGGACGGTAAATTCTCTTGTTACGGTGGTCAAATGATCGAATCTCCAT
	CTCCAATCTACAACTTGTACAGAGCTTCCCAATTGAGATTTCCAGGTGAAGAAATC
	TTGGAGGATGCTAAGAGATTTGCCTACGATTTCTTGAAAGAGAAGTTGGCTAACAA
	CCAGATATTGGATAAGTGGGTTATCTCTAAACACTTGCCAGACGAAATCAAGTTGG
	GTTTAGAAATGCCATGGTTGGCTACTTTGCCAAGAGTTGAAGCTAAGTACTACATT
	CAATATTACGCCGGTTCTGGTGATGTTTGGATTGGTAAAACACTGTACAGAATGCC
	CGAAATCTCTAACGATACTTACCATGATTTGGCCAAGACCGATTTCAAAAGATGCC
	AAGCTAAACACCAATTCGAGTGGTTGTATATGCAAGAATGGTACGAATCTTGCGGT
	ATCGAAGAATTCGGTATCTCTAGAAAGGACCTGTTGTTGTCTTACTTTTTGGCTACC
	GCTTCCATTTTCGAATTGGAAAGAACTAACGAAAGAATTGCTTGGGCCAAGTCTCA
	AATTATTGCCAAGATGATTACCTCGTTCTTCAACAAAGAAACCACTTCCGAAGAAG
	ATAAGAGAGCCTTGTTGAACGAATTGGGTAACATTAACGGTTTGAACGATACAAAT
	GGTGCAGGTAGAGAAGGTGGTGCTGGTTCTATTGCTTTAGCTACTTTGACTCAATTC
	TTGGAGGGTTTCGATAGATACACCAGACATCAATTGAAGAATGCTTGGTCTGTTTG
	GTTGACCCAATTGCAACATGGTGAAGCTGATGATGCTGAATTATTGACTAACACCT
	TGAACATTTGCGCTGGTCATATTGCCTTCAGGGAAGAAATTTTAGCTCACAATGAG
	TACAAGGCCCTGTCTAACTTGACTTCTAAAATCTGTAGACAGCTGAGCTTCATCCA
	GTCCGAAAAAGAAATGGGTGTAGAAGGTGAAATTGCCGCCAAATCTTCCATTAAG
	AACAAAGAACTGGAAGAGGACATGCAGATGTTGGTTAAGTTGGTTTTGGAAAAGT
	ACGGTGGTATCGACAGAAACATTAAGAAGGCATTTTTGGCTGTTGCCAAGACCTAT
	TACTATAGAGCTTATCATGCTGCCGATACCATTGATACCCATATGTTTAAGGTTTTG
	TTTGAGCCAGTTGCCTAA
**CfTPS3**	ATGTCATCTTTGGCTGGTAACTTGAGAGTTATTCCATTCTCTGGTAACAGAGTTCAA
	ACCAGAACTGGTATTTTGCCAGTTCATCAAACTCCCATGATTACCTCTAAATCTTCT
	GCTGCTGTTAAGTGCTCTTTGACTACTCCAACTGATTTGATGGGTAAGATCAAAGA
	GGTGTTCAACAGAGAAGTTGATACTTCTCCAGCTGCTATGACTACTCATTCTACTGA
	TATTCCATCCAACTTGTGCATCATCGATACCTTGCAAAGATTGGGTATCGACCAAT
	ACTTCCAATCCGAAATTGATGCTGTCTTGCATGATACTTACAGATTGTGGCAATTGA
	AGAAGAAGGACATCTTCTCCGATATTACTACTCATGCTATGGCTTTCAGACTGTTG
	AGAGTCAAAGGTTATGAAGTTGCCTCTGATGAATTGGCTCCATATGCTGATCAAGA
	AAGAATCAACTTGCAAACCATTGATGTTCCAACCGTTGTTGAATTATACAGAGCTG
	CACAAGAAAGGTTGACCGAAGAAGATTCTACTTTGGAAAAGTTGTACGTTTGGACC
	TCTGCTTTCTTGAAACAACAGTTGTTGACTGATGCCATTCCAGATAAGAAGTTGCAT
	AAGCAAGTTGAGTACTACCTGAAGAACTACCACGGTATTTTGGATAGAATGGGTGT
	CAGAAGAAACTTGGACTTGTACGATATCTCCCACTACAAATCTTTGAAGGCTGCTC
	ATAGATTCTACAACTTGTCTAACGAAGATATTTTGGCCTTCGCCAGACAAGATTTC
	AACATTTCTCAAGCCCAACACCAGAAAGAATTGCAACAATTACAAAGATGGTACG
	CCGATTGCAGATTGGATACTTTGAAATTCGGTAGAGATGTTGTCAGGATCGGTAAC
	TTTTTGACTTCTGCTATGATTGGTGACCCAGAATTGTCTGATTTGAGATTGGCTTTC
	GCCAAACACATCGTTTTGGTTACTAGAATCGATGACTTCTTCGATCATGGTGGTCCA
	AAAGAAGAATCCTACGAAATTTTGGAACTGGTCAAAGAGTGGAAAGAAAAACCTG
	CTGGTGAATACGTTTCCGAAGAAGTGGAAATTTTGTTCACCGCTGTTTACAACACC
	GTTAACGAATTGGCTGAAATGGCCCATATTGAACAAGGTAGATCTGTTAAGGATCT
	GTTGGTTAAGTTGTGGGTCGAGATTTTGTCCGTTTTCAGAATTGAATTGGATACCTG

	GACTAACGATACTGCTTTGACTTTGGAAGAATACTTGTCCCAATCCTGGGTTTCTAT<br>TGGTTGCAGAATCTGCATTTTGATCTCCATGCAATTCCAAGGTGTTAAGTTGAGTGA<br>CGAAATGTTGCAGTCTGAAGAATGTACCGATTTGTGCAGATACGTTTCAATGGTTG<br>ATAGGTTGTTGAACGATGTCCAGACCTTCGAAAAAGAACGTAAAGAAAACACCGG<br>CAACTCCGTTTCTTTGTTGCAAGCTGCTCACAAAGATGAGAGAGTTATCAACGAAG<br>AAGAGGCTTGCATTAAGGTCAAAGAATTGGCAGAGTACAACAGGCGTAAGTTGAT<br>GCAAATCGTTTACAAGACCGGTACTATCTTCCCAAGAAAGTGTAAGGATTTGTTCT<br>TGAAGGCTTGCAGAATTGGTTGCTACTTGTATTCTTCTGGTGACGAATTCACTTCCC<br>CACAACAAATGATGGAAGATATGAAGTCCTTGGTCTATGAACCATTGCCAATTTCT<br>CCACCTGAAGCTAACAATGCATCTGGTGAAAAGATGTCTTGCGTGTCTAACTAA
**SmKSL1**	ATGTCCTTGGCTTTTAATCCAGCTGCTACTGCTTTTTCTGGTAATGGTGCTAGATCC<br>AGAAGAGAAAATTTCCCAGTCAAACACGTTACCGTTAGAGGTTTTCCAATGATCAC<br>TAACAAGTCATCCTTCGCTGTTAAGTGTAACTTGACTACTACTGACTTGATGGGTAA<br>GATTGCCGAAAAGTTTAAGGGCGAAGATTCTAATTTTCCAGCTGCCGCTGCTGTTC<br>AACCAGCTGCTGATATTCCATCTAACTTGTGCATTATCGACACCTTGCAAAGATTG<br>GGTGTTGACAGATATTTCAGACCAGAAATCGATACCATCTTGGAAGGTACTTATAG<br>ATTGTGGCAGAGAAAAGAAAGAGCCATCTTTTCTGATACCGCTATTCATGCTATGG<br>CTTTCAGATTATTGAGAGTCAAGGGTTACGAAGTCTCCTCTGAAGAATTGGCTCCA<br>TATGCTGATCAAGAACATGTTGACTTGCAAACCATTGAAGTTGCTACCGTTATTGA<br>GTTGTATAGAGCTGCTCAAGAAAGAACCGGTGAAGAAGAATCTTCCTTGAAAAAG<br>TTGCATGCTTGGACTACTACCTTCTTGAAGCAAAAGTTGTTGACCAACTCCATTCCA<br>GATAAGAAGTTGCATAAGTTGGTCGAGTACTACTTGAAGAACTACCACGGTATTTT<br>GGATAGAATGGGTGTCAGACAAAACTTGGACTTGTACGATATCTCTTACTACAGAA<br>CTGCTAAGGCTGCTAACAGATTCTCTAACTTGAGATCCGAAGATTTCTTGGCTTTCG<br>CTAGACAAGATTTCAACATTTGCCAAGCTCAACACCAGAAAGAATTGCAACAATTG<br>CAGAGATGGTACGCTGATTGCAAATTGGATACTTTGAAGTACGGTAGAGATGTTGT<br>TAGAGTTGCTAACTTCTTGACCTCCGCTATTATTGGTGATCCAGAATTGTCTGATGT<br>CAGAATCGTTTTCGCTCAACACATCGTTTTGGTTACCAGAATCGATGATTTCTTCGA<br>TCACAGAGGTTCTAGAGAAGAGTCCTACAAGATTTTGGAGTTGATCAAAGAGTGG<br>AAAGAAAAACCAGCAGCTGAATACGGTTCTGAAGAAGTCGAAATTTTGTTCACCG<br>CTGTTTACAACACCGTTAACGAATTGGCTGAAAGGGCTCATGTTGAACAAGGTAGA<br>TCTGTTAAGGACTTCCTGATTAAGTTGTGGGTTCAAATCTTGACCATCTTCAAGAGA<br>GAATTGGACACTTGGTCTGATGATACTGCTTTGACTTTGGATGATTACTTGTCTGCT<br>TCCTGGGTTTCTATTGGTTGTAGAATCTGCATCTTGATGTCCATGCAATTCATCGGT<br>ATCAAGTTGTCTGACGAGATGTTGCTATCCGAAGAATGTATTGATTTGTGCAGGCA<br>CGTTAGTATGGTTGATAGGTTGTTGAACGATGTCCAGACCTTTGAAAAAGAGCGTA<br>AAGAAAACACCGGTAACTCTGTTACTTTGTTGTTGGCTGCAAACAAGGACGATTCA<br>TCTTTTACAGAAGAAGAAGCCATCAGGATCGCTAAAGAAATGGCTGAATGTAACA<br>GACGTCAATTGATGCAAATCGTTTACAAGACCGGTACTATCTTCCCAAGACAATGT<br>AAGGACATGTTTTTGAAGGTCTGCAGAATTGGTTGTTACTTGTACGCTTCTGGTGAT<br>GAATTCACTTCTCCACAACAAATGATGGAAGATATGAAGTCCTTGGTCTACGAACC<br>ATTGACTATTCATCCATTGGTTGCCAACAATGTCAGAGGTAAGTAA

Table S6. Plasmids used in the study.

**Plasmids**	**Description**	**Reference/ Source**
pESC-URA	2µ (episomal) URA3, bidirectional P_{GAL1}/P_{GAL10}, empty vector.	Agilent #217454
pESC-HIS	2µ (episomal) HIS3, bidirectional P_{GAL1}/P_{GAL10}, empty vector.	Agilent #217451
pESC-LEU	2µ (episomal) LEU2, bidirectional P_{GAL1}/P_{GAL10}, empty vector.	Agilent #217452
pESC-TRP	2µ (episomal) TRP1, bidirectional P_{GAL1}/P_{GAL10}, empty vector.	Agilent #217453
pCL79 (pYL15-Gb_33836)	pUC ori, Km^R, M13 primer sites; Gb_33836.	This study
pCL87 (pESC-URA-TcGGPPs-GbLPS)	2µ (episomal) URA3, P_{GAL10}–*TcGGPPs*–T_{ADH1}; P_{GAL1}–*LPS (ΔN79, M593I/Y700F)*–T_{CYC1}.	This study
pCL88 (pESC-LEU-GbCPR1)	2µ (episomal) LEU2, P_{GAL10}–*GbCPR1*–T_{ADH1}.	This study
pCL89 (pESC-LEU-GbCPR2)	2µ (episomal) LEU2, P_{GAL10}–*GbCPR2*–T_{ADH1}.	This study
pCL90 (pESC-LEU-GbCPR1-Gb_04669)	2µ (episomal) LEU2, P_{GAL10}–*GbCPR1*–T_{ADH1}; P_{GAL1}–*Gb_04669*–T_{CYC1}.	This study
pCL91 (pESC-LEU-GbCPR2-Gb_04669)	2µ (episomal) LEU2, P_{GAL10}–*GbCPR2*–T_{ADH1}; P_{GAL1}–*Gb_04669*–T_{CYC1}.	This study
pCL92 (pESC-LEU-AtCPR1-Gb_04669)	2µ (episomal) LEU2, P_{GAL10}–*AtCPR1*–T_{ADH1}; P_{GAL1}–*Gb_04669*–T_{CYC1}.	This study
pAG415GPD-ccdB	Centromeric LEU2, *attR1*–P_{GPD}–*ccdB*–T_{CYC1}–*attR2*.	(Alberti et al., 2007)
pCL186 (pAG415GPDGbLPS)	Centromeric LEU2, P_{GPD}–*GbLPS*–T_{ADH1}.	This study
pCL248 (pESC-HIS-Gb_33836)	2µ (episomal) HIS3, P_{GAL10}–*Gb_33836*–T_{ADH1}.	This study
pCL334 (pESC-HIS-SmCPS-CfTPS3)	2µ (episomal) HIS3, P_{GAL10}–*SmCPS*–T_{ADH1}; P_{GAL1}–*CfTPS3*–T_{CYC1}.	This study

pCL335 (pESC-HIS-SmCPS-SmKSL1)	2μ (episomal) HIS3, P_{GAL10}–*SmCPS*–T_{ADH1}; P_{GAL1}–*SmKSL1*–T_{CYC1}.	This study
pCL342（pESC-Leu-GbCPR2-Gb33836）	2μ (episomal) LEU2, P_{GAL10}–GbCPR1–T_{ADH1}; P_{GAL1}–*Gb_33836*–T_{CYC1}	This study
pCL418（pESC-Ura-TcGGPPs）	2μ (episomal) URA3, P_{GAL10}–*TcGGPPs*–T_{ADH1};	This study
pCL420 (pYL13-Gb_04669)	Km^R, P_{GPD}–Gb_04669–T_{ADH1}.	This study
pCL433（pESC-His-SmCPS）	2μ (episomal) HIS3, P_{GAL10}–SmCPS–T_{ADH1}	This study

Table S7. Structure and NMR data for compound 7 ($CDCl_3$, 600 MHz for ^1H)

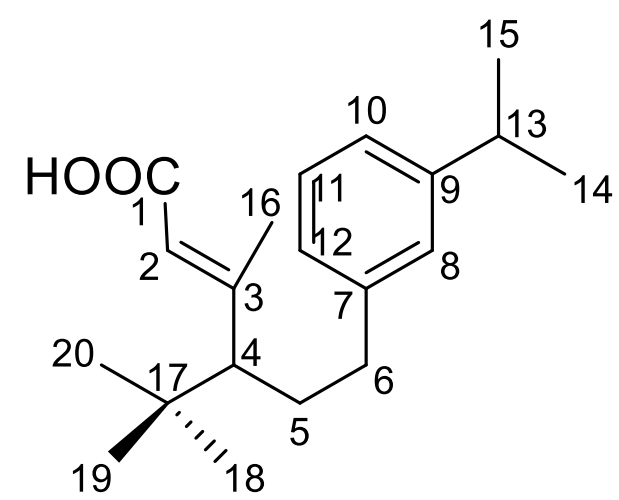

NO.	δH
1	-
2	5.75-5.70, s
3	-
4	1.98-1.93, m
5	1.92-1.87, m; 1.84-1.76, m
6	2.39-2.30, m; 2.57-2.49, t (11.0)
7	-
8	7.01-6.99, s
9	-
10	7.07-7.04, d (7.6)
11	7.23-7.19, t (7.6)
12	6.99-6.96, d (7.6)
13	2.92-2.81, sept (6.9)
14	1.26-1.24, d (6.9)
15	1.26-1.24, d (6.9)
16	2.24-2.19, s
17	-
18	0.93-0.89, s
19	0.93-0.89, s
20	0.93-0.89, s

Table S8. Structure and NMR data for compound 8 ($CDCl_3$, 600 and 151 MHz for ^1H and ^{13}C, respectively)

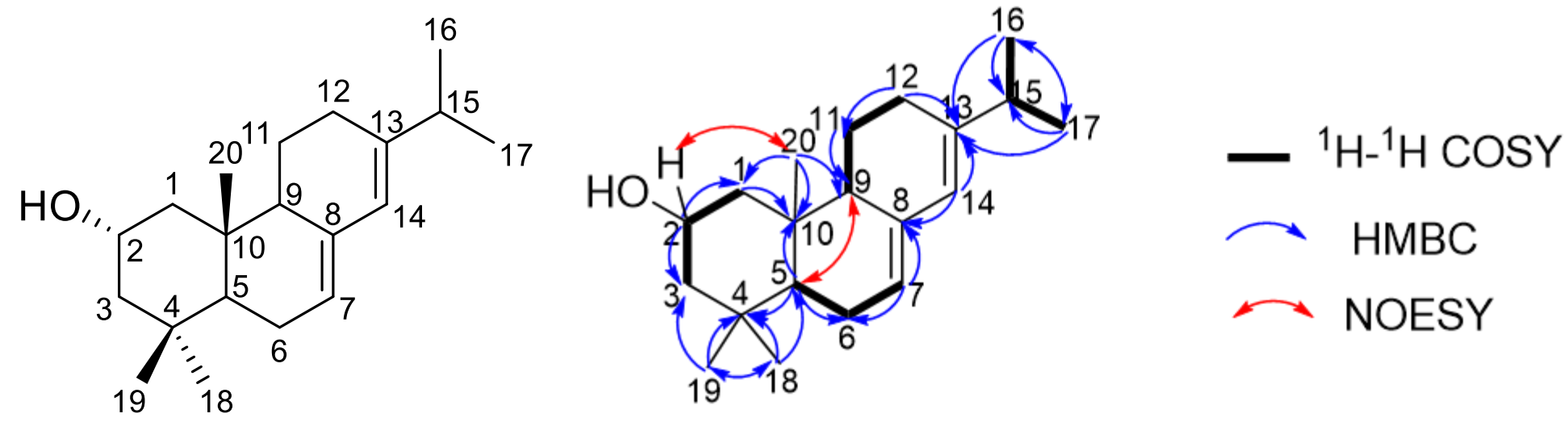

NO.	*δ*H	*δ*C	HMBC	COSY	NOESY
1	2.19-2.15, m; 1.01 – 1.00, m	48.67	C-2, C-10	H-2	
2	3.89, tt (11.5, 4.0)	65.40	-	H-1, 3	H-20
3	1.86-1.73, m; 1.16 t (11.9)	51.49	C-2, 4, 5, 18, 19	H-2	
4	-	34.93	-		
5	1.27-1.21, m	49.78	C-4, 6, 10	H-6	H-9
6	2.19-2.15, m; 1.98-1.90, m	23.96	C-5, 7	H-5, 7	
7	5.44, dd (5.5, 2.7)	121.39	C- 5, 6, 14	H-6	
8	-	135.32	-		
9	1.98-1.90, m	51.13	C-7, 8, 10, 11	H-11	H-5
10	-	37.02	-		
11	1.86-1.73, m; 1.27-1.21, m	22.90	C-8, 9, 12, 13	H-9, 12	
12	2.16-2.02, m	27.58	C-9, 11, 13, 14	H-11	
13	-	145.49	-		
14	5.79, s	122.43	C-7, 8, 9, 12, 15		
15	2.23, dq (13.7, 6.6)	35.02	C-12, 13, 14, 16, 17	H-16, 17	
16	1.02, d (5.4)	21.56	C-13, 15, 17	H-15	
17	1.00, d (5.5)	21.00	C-13, 15, 16	H-15	
18	0.93, s	33.41	C-3, 4, 5, 19		
19	0.96, s	22.98	C-3, 4, 5, 18		
20	0.83, s	14.74	C-1, 9, 10		H-2

Table S9. NMR data for compound 10 ($CDCl_3$, 600 and 151 MHz for ^1H and ^{13}C, respectively)

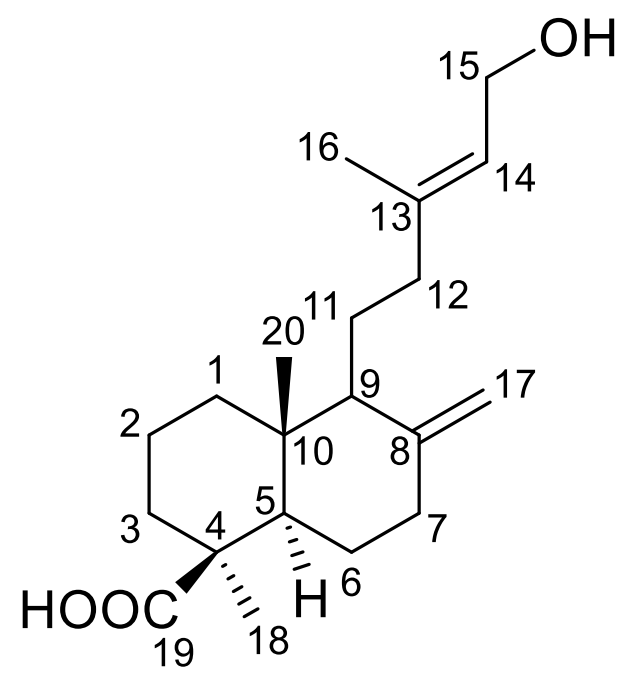

No.	δH	δC
1	1.05, td (13.4, 4.1); 1.93–1.80, m	38.84
2	1.65–1.49, m; 1.93–1.80, m	38.16
3	1.05, td (13.4, 4.1); 2.18–2.12, m	20.03
4	-	44.24
5	1.33, dd (11.9)	55.62
6	1.93–1.80, m; 2.02–1.95, m	26.21
7	1.93–1.80, m; 2.45–2.37, m	39.25
8	-	148.05
9	1.65–1.49, m	56.38
10	-	40.57
11	1.65–1.49, m	22.09
12	1.93–1.80, m; 2.18–2.12, m	38.54
13	-	140.66
14	5.38, t, (7.0)	123.18
15	4.15, d (7.0)	59.58
16	1.67, s	16.51
17	4.86, d (1.6); 4.53, s	106.67
18	1.24, s	29.12
19	-	182.19
20	0.60, s	12.96

Table S10. Structure and NMR data for compound 11 ($CDCl_3$, 600 and 151 MHz for ^1H and ^{13}C, respectively)

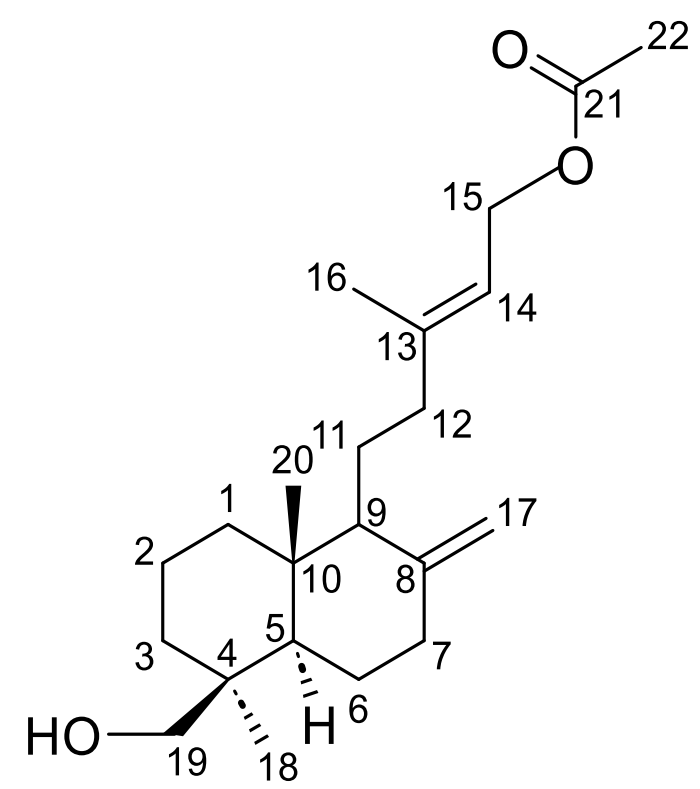

NO.	*δ*H	*δ*C	HMBC	COSY	NOESY
1	1.83-1.76, m; 1.04, dt (12.6, 6.3)	39.14	C-2, 10, 20	H-2	
2	1.56-1.46, m	19.14	C-1, 3	H-1, 3	
3	1.85-1.81, m; 1.03-0.93, m	35.56	C-2, 4, 5, 18	H-2	
4	-	39.01	-	-	
5	1.26, d (2.5)	56.41	C-4, 6, 10, 19, 20	H-6	H-18
6	1.83-1.76, m; 1.32, dq (12.8, 4.1)	24.59	C-5, 7	H-5, 7	
7	2.38, ddd (12.8, 4.1, 2.4); 1.93, td (12.7, 5.1)	38.75	C-6, 8, 9, 17	H-6	
8	-	148.16	-	-	
9	1.60-1.56, m	56.46	C-8, 10, 17	H-11	
10	-	39.67	-	-	
11	1.66-1.59, m; 1.47-1.38, m	21.24	C-8, 9, 10, 12, 13	H-9, 12	
12	2.16, td (14.1, 12.3, 4.5); 1.87-1.83, m	38.45	C-9, 11, 13, 14, 16	H-11	
13	-	143.13	-	-	
14	5.33-5.28, m	118.17	C-12, 15, 16	H-15, 16	
15	4.58, d (7.1)	61.57	C-13, 14, 21	H-14	
16	1.69, d (1.2)	16.68	C-12, 13, 14	H-14	
17	4.82, d (1.6); 4.51, d (1.8)	106.73	C-7, 9	-	
18	0.98, s	27.22	C-3, 4, 5, 19	-	H-5
19	3.75, d (10.8); 3.39, d (10.8)	65.23	C-3, 4, 5, 18	-	
20	0.65, s	15.47	C-1, 9, 10	-	
21	-	171.29	-	-	
22	2.06, s	21.86	C-15, 21	-	

Table S11. Absolute RT–qPCR-derived transcript abundance of Gb_04669 and Gb_33836 in three *Ginkgo biloba* tissues.

Gene	Plant	Tissue	Mean Ct	RNA input (µg)	cDNA template volume (µL)	Target copies/ µL cDNA	Target copies/ reaction	Target copies/ µg total RNA	$\log_{10}$(target copies/ µg total RNA)
Gb_04669	1	Leaf	28.192	1.000000	2.0	146.7	293.4	2,934.1	3.4675
Gb_04669	1	Sarcotesta	30.019	0.335720	2.0	41.2	82.4	2,455.5	3.3901
Gb_04669	1	Stem	26.320	0.375914	2.0	538.4	1,076.8	28,644.8	4.4570
Gb_04669	2	Leaf	28.760	1.000000	2.0	98.8	197.6	1,976.5	3.2959
Gb_04669	2	Sarcotesta	29.863	0.432376	2.0	45.9	91.8	2,124.2	3.3272
Gb_04669	2	Stem	27.366	0.580132	2.0	260.3	520.5	8,972.6	3.9529
Gb_04669	3	Leaf	28.671	1.000000	2.0	105.1	210.2	2,102.4	3.3227
Gb_04669	3	Sarcotesta	29.967	0.367108	2.0	42.7	85.5	2,327.8	3.3669
Gb_04669	3	Stem	26.792	0.512344	2.0	388.0	776.0	15,145.9	4.1803
Gb_33836	1	Leaf	26.982	0.300000	1.0	7,124.4	7,124.4	474,957.6	5.6767
Gb_33836	1	Sarcotesta	28.009	0.300000	1.0	3,450.7	3,450.7	230,048.5	5.3618
Gb_33836	1	Stem	25.244	0.300000	1.0	24,304.5	24,304.5	1,620,303.1	6.2096

Gene	Plant	Tissue	Mean Ct	RNA input (µg)	cDNA template volume (µL)	Target copies/ µL cDNA	Target copies/ reaction	Target copies/ µg total RNA	$\log_{10}$(target copies/ µg total RNA)
Gb_33836	2	Leaf	27.215	0.300000	1.0	6,042.8	6,042.8	402,852.0	5.6051
Gb_33836	2	Sarcotesta	28.160	0.300000	1.0	3,100.3	3,100.3	206,688.6	5.3153
Gb_33836	2	Stem	26.654	0.300000	1.0	8,979.0	8,979.0	598,596.9	5.7771
Gb_33836	3	Leaf	26.940	0.300000	1.0	7,337.0	7,337.0	489,132.7	5.6894
Gb_33836	3	Sarcotesta	28.024	0.300000	1.0	3,413.1	3,413.1	227,538.1	5.3571
Gb_33836	3	Stem	25.732	0.300000	1.0	17,217.6	17,217.6	1,147,839.0	6.0599

Table S12. Quantification of isocupressic acid in three independent Gb_33836-expressing yeast cultures.

**Independent culture**	**Peak area**	**$C_{extract}$ (μg mL^{-1})**	**Titer (μg L^{-1})**
1	4,914,068	30.91	206.04
2	4,897,055	30.74	204.92
3	5,168,144	33.41	222.75
**Mean ± SD**	4,993,089 ± 151,841	31.69 ± 1.50	211.24 ± 9.98

Reference

ALBERTI, S., GITLER, A. D. & LINDQUIST, S. 2007. A suite of Gateway cloning vectors for high-throughput genetic analysis in Saccharomyces cerevisiae. *Yeast,* 24**,** 913-9.

ENTIAN, K.-D. & KöTTER, P. 2007. 25 Yeast Genetic Strain and Plasmid Collections. *In:* STANSFIELD, I. & STARK, M. J. R. (eds.) *Methods in Microbiology.* Academic Press.

GAO, K., XU, K., LIN, P., ZHU, J., WU, R. & ZI, J. 2024. Unexpected Main-Chain-Mediated and Neutral-Intermediate-Involved Catalytic Reaction in a High-Fidelity ent-Neoabietadiene Synthase. *ACS Catalysis,* 14**,** 14233-14241.

MA, T., SHI, B., YE, Z., LI, X., LIU, M., CHEN, Y., XIA, J., NIELSEN, J., DENG, Z. & LIU, T. 2019. Lipid engineering combined with systematic metabolic engineering of Saccharomyces cerevisiae for high-yield production of lycopene. *Metabolic Engineering,* 52**,** 134-142.

SIEMON, T., WANG, Z., BIAN, G., SEITZ, T., YE, Z., LU, Y., CHENG, S., DING, Y., HUANG, Y., DENG, Z., LIU, T. & CHRISTMANN, M. 2020. Semisynthesis of Plant-Derived Englerin A Enabled by Microbe Engineering of Guaia-6,10(14)-diene as Building Block. *Journal of the American Chemical Society,* 142**,** 2760-2765.
